# Meta-Analysis of Early Nonmotor Features and Risk Factors for Parkinson Disease

**DOI:** 10.1002/ana.23687

**Published:** 2012-10-15

**Authors:** Alastair J Noyce, Jonathan P Bestwick, Laura Silveira-Moriyama, Christopher H Hawkes, Gavin Giovannoni, Andrew J Lees, Anette Schrag

**Affiliations:** 1 Institute of Neurology, University College LondonLondon, United Kingdom; 2 Blizard Institute of Cell and Molecular Science, Barts and the London School of Medicine and Dentistry, Queen Mary University of LondonLondon, United Kingdom; 3 Wolfson Institute of Preventive Medicine, Barts and the London School of Medicine and Dentistry, Queen Mary University of LondonLondon, United Kingdom; 4 Department of Neurology, State University of CampinasCampinas, Brazil

## Abstract

**Objective:**

To evaluate the association between diagnosis of Parkinson disease (PD) and risk factors or early symptoms amenable to population-based screening.

**Methods:**

A systematic review and meta-analysis of risk factors for PD.

**Results:**

The strongest associations with later diagnosis of PD were found for having a first-degree or any relative with PD (odds ratio [OR], 3.23; 95% confidence interval [CI], 2.65–3.93 and OR, 4.45; 95% CI, 3.39–5.83) or any relative with tremor (OR, 2.74; 95% CI, 2.10–3.57), constipation (relative risk [RR], 2.34; 95% CI, 1.55–3.53), or lack of smoking history (current vs never: RR, 0.44; 95% CI, 0.39–0.50), each at least doubling the risk of PD. Further positive significant associations were found for history of anxiety or depression, pesticide exposure, head injury, rural living, beta-blockers, farming occupation, and well-water drinking, and negative significant associations were found for coffee drinking, hypertension, nonsteroidal anti-inflammatory drugs, calcium channel blockers, and alcohol, but not for diabetes mellitus, cancer, oral contraceptive pill use, surgical menopause, hormone replacement therapy, statins, acetaminophen/paracetamol, aspirin, tea drinking, history of general anesthesia, or gastric ulcers. In the systematic review, additional associations included negative associations with raised serum urate, and single studies or studies with conflicting results.

**Interpretation:**

The strongest risk factors associated with later PD diagnosis are having a family history of PD or tremor, a history of constipation, and lack of smoking history. Further factors also but less strongly contribute to risk of PD diagnosis or, as some premotor symptoms, require further standardized studies to demonstrate the magnitude of risk associated with them. ANN NEUROL 2012

Idiopathic Parkinson disease (PD) has a prevalence of approximately 1% in individuals aged >65 years.^1^–^3^ The cause is unknown, but many individual risk factors for subsequent PD have been suggested. In addition, several early nonmotor features have been identified that precede the diagnosis of PD, sometimes by several years.^4^–^8^ In the past 10 years, there have been a small number of meta-analyses addressing risk factors for subsequent PD, including smoking, coffee consumption, pesticide exposure, and use of nonsteroidal anti-inflammatory drugs (NSAIDs).^9^–^21^ No review has comprehensively assessed predictive risk factors for later diagnosis of PD. Here we report the largest and most comprehensive systematic review and meta-analysis to date, which uses an extensive search of observational studies to calculate effect sizes of multiple risk factors for PD.

## Methods

### Search Strategy

We followed PRISMA 2009 guidelines for systematic review and meta-analysis and the Cochrane Collaboration definition of both terms.^22^, ^23^ We undertook a MEDLINE database search on PubMed from 1966 until March 2011 for studies that reported factors that could be used to screen for risk of future PD. The MeSH terms used were: Constipation OR Sleep Disorders OR Olfaction Disorders OR Smoking OR Color Vision OR Coffee OR Erectile Dysfunction OR Depression OR Anxiety OR Mood Disorders OR Hydroxymethylglutaryl-CoA Reductase Inhibitors OR Anti-Inflammatory Agents, Non-Steroidal OR Solvents OR Pesticides OR Body Mass Index OR Family OR Risk OR Risk Factors AND Parkinson Disease. We restricted our analysis to articles written in English. Reference lists of suitable retrieved articles were hand searched for any missed references, as were the reference lists of existing relevant meta-analyses identified in the original search. The final search was carried out on March 31, 2011.

### Inclusion Criteria

Published studies were included if they fulfilled the following criteria: (1) assessed at least 1 risk factor or early nonmotor symptom preceding a subsequent diagnosis of PD; (2) reported original data on relative risks (RRs) or odds ratios (ORs) from cohorts representative of the general population or case–control studies with cases defined as patients diagnosed with PD; and (3) reported data that could be easily obtained in a primary care environment, that is, those factors that could be determined through questionnaires or widely available blood tests.

### Exclusion Criteria

We excluded review articles, editorials, commentaries, hypothesis papers, letters that reported no new data, meta-analyses, and abstracts. We excluded studies that (1) reported on treatment and management of PD, (2) considered associations with established PD (ie, not preceding PD), (3) reported factors not easily ascertainable in the primary care setting (eg, complicated questionnaires on food frequencies, life events, physical activity, environmental, solvent or toxin exposures), (4) studied young onset PD only, (5) did not use a control group or provide adequate details of the control group (including prevalence studies), (6) used blood relatives as the control group, (7) were twin studies, (8) were genetic studies or laboratory studies not used widely, (9) reported on the same risk factor in a common study population (where >1 paper reported on the same population, we chose the larger and, where equal, the most recent report), (10) reported on a disease other than PD, or (11) reported measures other than OR/RR or an equivalent (such as proportional mortality rate and standardized hospitalization rate) or from which an OR could not be calculated. If there was disagreement between authors (A.J.N., J.P.B., A.S.), the articles were discussed in further detail until an agreement was reached.

### Data Extraction

Study characteristics, a risk estimate of the main study finding, and secondary findings were extracted for all eligible studies using a standardized template. Only factors for which a significant association was reported in at least 1 study were included. We included risk factors according to binary measurements (eg, yes vs no for having a first-degree relative with PD and ever versus never for alcohol). Data that reported exposures as quartiles or quintiles where the lowest exposure quartile was equal to zero were converted to binary terms. We excluded associations reported with medical conditions, drugs or toxins known to cause symptomatic Parkinsonism such as antipsychotics or carbon monoxide poisoning. We did not include studies that reported associations with dementia occurring before onset of PD, as these cases would not fulfill current criteria for PD and might include cases of dementia with Lewy bodies.^24^ For cases, we used figures for PD instead of Parkinsonism. In studies that reported data for both young onset and typical age of onset PD, we excluded the young onset data where possible. If case–control studies made comparison with >1 control group, the control group most representative of the healthy general population was used. If studies did not report OR, RR, or an equivalent measure, the raw data were reviewed to determine whether ORs could be calculated. In studies that reported both crude ORs and adjusted ORs, the adjusted figures were used. After application of the above methods, no additional studies required exclusion for quality reasons, according to Newcastle Ottawa Scale guidelines.^25^ Length of time that any given factor preceded onset of PD was not included in the analysis due to inconsistent reporting of these data.

### Statistical Analysis

Where a factor of interest was reported by 2 or more studies in a consistent manner, these were combined in a meta-analysis; first separately for case–control and cohort studies (given that cohort studies are less subject to bias), and second for all studies together (considering odds ratios from case–control studies to be estimates of relative risks) to generate a pooled effect size and 95% confidence interval (CI) for each factor. Heterogeneity between studies was assessed using the I^2^ statistic and, where statistically significant heterogeneity was found (*p* < 0.05), the random effects model was used to combine results.^26^, ^27^ Publication bias was assessed using the Egger test, and where statistically significant bias was found, the trim and fill method was used to adjust for it.^28^, ^29^ Where data were not given in a way that could be used in the meta-analysis or where only 1 significant study was identified for a given risk factor, the findings of these studies are only listed in this review. All analyses were performed using Stata version 10 (StataCorp, College Station, TX).

## Results

**FIGURE 1:**
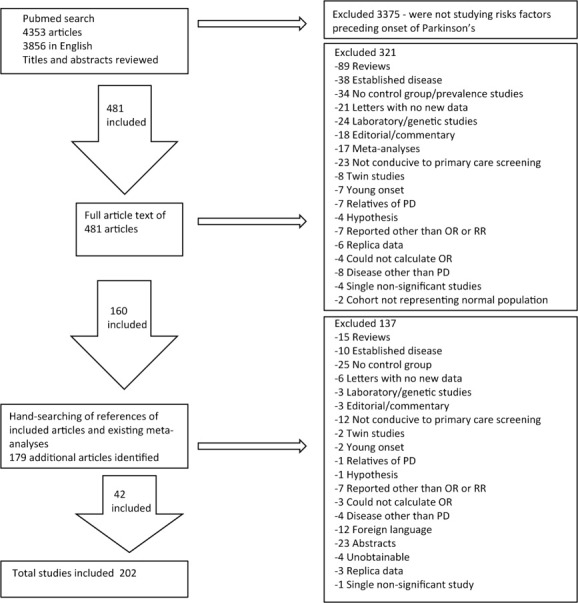
Flowchart of studies included and excluded. OR = odds ratio; PD = Parkinson disease; RR = relative risk.

The literature search yielded 3,856 English language articles, of which 202 were eligible for inclusion in the systematic review and 173 of these in the meta-analysis (Fig 1). The reasons for exclusion of other studies are given. Full details of studies included in the meta-analysis are provided (Supplementary Tables 1 and 2). Twenty-nine articles that could not be included in the meta-analysis, primarily due to inconsistencies in method of measurement of the studied risk factor or because only 1 study had reported a significant result on a given factor, are described and shown (Supplementary Table 3).

**FIGURE 2:**
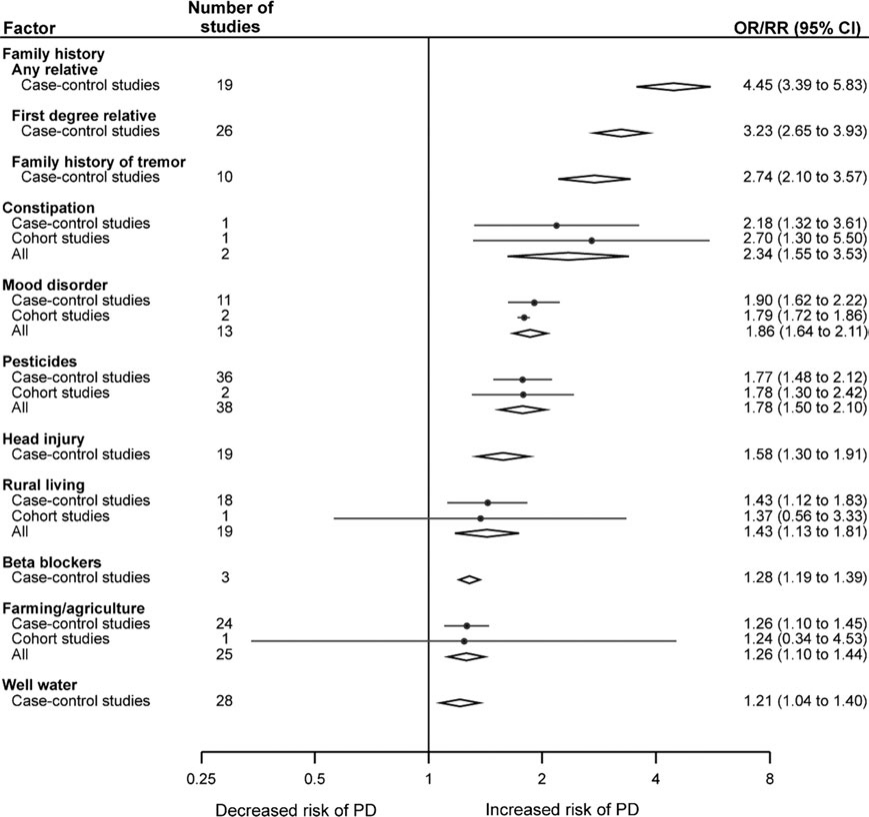
Factors included for meta-analysis that show a significant positive association with a later diagnosis of Parkinson disease (PD). CI = confidence interval; RR = relative risk; OR = odds ratio.

**FIGURE 3:**
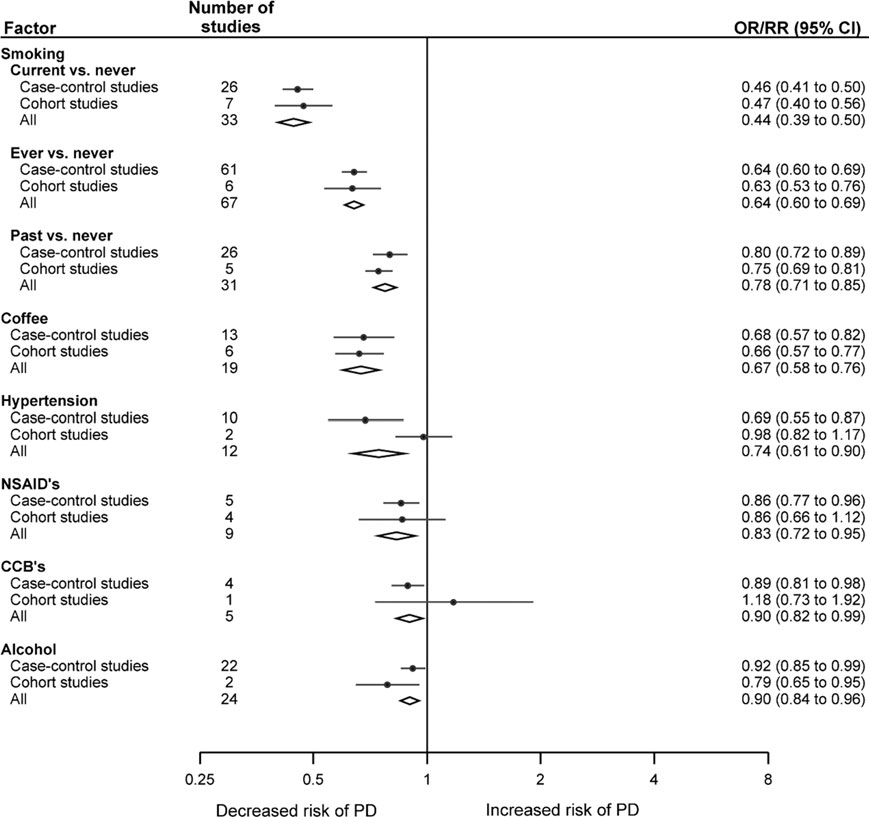
Factors included for meta-analysis that show a significant negative association with a later diagnosis of Parkinson disease (PD). CCB's = calcium channel blockers; CI = confidence interval; NSAID's = nonsteroidal anti-inflammatory drugs; PD = Parkinson disease; RR = relative risk; OR = odds ratio.

**FIGURE 4:**
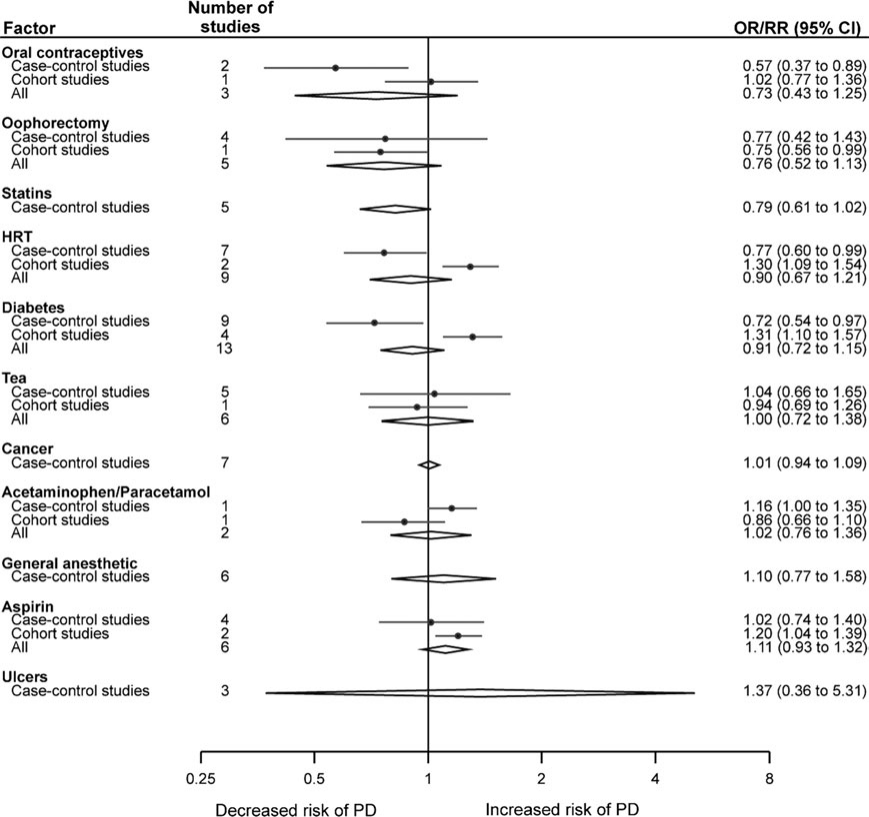
Factors included for meta-analysis that did not show a significant association with a later diagnosis of Parkinson disease. CI = confidence interval; HRT = hormone replacement therapy; RR = relative risk; OR = odds ratio.

Summary results for positive and negative associations with PD found in the meta-analysis and those where no association was found are shown in Figures 2 to 4. Significant positive associations were found for family history of PD (including any relative and first-degree relatives only), family history of tremor, preceding constipation, prior mood disorder, exposure to pesticides (or herbicides or insecticides), previous head injury, rural living, beta-blocker use, farming/agricultural occupation, and well water drinking. Significant negative associations were found for smoking, coffee drinking, prior hypertension, use of NSAIDs, calcium channel blocker use, and alcohol consumption. No significant association was found for tea drinking; oral contraceptive pill use, preceding oophorectomy, or hormone replacement therapy; preceding diabetes mellitus, cancer, or gastric ulcer; use of statins, acetaminophen/paracetamol, or aspirin; and prior general anesthetic. In the systematic review, additional associations that were reported but could not be categorized consistently included negative associations with previously raised serum urate and conflicting results with serum cholesterol (total and various subtypes of serum lipid), obesity, physical activity, any antihypertensive medication (without further subclassification), education, and various occupations. Single studies also reported negative associations with both parents having smoked and use of smokeless tobacco and positive associations with family history of any neurologic disease, hyposmia, erectile dysfunction, and excessive daytime sleepiness; complaints of stiffness, imbalance, or tremor; having a first-degree relative with melanoma; having brown, blond, and red hair versus black hair; a variety of infectious diseases; immediate-type hypersensitivity; anemia; duration of fertile life and cumulative length of pregnancies; having ≥3 children; having no children, and various occupations (see Supplementary Table 3).

### Assessment of Publication Bias

According to the Egger test, there was evidence of publication bias for the factors ever smoking (*p* = 0.017), coffee drinking (*p* = 0.002), pesticides (*p* < 0.001), hormone replacement therapy (p = 0.016), statins (*p* = 0.038), any family history of PD (*p* = 0.011), family history of tremor (*p* = 0.009), and well water drinking (*p* = 0.005). Using the trim and fill method to account for the bias had no effect on the summary estimate for ever smoking, coffee, hormone replacement therapy or statins, but did diminish the summary estimates for pesticides (RR from 1.78 to 1.53; 95% CI, 1.29–1.80), any family history of PD (OR from 4.45 to 3.25; 95% CI, 2.43–4.35), family history of tremor (OR from 2.74 to 2.51; 95% CI, 1.96–3.22), and well water drinking (OR from 1.21 to 1.20; 95% CI, 1.04–1.39); however, the conclusion that these are statistically significant risk factors for PD was not altered.

## Discussion

This systematic review identified >40 individual risk factors of potential value for clinical screening. Identified factors include genetic and environmental risk factors, associated comorbidities, and medication exposures, as well as early nonmotor features that may represent the earliest stages of PD. Although some of these have possible pathogenic importance and others may represent early PD, they serve as markers that may help identify patients who are at high risk of a future diagnosis of PD. They also provide important information for the earlier diagnosis of PD, which is often delayed by several years.^30^ Of the 30 factors that had data amenable to meta-analysis, 19 significantly altered risk of future PD and 11 did not reach significance. This information may be used to provide the basis for primary care or population-based screening as well as improve our understanding of contribution of risk to subsequent PD for each of these factors.

### Family History

Having a family member with PD was the strongest risk factor for later diagnosis of PD. Although a number of monogenic causes of PD have been identified, these account for only about 5% of PD. The excess risk in those with a family history of PD may be conveyed by multiple susceptibility loci.^31^ Familial aggregation may also be explained by the effect of a shared environment, for example, concordance for not smoking and avoidance of coffee, which may influence the risk of PD, either independently or in conjunction with genetic susceptibility.

### Lifestyle and Environmental Factors

A history of smoking reduces the risk of PD by about 36%, with coffee and alcohol (but not tea) consumption also reducing risk. For smoking, the effect is strongest in current smokers and weakest in past smokers (56% for current smokers and 22% for past smokers), but the association remains significant in all. The nature of this association is still poorly understood, and cannot be explained simply by selection bias and confounding.^32^–^36^ The risk reduction from ever versus never coffee use and ever versus never alcohol use is approximately 33% and 10%, respectively. A confounding effect of smoking, coffee, and alcohol use in combination has been noted, but other investigators suggest independence of these factors.^37^, ^38^

Pesticide and environmental toxin exposure have been often implicated in PD causation.^13^, ^18^, ^19^, ^39^ Despite differences in populations studied and study design, we found an overall positive association with ever exposure to pesticides. Some suggest there may be a greater risk associated with length of exposure to pesticides, but we did not evaluate this in our analysis.^18^, ^19^ The finding that farming is also associated with subsequent PD may be in part due to increased likelihood of exposure to pesticides and other chemicals. Inconsistencies in adjustment between studies made it impossible to determine the importance of this interaction. Rural living and well water exposure were also significant positive associations. We did not include additional surrogates for pesticide or chemical exposures such as gardening, plantation work, and solvent exposures requiring more detailed exposure and lifestyle questionnaires. Other occupations that have been reported to change the risk of PD (but could not be classified consistently) included being a physician, clerk, carpenter, or cleaner or having a legal occupation (increase); production worker, driver, technician, transport/communication worker, mechanical/factory or metal worker, sales or service personnel, or engineer (reduced); or construction/extraction worker (conflicting results).

Head injury may carry increased risk of PD, particularly in those with repeated head injury and pugilist's encephalopathy.^40^ In our meta-analysis, head injury with or without loss of consciousness had a significant but modest effect on the risk of subsequent diagnosis of PD.

### Early Symptoms

This systematic review reaffirms the proposed early nonmotor symptoms, which may predate the diagnosis of PD by several years. Constipation and mood disorders appear to approximately double an individual's risk of subsequent PD in the meta-analysis. Both constipation and mood disorders have been suggested to correlate with brainstem involvement that, together with olfactory bulb involvement, occurs early in PD and later spreads to the substantia nigra and to the cortex.^41^ This theory would also support a role for hyposmia, erectile dysfunction, and excessive daytime somnolence as early features of PD, all of which have been reported in single prospective cohort studies to be associated with significantly increased risk.^42^–^44^ These were large, well-conducted studies, and there is additional support for smell loss preceding PD in studies of first-degree relatives of persons with PD and idiopathic anosmia (see Hawkes^5^). However, these clinical syndromes are not routinely tested and are infrequently recorded; therefore, no case–control or further cohort studies fulfilling our criteria that allow meta-analysis are available at the present time. Rapid eye movement sleep behavior disorder (RBD) is also believed to precede PD by a number of years.^45^, ^46^ Currently, no observational studies that give ORs or RRs for this association have been reported. Further studies are required to replicate (for hyposmia, erectile dysfunction, and excessive daytime sleepiness) or delineate (for RBD) the magnitude of risk each of these conveys and to establish a comparable method of measurement for each. The association of a preceding tremor, imbalance, and stiffness with later PD (all reported in single studies) may point to early motor manifestations of the disease before a diagnosis can be made.^47^, ^48^

### Comorbidities and Medications

Hypertension was associated with reduced risk of PD, but the role of selective mortality in these individuals has not been studied. The combined analysis of studies of calcium channel blockers suggests a mild but significant overall reduction of PD risk. Beta blockers, on the other hand, were associated with increased PD risk. This could, however, potentially be explained by trials of beta blockers in those with seemingly isolated tremor. A history of diabetes mellitus did not significantly alter risk; however, further studies may be necessary, given that the case–control studies showed a statistically significant decrease in risk, whereas the cohort studies, showed a statistically significant increase in risk. Statins showed a trend of reduction in risk of subsequent PD in this meta-analysis, which may be due to a protective role through reduction of oxidative stress.^49^ Hypercholesterolemia, obesity, and physical activity could not be included in the meta-analysis due to differences in assessment, and the systematic review found conflicting results.

NSAIDs are associated with risk reduction by approximately 17%. A similar finding has recently been published in a Cochrane review of NSAIDs preceding PD diagnosis.^50^ There is increasing evidence that inflammation may play a role in the pathogenesis of PD, which may underlie this finding.^31^, ^51^, ^52^ Aspirin and acetaminophen/paracetamol did not modify risk significantly. The suggestion that raised plasma urate might protect against PD may also be related to inflammatory mechanisms, because serum urate is a free radical scavenger and therefore protects against oxidative stress, which may contribute to dopaminergic neuronal loss.^53^

We did not find any association of subsequent PD with the oral contraceptive pill, surgical menopause, or hormone replacement therapy, although further studies on the latter are needed, given that case–control studies showed a statistically significant decrease in risk, whereas cohort studies showed a statistically significant increase in risk. There was no significant association of PD and preceding cancer. Although we did not include melanoma as a specific search term, a recent meta-analysis suggests a positive association of established PD with melanoma, but no association if melanoma precedes a diagnosis of PD.^54^

*Helicobacter pylori* infection has been suggested as a risk factor for PD in recent years, but there are no studies that specifically look at *H. pylori* and subsequent risk of PD. A surrogate marker for this could be gastric ulceration, but in our meta-analysis there was no association with prior gastric ulcer and subsequent PD diagnosis.

### Factors Not Included in Analysis

Age and gender were not included in this review, as they are mainly reported in uncontrolled prevalence studies. Thus, although some of the studies included in the analysis reported risk associated with these variables, a combined risk estimate is unlikely to be representative of the full published literature on these factors. Nevertheless, the increased prevalence rate with increasing age and the higher prevalence in men are well recognized, and reporting is therefore usually stratified by age and gender.^2^, ^55^

### Limitations of the Analysis

We restricted our search to articles written in English, and therefore reports written in other languages are not included in the analysis. Due to the large number of studies used and the heterogeneity of methods and reported findings, we selected variables according to the main criterion that they can be screened for on a population basis. Therefore, risk factors and early symptoms not easily screened for would not have been included. Not all studies reported estimates of risk adjusted for confounders (eg, smoking). Where such adjustments have been made, we have included these data in the analysis.

Risk factors were dichotomized, which ignores dose effects. However, an essential feature of this analysis was to define factors that could be defined in binary terms, to allow them to be employed in combination for large-scale screening.

Statistically significant heterogeneity was found in the majority (24 of 30) of meta-analyses performed, similar to what has previously been reported.^56^ In 16 of these there was moderate heterogeneity (I^2^ = 50–75%), and in 4 there was high heterogeneity (I^2^ > 75%). This is expected, because of the differences between individual studies in, for example, case ascertainment, study population characteristics, exposure measurement, and whether crude or adjusted risk estimates were reported. For this reason, the pooled estimates should be interpreted cautiously, in particular for those instances of high heterogeneity (hypertension, statin use, previous gastric ulceration, and rural living).

### Conclusions

This is the first comprehensive systematic review and meta-analysis taking into account all risk factors for PD suitable for screening in primary care. This report allows comparative assessment of the evidence for, and magnitude of association for, the numerous proposed risk and early disease factors of PD, and helps to understand better their contribution to the risk of PD.
