# Supplementary material for: Meta-Analysis of Early Nonmotor Features and Risk Factors for Parkinson Disease
Source: Ann Neurol. 2012 Oct 15;72(6):893–901. doi: 10.1002/ana.23687 (PMC3556649; doi:10.1002/ana.23687)
Supplement: Supplementary file 1 [file ana0072-0893-SD1.doc]

**Reference list**

1. de Lau LML, Giesbergen PCLM, de Rijk MC, Hofman A, Koudstaal PJ, Breteler MMB. Incidence of parkinsonism and Parkinson disease in a general population: the Rotterdam Study. Neurology 2004;63(7):1240–4.
2. Driver JA, Logroscino G, Gaziano JM, Kurth T. Incidence and remaining lifetime risk of Parkinson disease in advanced age. Neurology 2009;72(5):432–8.
3. Olanow CW, Tatton WG. Etiology and pathogenesis of Parkinson's disease. Annu Rev Neurosci 1999;22:123–44.
4. Gonera EG, van't Hof M, Berger HJ, van Weel C, Horstink MW. Symptoms and duration of the prodromal phase in Parkinson's disease. Mov Disord 1997;12(6):871–6.
5. Hawkes CH. The prodromal phase of sporadic Parkinson's disease: does it exist and if so how long is it? Mov Disord 2008;23(13):1799–807.
6. Savica R, Rocca WA, Ahlskog JE. When does Parkinson disease start? Arch Neurol 2010;67(7):798–801.
7. Siderowf A, Stern MB. Premotor Parkinson's disease: clinical features, detection, and prospects for treatment. Ann Neurol 2008;64(Suppl 2):S139–47.
8. Tolosa E, Gaig C, Santamaría J, Compta Y. Diagnosis and the premotor phase of Parkinson disease. Neurology 2009;72(Suppl 7):S12–20.
9. Allam MF, Del Castillo AS, Navajas RF-C. Parkinson's disease, smoking and family history: meta-analysis. Eur J Neurol 2003;10(1):59–62.
10. Allam MF, Campbell MJ, Del Castillo AS, Navajas RF-C. Parkinson's disease protects against smoking? Behav Neurol 2004;15:65–71.
11. Allam MF, Campbell MJ, Hofman A, Del Castillo AS, Navajas RF-C. Smoking and Parkinson's disease: Systematic review of prospective studies. Mov Disord 2004;19(6):614–21.
12. Bajaj A, Driver JA, Schernhammer ES. Parkinson’s disease and cancer risk: a systematic review and meta-analysis. Cancer Causes Control 2010;21(5):697–707.
13. Brown TP, Rumsby PC, Capleton AC, Rushton L, Levy LS. Pesticides and Parkinson’s Disease—Is there a Link? Environ Health Perspect 2006;114(2):156–64.
14. Etminan M, Gill SS, Samii A. Intake of vitamin E, vitamin C, and carotenoids and the risk of Parkinson's disease: a meta-analysis. Lancet Neurol 2005;4(6):362–5.
15. Gagne JJ, Power MC. Anti-inflammatory drugs and risk of Parkinson disease: a meta-analysis. Neurology 2010;74(12):995–1002.
16. Hernán MA, Takkouche B, Caamano-Isorna F, Gestal-Otero JJ. A meta-analysis of coffee drinking, cigarette smoking, and the risk of Parkinson's disease. Ann Neurol 2002;52(3):276–84.
17. Ishihara L, Brayne C. A systematic review of depression and mental illness preceding Parkinson's disease. Acta Neurol Scand 2006;113(4):211–20.
18. Li AA, Mink PJ, McIntosh LJ, Teta MJ, Finley B. Evaluation of epidemiologic and animal data associating pesticides with Parkinson's disease. J Occup Environ Med 2005;47(10):1059–87.
19. Priyadarshi A, Khuder SA, Schaub EA, Shrivastava S. A meta-analysis of Parkinson's disease and exposure to pesticides. NeuroToxicology 2000;21(4):435–40.
20. Priyadarshi A. Environmental risk factors and Parkinson's disease: A meta-analysis. Environ Res 2001;86(2):122–7.
21. Ritz B, Ascherio A, Checkoway H, Marder KS, Nelson LM, Rocca WA, et al. Pooled analysis of tobacco use and risk of Parkinson disease. Arch Neurol 2007;64(7):990–7.
22. Moher D, Liberati A, Tetzlaff J, Altman DG, PRISMA Group. Preferred reporting items for systematic reviews and meta-analyses: the PRISMA statement. BMJ 2009;339:b2535.
23. Green S, McDonald S. Cochrane Collaboration: more than systematic reviews? Intern Med J 2005;35(1):3–4.
24. McKeith IG, Dickson DW, Lowe J, Emre M, O'Brien JT, Feldman H, et al. Diagnosis and management of dementia with Lewy bodies: third report of the DLB Consortium. Neurology 2005;65:1863–72.
25. Wells G, Shea B, O'connell D. The Newcastle-Ottawa Scale (NOS) for assessing the quality of nonrandomised studies in meta-analyses. Ottawa Health Research Institute. 2003.
26. Higgins JPT, Thompson SG, Deeks JJ, Altman DG. Measuring inconsistency in meta-analyses. BMJ 2003;327(7414):557–60.
27. DerSimonian R, Laird N. Meta-analysis in clinical trials. Control Clin Trials 1986;7(3):177–88.
28. Egger M, Smith GD, Phillips AN. Meta-analysis: principles and procedures. BMJ 1997;315(7121):1533–1537.
29. Duval S, Tweedie R. Trim and fill: A simple funnel-plot-based method of testing and adjusting for publication bias in meta-analysis. Biometrics 2000;56(2):455–63.
30. O'Sullivan SS, Williams DR, Gallagher DA, Massey LA, Silveira-Moriyama L, Lees AJ. Nonmotor symptoms as presenting complaints in Parkinson's disease: a clinicopathological study. Mov Disord 2008;23(1):101–6.
31. International Parkinson Disease Genomics Consortium, Nalls MA, Plagnol V, Hernandez DG, Sharma M, Sheerin U-M, et al. Imputation of sequence variants for identification of genetic risks for Parkinson's disease: a meta-analysis of genome-wide association studies. Lancet 2011;377(9766):641–9.
32. Tanner CM, Goldman SM, Aston DA, Ottman R, Ellenberg J, Mayeux R, et al. Smoking and Parkinson's disease in twins. Neurology 2002;58(4):581–8.
33. Wirdefeldt K, Gatz M, Pawitan Y, Pedersen NL. Risk and protective factors for Parkinson's disease: a study in Swedish twins. Ann Neurol 2005;57(1):27–33.
34. Quik M. Smoking, nicotine and Parkinson's disease. Trends Neurosci. 2004 Sep.;27(9):561–568.
35. Fratiglioni L, Wang HX. Smoking and Parkinson's and Alzheimer's disease: review of the epidemiological studies. Behav Brain Res 2000;113:117–20.
36. Morens DM, Grandinetti A, Davis JW, Ross GW, White LR, Reed D. Evidence against the operation of selective mortality in explaining the association between cigarette smoking and reduced occurrence of idiopathic Parkinson disease. Am J Epidemiol 1996;144(4):400–4.
37. Ragonese P, Salemi G, Morgante L, Aridon P, Epifanio A, Buffa D, et al. A case-control study on cigarette, alcohol, and coffee consumption preceding Parkinson's disease. Neuroepidemiology 2003;22(5):297–304.
38. Grandinetti A, Morens DM, Reed D, MacEachern D. Prospective study of cigarette smoking and the risk of developing idiopathic Parkinson's disease. Am J Epidemiol 1994;139(12):1129–38.
39. Langston JW, Ballard P, Tetrud JW, Irwin I. Chronic Parkinsonism in humans due to a product of meperidine-analog synthesis. Science 1983;219(4587):979–80.
40. Parker HL. Traumatic encephalopathy (`Punch Drunk') of professional pugilists. J Neurol Psychopathol 1934;15(57):20–8.
41. Braak H, Del Tredici K, Rüb U, de Vos RAI, Jansen Steur ENH, Braak E. Staging of brain pathology related to sporadic Parkinson's disease. Neurobiol Aging 2003;24(2):197–211.
42. Ross GW, Petrovitch H, Abbott RD, Tanner CM, Popper J, Masaki K, et al. Association of olfactory dysfunction with risk for future Parkinson's disease. Ann Neurol 2008;63(2):167–73.
43. Abbott RD, Ross GW, White LR, Tanner CM, Masaki KH, Nelson JS, et al. Excessive daytime sleepiness and subsequent development of Parkinson disease. Neurology 2005;65(9):1442–6.
44. Gao X, Chen H, Schwarzschild MA, Glasser DB, Logroscino G, Rimm EB, et al. Erectile function and risk of Parkinson's disease. Am J Epidemiol 2007;166(12):1446–50.
45. Postuma RB, Gagnon JF, Vendette M, Fantini ML, Massicotte-Marquez J, Montplaisir J. Quantifying the risk of neurodegenerative disease in idiopathic REM sleep behavior disorder. Neurology 2009;72(15):1296–1300.
46. Schenck CH, Bundlie SR, Mahowald MW. Delayed emergence of a parkinsonian disorder in 38% of 29 older men initially diagnosed with idiopathic rapid eye movement sleep behaviour disorder. Neurology 1996;46(2):388–93.
47. de Lau LML, Koudstaal PJ, Hofman A, Breteler MMB. Subjective complaints precede Parkinson disease: the rotterdam study. Arch Neurol 2006;63(3):362–5.
48. Gao X, Chen H, Schwarzschild MA, Logroscino G, Ascherio A. Perceived imbalance and risk of Parkinson's disease. Mov Disord 2008;23(4):613–6.
49. Becker C, Jick SS, Meier CR. Use of statins and the risk of Parkinson's disease: a retrospective case-control study in the UK. Drug Saf 2008;31(5):399–407.
50. Rees K, Stowe R, Patel S, Ives N, Breen K, Clarke CE, et al. Non-steroidal anti-inflammatory drugs as disease-modifying agents for Parkinson's disease: evidence from observational studies. Cochrane Database Syst Rev 2011;11:CD008454.
51. Hirsch EC, Hunot S. Neuroinflammation in Parkinson's disease: a target for neuroprotection? Lancet Neurol 2009;8(4):382–97.
52. Chen H, O'Reilly EJ, Schwarzschild MA, Ascherio A. Peripheral inflammatory biomarkers and risk of Parkinson's disease. Am J Epidemiol 2007;167(1):90–5.
53. Kutzing MK, Firestein BL. Altered uric acid levels and disease states. J Pharmacol Exp Ther 2008;324(1):1–7.
54. Liu R, Gao X, Lu Y, Chen H. Meta-analysis of the relationship between Parkinson disease and melanoma. Neurology 2011;76(23):2002–9.
55. Wooten GF. Are men at greater risk for Parkinson's disease than women? J Neurol Neurosurg Psychiatry 2004;75(4):637–9.
56. van der Mark M, Brouwer M, Kromhout H, Nijssen P, Huss A, Vermeulen R. Is pesticide use related to Parkinson disease? Some clues to heterogeneity in study results. Environ Health Perspect 2012;120(3):340-7.
57. Alonso ME, Otero E, D'Regules R, Figueroa HH. Parkinson's disease: a genetic study. Can J Neurol Sci 1986;13(3):248–51.
58. Autere JM, Moilanen JS, Myllylä VV, Majamaa K. Familial aggregation of Parkinson's disease in a Finnish population. J Neurol Neurosurg Psychiatry 2000;69(1):107–9.
59. Dick FD, de Palma G, Ahmadi A, Scott NW, Prescott GJ, Bennett J, et al. Environmental risk factors for Parkinson's disease and parkinsonism: the Geoparkinson study. Occup Environ Med 2007;64(10):666–72.
60. Duzcan F, Zencir M, Ozdemir F, Cetin GO, Bagci H, Heutink P, et al. Familial influence on parkinsonism in a rural area of Turkey (Kizilcaboluk-Denizli): a community-based case-control study. Mov Disord 2003;18(7):799–804.
61. Elbaz A, Manubens-Bertran JM, Baldereschi M, Breteler MM, Grigoletto F, Lopez-Pousa S, et al. Parkinson's disease, smoking, and family history. EUROPARKINSON Study Group. J Neurol 2000;247(10):793–8.
62. Herishanu YO, Medvedovski M, Goldsmith JR, Kordysh E. A case-control study of Parkinson's disease in urban population of southern Israel. Can J Neurol Sci 2001;28(2):144–7.
63. Jacob EL, Gatto NM, Thompson A, Bordelon Y, Ritz B. Occurrence of depression and anxiety prior to Parkinson's disease. Parkinsonism Relat Disord 2010;16(9):576–81.
64. Korchounov A, Schipper HI, Preobrazhenskaya IS, Kessler KR, Yakhno NN. Differences in age at onset and familial aggregation between clinical types of idiopathic Parkinson's disease. Mov Disord 2004;19(9):1059–64.
65. Kuopio AM, Marttila RJ, Helenius H, Rinne UK. Familial occurrence of Parkinson's disease in a community-based case-control study. Parkinsonism Relat Disord 2001;7(4):297–303.
66. Kurz M, Alves G, Aarsland D, Larsen JP. Familial Parkinson's disease: a community-based study. Eur J Neurol 2003;10(2):159–63.
67. la Fuente-Fernández de R. Maternal effect on Parkinson's disease. Ann Neurol 2000;48(5):782–7.
68. Marder K, Tang MX, Mejia H, Alfaro B, Cote L, Louis E, et al. Risk of Parkinson's disease among first-degree relatives: A community-based study. Neurology 1996;47(1):155–60.
69. Marder K, Levy G, Louis ED, Mejia-Santana H, Cote L, Andrews H, et al. Familial aggregation of early- and late-onset Parkinson's disease. Ann Neurol 2003;54(4):507–13.
70. Martin WE, Young WI, Anderson VE. Parkinson's disease. A genetic study. Brain 1973;96(3):495–506.
71. McCann SJ, LeCouteur DG, Green AC, Brayne C, Johnson AG, Chan D, et al. The epidemiology of Parkinson's disease in an Australian population. Neuroepidemiology 1998;17(6):310–7.
72. Mickel SF, Broste SK, Hiner BC. Lack of overlap in genetic risks for Alzheimer's disease and Parkinson's disease. Neurology 1997;48(4):942–9.
73. Payami H, Zareparsi S, James D, Nutt J. Familial aggregation of Parkinson disease: a comparative study of early-onset and late-onset disease. Arch Neurol 2002;59(5):848–50.
74. Preux PM, Condet A, Anglade C, Druet-Cabanac M, Debrock C, Macharia W, et al. Parkinson's disease and environmental factors. Matched case-control study in the Limousin region, France. Neuroepidemiology 2000;19(6):333–7.
75. Rocca WA, McDonnell SK, Strain KJ, Bower JH, Ahlskog JE, Elbaz A, et al. Familial aggregation of Parkinson's disease: The Mayo Clinic family study. Ann Neurol 2004;56(4):495–502.
76. Rosen AR, Steenland NK, Hanfelt J, Factor SA, Lah JJ, Levey AI. Evidence of shared risk for Alzheimer’s disease and Parkinson’s disease using family history. Neurogenetics 2007;8(4):263–70.
77. Rybicki BA, Johnson CC, Peterson EL, Kortsha GX, Gorell JM. A family history of Parkinson's disease and its effect on other PD risk factors. Neuroepidemiology 1999;18(5):270–8.
78. Semchuk KM, Love EJ, Lee RG. Parkinson's disease: a test of the multifactorial etiologic hypothesis. Neurology 1993;43(6):1173–80.
79. Shino MY, McGuire V, Van Den Eeden SK, Tanner CM, Popat R, Leimpeter A, et al. Familial aggregation of Parkinson's disease in a multiethnic community-based case-control study. Mov Disord 2010;25(15):2587–94.
80. Spanaki C, Plaitakis A. Bilineal transmission of Parkinson disease on Crete suggests a complex inheritance. Neurology 2004;62(5):815–7.
81. Taylor CA, Saint-Hilaire MH, Cupples LA, Thomas CA, Burchard AE, Feldman RG, et al. Environmental, medical, and family history risk factors for Parkinson's disease: a New England-based case control study. Am J Med Genet 1999;88(6):742–9.
82. Zorzon M, Capus L, Pellegrino A, Cazzato G, Zivadinov R. Familial and environmental risk factors in Parkinson's disease: a case-control study in north-east Italy. Acta Neurol Scand 2002;105(2):77–82.
83. Wang WZ, Fang XH, Cheng XM, Jiang DH, Lin ZJ. A case-control study on the environmental risk factors of Parkinson's disease in Tianjin, China. Neuroepidemiology 1993;12(4):209–18.
84. Morano A, Jiménez-Jiménez FJ, Molina JA, Antolín MA. Risk-factors for Parkinson's disease: case-control study in the province of Cáceres, Spain. Acta Neurol Scand 1994;89(3):164–70.
85. Bonifati V, Fabrizio E, Vanacore N, De Mari M, Meco G. Familial Parkinson's disease: a clinical genetic analysis. Can J Neurol Sci 1995;22(4):272–9.
86. Vieregge P, Heberlein I. Increased risk of Parkinson's disease in relatives of patients. Ann Neurol 1995;37(5):685.
87. de Michele G, Filla A, Volpe G, de Marco V, Gogliettino A, Ambrosio G, et al. Environmental and genetic risk factors in Parkinson's disease: a case-control study in southern Italy. Mov Disord 1996;11(1):17–23.
88. Seidler A, Hellenbrand W, Robra BP, Vieregge P, Nischan P, Joerg J, et al. Possible environmental, occupational, and other etiologic factors for Parkinson's disease: a case-control study in Germany. Neurology 1996;46(5):1275–84.
89. Chan DK, Woo J, Ho SC, Pang CP, Law LK, Ng PW, et al. Genetic and environmental risk factors for Parkinson's disease in a Chinese population. J Neurol Neurosurg Psychiatry 1998;65(5):781–4.
90. Werneck AL, Alvarenga H. Genetics, drugs and environmental factors in Parkinson's disease. A case-control study. Arq Neuropsiquiatr 1999;57(2B):347–55.
91. Behari M, Srivastava AK, Das RR, Pandey RM. Risk factors of Parkinson's disease in Indian patients. J Neurol Sci 2001;190:49–55.
92. Galanaud JP, Elbaz A, Clavel J, Vidal JSB, Correze JR, Alperovitch A, et al. Cigarette smoking and Parkinson's disease: A case-control study in a population characterized by a high prevalence of pesticide exposure. Mov Disord 2005;20(2):181–9.
93. Sanyal J, Chakraborty DP, Sarkar B, Banerjee TK, Mukherjee SC, Ray BC, et al. Environmental and familial risk factors of Parkinsons disease: case-control study. Can J Neurol Sci 2010;37(5):637–42.
94. Lang AE, Kierans C, Blair RD. Family history of tremor in Parkinson's disease compared with those of controls and patients with idiopathic dystonia. Adv Neurol 1987;45:313–6.
95. Jankovic J, Beach J, Schwartz K, Contant C. Tremor and longevity in relatives of patients with Parkinson's disease, essential tremor, and control subjects. Neurology 1995;45(4):645–8.
96. Nefzger MD, Quadfasel FA, Karl VC. A retrospective study of smoking in Parkinson's disease. Am J Epidemiol 1968;88(2):149–58.
97. Kessler II. Epidemiologic studies of Parkinson's disease. 3. A community-based survey. Am J Epidemiol 1972;96(4):242–54.
98. Kessler II, Diamond EL. Epidemiologic studies of Parkinson's disease. I. Smoking and Parkinson's disease: a survey and explanatory hypothesis. Am J Epidemiol 1971;94(1):16–25.
99. Baumann RJ, Jameson HD, McKean HE, Haack DG, Weisberg LM. Cigarette smoking and Parkinson disease: 1. Comparison of cases with matched neighbors. Neurology 1980;30(8):839–43.
100. Marttila RJ, Rinne UK. Smoking and Parkinson's disease. Acta Neurol Scand 1980;62(5):322–5.
101. Godwin-Austen RB, Lee PN, Marmot MG, Stern GM. Smoking and Parkinson's disease. J Neurol Neurosurg Psychiatry 1982;45(7):577–81.
102. Rajput AH, Offord KP, Beard CM, Kurland LT. A case-control study of smoking habits, dementia, and other illnesses in idiopathic Parkinson's disease. Neurology 1987;37(2):226–32.
103. Tanner CM, Chen B, Wang WZ, Peng ML, Liu ZL, Liang XL, et al. Environmental factors in the etiology of Parkinson's disease. Can J Neurol Sci 1987;14(Suppl 3):419–23.
104. Ho SC, Woo J, Lee CM. Epidemiologic study of Parkinson's disease in Hong Kong. Neurology 1989;39(10):1314–8.
105. Hofman A, Collette HJ, Bartelds AI. Incidence and risk factors of Parkinson's disease in The Netherlands. Neuroepidemiology 1989;8(6):296–9.
106. Ngim CH, Devathasan G. Epidemiologic study on the association between body burden mercury level and idiopathic Parkinson's disease. Neuroepidemiology 1989;8(3):128–41.
107. Hertzman C, Wiens M, Bowering D, Snow B, Calne D. Parkinson's disease: a case-control study of occupational and environmental risk factors. Am J Ind Med 1990;17(3):349–55.
108. Sasco AJ, Paffenbarger RS. Smoking and Parkinson's disease. Epidemiology 1990;1(6):460–5.
109. Wechsler LS, Checkoway H, Franklin GM, Costa LG. A pilot study of occupational and environmental risk factors for Parkinson's disease. NeuroToxicology 1991;12(3):387–92.
110. Busenbark KL, Huber SJ, Greer G, Pahwa R, Koller WC. Olfactory function in essential tremor. Neurology 1992;42(8):1631–2.
111. Jiménez-Jiménez FJ, Mateo D, Giménez-Roldán S. Premorbid smoking, alcohol consumption, and coffee drinking habits in Parkinson's disease: a case-control study. Mov Disord 1992;7(4):339–44.
112. Mayeux R, Tang MX, Marder K, Côté LJ, Stern Y. Smoking and Parkinson's disease. Mov Disord 1994;9(2):207–12.
113. Martyn CN, Osmond C. Parkinson's disease and the environment in early life. J Neurol Sci 1995;132(2):201–6.
114. Hellenbrand W, Seidler A, Robra BP, Vieregge P, Oertel WH, Joerg J, et al. Smoking and Parkinson's disease: a case-control study in Germany. Int J Epidemiol 1997;26(2):328–39.
115. Liou HH, Tsai MC, Chen CJ, Jeng JS, Chang YC, Chen SY, et al. Environmental risk factors and Parkinson's disease: a case-control study in Taiwan. Neurology 1997;48(6):1583–8.
116. Tzourio C, Rocca WA, Breteler MM, Baldereschi M, Dartigues JF, Lopez-Pousa S, et al. Smoking and Parkinson's disease. An age-dependent risk effect? The EUROPARKINSON Study Group. Neurology 1997;49(5):1267–72.
117. de Palma G, Mozzoni P, Mutti A, Calzetti S, Negrotti A. Case-control study of interactions between genetic and environmental factors in Parkinson's disease. Lancet 1998;352(9145):1986–7.
118. Smargiassi A, Mutti A, De Rosa A, de Palma G, Negrotti A, Calzetti S. A case-control study of occupational and environmental risk factors for Parkinson's disease in the Emilia-Romagna region of Italy. NeuroToxicology 1998;19:709–12.
119. Gorell JM, Rybicki BA, Johnson CC, Peterson EL. Smoking and Parkinson's disease: a dose-response relationship. Neurology 1999;52(1):115–9.
120. Kuopio AM, Marttila RJ, Helenius H, Rinne UK. Environmental risk factors in Parkinson's disease. Mov Disord 1999;14(6):928–39.
121. Vanacore N, Bonifati V, Fabbrini G, Colosimo C, Marconi R, Nicholl D, et al. Smoking habits in multiple system atrophy and progressive supranuclear palsy. European Study Group on Atypical Parkinsonisms. Neurology 2000;54(1):114–9.
122. Paganini-Hill A. Risk factors for parkinson's disease: the leisure world cohort study. Neuroepidemiology 2001;20(2):118–124.
123. Baldereschi M, Di Carlo A, Vanni P, Ghetti A, Carbonin P, Amaducci L, et al. Lifestyle-related risk factors for Parkinson's disease: a population-based study. Acta Neurol Scand 2003;108(4):239–44.
124. Baldi I, Cantagrel A, Lebailly P, Tison FCO, Dubroca B, Chrysostome V, et al. Association between Parkinson's disease and exposure to pesticides in Southwestern France. Neuroepidemiology 2003;22(5):305–10.
125. Dong JQ, Zhang ZX, Zhang KL. Parkinson's disease and smoking: an integral part of PD's etiological study. Biomed Environ Sci 2003;16(2):173–9.
126. Pals P, van Everbroeck B, Grubben B, Viaene MK, Dom R, van der Linden C, et al. Case-control study of environmental risk factors for Parkinson's disease in Belgium. Eur J Epidemiol 2003;18(12):1133–42.
127. Tan E. Dose-dependent protective effect of coffee, tea, and smoking in Parkinson's disease: a study in ethnic Chinese. J Neurol Sci 2003;216(1):163–7.
128. Ma L, Zhang L, Gao XH, Chen W, Wu YP, Wang Y, et al. Dietary factors and smoking as risk factors for PD in a rural population in China: a nested case-control study. Acta Neurol Scand 2006;113(4):278–81.
129. Powers K, Smithweller T, Franklin G, Longstreth W, Swanson P, Checkoway H. Diabetes, smoking, and other medical conditions in relation to Parkinson's disease risk. Parkinsonism Relat Disord 2006;12(3):185–9.
130. Frigerio R, Breteler MMB, de Lau LML, Sanft KR, Bower JH, Ahlskog JE, et al. Number of children and risk of Parkinson's disease. Mov Disord 2007;22(5):632–9.
131. Kamel F, Tanner C, Umbach D, Hoppin J, Alavanja M, Blair A, et al. Pesticide exposure and self-reported Parkinson's disease in the Agricultural Health Study. Am J Epidemiol 2007;165(4):364–74.
132. Facheris MF, Schneider NK, Lesnick TG, de Andrade M, Cunningham JM, Rocca WA, et al. Coffee, caffeine-related genes, and Parkinson's disease: A case-control study. Mov Disord 2008;23(14):2033–40.
133. Petersen M, Halling J, Bech S, Wermuth L, Weihe P, Nielsen F, et al. Impact of dietary exposure to food contaminants on the risk of Parkinson's disease. NeuroToxicology 2008;29(4):584–90.
134. Powers KM, Kay DM, Factor SA, Zabetian CP, Higgins DS, Samii A, et al. Combined effects of smoking, coffee, and NSAIDs on Parkinson's disease risk. Mov Disord 2008;23(1):88–95.
135. D'Amelio MD, Ragonese P, Callari G, Di Benedetto N, Palmeri B, Terruso V, et al. Diabetes preceding Parkinson's disease onset. A case-control study. Parkinsonism Relat Disord 2009;15(9):660–4.
136. Gatto NM, Cockburn M, Bronstein J, Manthripragada AD, Ritz B. Well-water consumption and Parkinson's disease in rural California. Environ Health Perspect 2009;117(12):1912–8.
137. Tanner CM, Ross GW, Jewell SA, Hauser RA, Jankovic J, Factor SA, et al. Occupation and risk of parkinsonism: a multicenter case-control study. Arch Neurol 2009;66(9):1106–13.
138. Fang F, Xu Q, Park Y, Huang X, Hollenbeck A, Blair A, et al. Depression and the subsequent risk of Parkinson's disease in the NIH-AARP Diet and Health Study. Mov Disord 2010;25(9):1157–62.
139. Tanaka K, Miyake Y, Fukushima W, Sasaki S, Kiyohara C, Tsuboi Y, et al. Active and passive smoking and risk of Parkinson’s disease. Acta Neurol Scand 2010;122(6):377–82.
140. Chen H, Mosley TH, Alonso A, Huang X. Plasma urate and Parkinson's disease in the Atherosclerosis Risk in Communities (ARIC) study. Am J Epidemiol 2009;169(9):1064–9.
141. Checkoway H, Powers K, Smith-Weller T, Franklin GM, Longstreth WT, Swanson PD. Parkinson's disease risks associated with cigarette smoking, alcohol consumption, and caffeine intake. Am J Epidemiol 2002;155(8):732–8.
142. Sääksjärvi K, Knekt P, Rissanen H, Laaksonen MA, Reunanen A, Männistö S. Prospective study of coffee consumption and risk of Parkinson's disease. Eur J Clin Nutr 2008;62(7):908–15.
143. Hernán MA, Zhang SM, Rueda-DeCastro AM, Colditz GA, Speizer FE, Ascherio A. Cigarette smoking and the incidence of Parkinson's disease in two prospective studies. Ann Neurol 2001;50(6):780–6.
144. Thacker EL, O'Reilly EJ, Weisskopf MG, Chen H, Schwarzschild MA, McCullough ML, et al. Temporal relationship between cigarette smoking and risk of Parkinson disease. Neurology 2007;68(10):764–8.
145. Tan LC, Koh WP, Yuan JM, Wang R, Au WL, Tan JH, et al. Differential effects of black versus green tea on risk of Parkinson's disease in the Singapore Chinese Health Study. Am J Epidemiol 2008;167(5):553–60.
146. Chen H, Huang X, Guo X, Mailman RB, Park Y, Kamel F, et al. Smoking duration, intensity, and risk of Parkinson disease. Neurology 2010;74(11):878–84.
147. Nuti A, Ceravolo R, Dell'Agnello G, Gambaccini G, Bellini G, Kiferle L, et al. Environmental factors and Parkinson's disease: a case–control study in the Tuscany region of Italy. Parkinsonism Relat Disord 2004;10(8):481–5.
148. Ross GW. Association of coffee and caffeine intake with the risk of Parkinson disease. JAMA 2000;283(20):2674–9.
149. Ascherio A, Zhang SM, Hernan MA, Kawachi I, Colditz GA, Speizer FE, et al. Prospective study of caffeine consumption and risk of Parkinson's disease in men and women. Ann Neurol 2001;50(1):56–63.
150. Ascherio A. Coffee consumption, gender, and Parkinson's disease mortality in the Cancer Prevention Study II Cohort: the modifying effects of estrogen. Am J Epidemiol 2004;160(10):977–84.
151. Hu G, Bidel S, Jousilahti P, Antikainen R, Tuomilehto J. Coffee and tea consumption and the risk of Parkinson's disease. Mov Disord 2007;22(15):2242–8.
152. Hernán MA, Logroscino G, Garcia Rodriguez LA. A prospective study of alcoholism and the risk of Parkinson's disease. J Neurol 2004;251(S7):vii14–vii17.
153. Brighina L, Schneider NK, Lesnick TG, de Andrade M, Cunningham JM, Mrazek D, et al. Alpha-Synuclein, alcohol use disorders, and Parkinson disease: A case-control study. Parkinsonism Relat Disord 2009;15(6):430–4.
154. Hernán MA, Chen H, Schwarzschild MA, Ascherio A. Alcohol consumption and the incidence of Parkinson's disease. Ann Neurol 2003;54(2):170–5.
155. Hubble JP, Cao T, Hassanein RE, Neuberger JS, Koller WC. Risk factors for Parkinson's disease. Neurology;43(9):1693–7.
156. Shiba M, Bower JH, Maraganore DM, McDonnell SK, Peterson BJ, Ahlskog JE, et al. Anxiety disorders and depressive disorders preceding Parkinson's disease: a case-control study. Mov Disord 2000;15(4):669–77.
157. Leentjens AFG, van den Akker M, Metsemakers JFM, Lousberg R, Verhey FRJ. Higher incidence of depression preceding the onset of Parkinson's disease: a register study. Mov Disord 2003;18(4):414–8.
158. Alonso A, Rodriguez LAG, Logroscino G, Hernán MA. Use of antidepressants and the risk of Parkinson's disease: a prospective study. J Neurol Neurosurg Psychiatry 2009;80(6):671–4.
159. Weisskopf MG, Chen H, Schwarzschild MA, Kawachi I, Ascherio A. Prospective study of phobic anxiety and risk of Parkinson's disease. Mov Disord 2003;18(6):646–51.
160. Brandt-Christensen M, Kvist K, Nilsson FM, Andersen PK, Kessing LV. Treatment with antidepressants and lithium is associated with increased risk of treatment with antiparkinson drugs: a pharmacoepidemiological study. J Neurol Neurosurg Psychiatry 2006;77(6):781–3.
161. Becker C, Jick SS, Meier CR. Use of antihypertensives and the risk of Parkinson disease. Neurology 2008;70(16):1438–44.
162. Rugbjerg K, Friis S, Ritz B, Schernhammer ES, Korbo L, Olsen JH. Autoimmune disease and risk for Parkinson disease: a population-based case-control study. Neurology 2009;73(18):1462–8.
163. Miyake Y, Tanaka K, Fukushima W, Sasaki S, Kiyohara C, Tsuboi Y, et al. Case–control study of risk of Parkinson's disease in relation to hypertension, hypercholesterolemia, and diabetes in Japan. J Neurol Sci 2010;293:82–6.
164. Hu G, Jousilahti P, Bidel S, Antikainen R, Tuomilehto J. Type 2 diabetes and the risk of Parkinson's disease. Diabetes Care 2007;30(4):842–7.
165. Simon KC, Chen H, Schwarzschild M, Ascherio A. Hypertension, hypercholesterolemia, diabetes, and risk of Parkinson disease. Neurology 2007;69(17):1688–95.
166. Driver JA, Smith A, Buring JE, Gaziano JM, Kurth T, Logroscino G. Prospective cohort study of type 2 diabetes and the risk of Parkinson's disease. Diabetes Care 2008;31(10):2003–5.
167. Factor SA, Weiner WJ. Prior history of head trauma in Parkinson's disease. Mov Disord 1991;6(3):225–9.
168. Semchuk KM, Love EJ. Effects of agricultural work and other proxy-derived case-control data on Parkinson's disease risk estimates. Am J Epidemiol 1995;141(8):747–54.
169. Bower JH, Maraganore DM, Peterson BJ, McDonnell SK, Ahlskog JE, Rocca WA. Head trauma preceding PD: a case-control study. Neurology 2003;60(10):1610–5.
170. Rugbjerg K, Ritz B, Korbo L, Martinussen N, Olsen JH. Risk of Parkinson's disease after hospital contact for head injury: population based case-control study. BMJ 2008;337:a2494.
171. Golbe LI, Farrell TM, Davis PH. Follow-up study of early-life protective and risk factors in Parkinson's disease. Mov Disord 1990;5(1):66–70.
172. Koller W, Vetere-Overfield B, Gray C, Alexander C, Chin T, Dolezal J, et al. Environmental risk factors in Parkinson's disease. Neurology 1990;40(8):1218–21.
173. Hertzman C, Wiens M, Snow B, Kelly S, Calne D. A case-control study of Parkinson's disease in a horticultural region of British Columbia. Mov Disord 1994;9(1):69–75.
174. Chaturvedi S, Ostbye T, Stoessl AJ, Merskey H, Hachinski V. Environmental exposures in elderly Canadians with Parkinson's disease. Can J Neurol Sci 1995;22(3):232–4.
175. Fall PA, Fredrikson M, Axelson O, Granérus AK. Nutritional and occupational factors influencing the risk of Parkinson's disease: a case-control study in southeastern Sweden. Mov Disord 1999;14(1):28–37.
176. Gorell J, Petersen EL, Rybicki BA, Johnson CC. Multiple risk factors for Parkinson's disease. J Neurol Sci 2004;217(2):169–74.
177. Frigerio R, Sanft KR, Grossardt BR, Peterson BJ, Elbaz A, Bower JH, et al. Chemical exposures and Parkinson's disease: A population-based case–control study. Mov Disord 2006;21(10):1688–92.
178. Fong CS, Wu RM, Shieh JC, Chao YT, Fu YP, Kuao CL, et al. Pesticide exposure on southwestern Taiwanese with MnSOD and NQO1 polymorphisms is associated with increased risk of Parkinson's disease. Clinica Chimica Acta 2007;378:136–41.
179. Firestone JA, Lundin JI, Powers KM, Smith-Weller T, Franklin GM, Swanson PD, et al. Occupational factors and risk of Parkinson's disease: A population-based case-control study. Am J Ind Med 2010;53:217-23.
180. Hristina VD, Sipetic SB, Maksimovic JM, Marinkovic JM, Dzoljic ED, Ratkov IS, et al. Environmental factors and Parkinson's disease: A case-control study in Belgrade, Serbia. Int J Neurosci 2010;120(5):361–7.
181. Baldi I. Neurodegenerative diseases and exposure to pesticides in the elderly. Am J Epidemiol 2003;157(5):409–14.
182. Ascherio A, Chen H, Weisskopf MG, O'Reilly E, McCullough ML, Calle EE, et al. Pesticide exposure and risk for Parkinson's disease. Ann Neurol 2006;60(2):197–203.
183. Semchuk KM, Love EJ, Lee RG. Parkinson's disease and exposure to rural environmental factors: a population based case-control study. Can J Neurol Sci 1991;18(3):279–86.
184. Rocca WA, Anderson DW, Meneghini F, Grigoletto F, Morgante L, Reggio A, et al. Occupation, education, and Parkinson's disease: a case-control study in an Italian population. Mov Disord 1996;11(2):201–6.
185. Kirkey KL, Johnson CC, Rybicki BA, Peterson EL, Kortsha GX, Gorell JM. Occupational categories at risk for Parkinson's disease. Am J Ind Med 2001;39(6):564–71.
186. Frigerio R, Elbaz A, Sanft KR, Peterson BJ, Bower JH, Ahlskog JE, et al. Education and occupations preceding Parkinson disease: a population-based case-control study. Neurology 2005;65(10):1575–83.
187. Park J, Yoo C, Sim C, Kim H, Kim J, Jeon B, et al. Occupations and Parkinson's disease: A multi-center case-control study in South Korea. NeuroToxicology 2005;26(1):99–105.
188. Dick S, Semple S, Dick F, Seaton A. Occupational titles as risk factors for Parkinson's disease. Occupational Medicine 2007;57(1):50–6.
189. Tanner CM, Chen B, Wang W, Peng M, Liu Z, Liang X, et al. Environmental factors and Parkinson's disease: a case-control study in China. Neurology 1989;39(5):660–4.
190. Gorell JM, Johnson CC, Rybicki BA, Peterson EL, Richardson RJ. The risk of Parkinson's disease with exposure to pesticides, farming, well water, and rural living. Neurology 1998;50(5):1346–50.
191. Park J, Yoo CI, Sim CS, Kim JW, Yi Y, Jung KY, et al. Occupations and Parkinson's disease: a case-control study in South Korea. Ind Health 2004;42(3):352–8.
192. Firestone JA, Smith-Weller T, Franklin G, Swanson P, Longstreth WT, Checkoway H. Pesticides and risk of Parkinson disease: a population-based case-control study. Arch Neurol 2005;62(1):91–5.
193. O'Reilly EJ, Chen H, Gardener H, Gao X, Schwarzschild MA, Ascherio A. Smoking and Parkinson's disease: Using parental smoking as a proxy to explore causality. Am J of Epidemiol 2009;169(6):678–82.
194. O'Reilly EJ, McCullough ML, Chao A, Jane Henley S, Calle EE, Thun MJ, et al. Smokeless tobacco use and the risk of Parkinson's disease mortality. Mov Disord 2005;20(10):1383–4.
195. Hellenbrand W, Seidler A, Boeing H, Robra BP, Vieregge P, Nischan P, et al. Diet and Parkinson's disease. I: A possible role for the past intake of specific foods and food groups. Results from a self-administered food-frequency questionnaire in a case-control study. Neurology 1996;47(3):636–43.
196. Tan EK, Chua E, Fook-Chong SM, Teo YY, Yuen Y, Tan L, et al. Association between caffeine intake and risk of Parkinson's disease among fast and slow metabolizers. Pharmacogenet Genomics 2007;17(11):1001–5.
197. Chan D, Mellick GD, Hung WT, Woo J. Genetic and environmental risk factors and their interactions for Parkinson's disease in a Chinese population. J Clin Neurosci 2003;10(3):313–5.
198. Savica R, Carlin JM, Grossardt BR, Bower JH, Ahlskog JE, Maraganore DM, et al. Medical records documentation of constipation preceding Parkinson disease: A case-control study. Neurology 2009;73(21):1752–8.
199. Abbott RD, Petrovitch H, White LR, Masaki KH, Tanner CM, Curb JD, et al. Frequency of bowel movements and the future risk of Parkinson's disease. Neurology 2001 Aug;57(3):456–62.
200. Elbaz A, Peterson BJ, Yang P, van Gerpen JA, Bower JH, Maraganore DM, et al. Nonfatal cancer preceding Parkinson's disease: a case-control study. Epidemiology 2002;13(2):157–64.
201. D'Amelio M, Ragonese P, Morgante L, Epifanio A, Callari G, Salemi G, et al. Tumor diagnosis preceding Parkinson's disease: A case-control study. Mov Disord 2004;19(7):807–11.
202. Olsen JRH, Friis SR, Frederiksen K. Malignant melanoma and other types of cancer preceding Parkinson disease. Epidemiology 2006;17(5):582–7.
203. Driver JA, Kurth T, Buring JE, Gaziano JM, Logroscino G. Prospective case–control study of nonfatal cancer preceding the diagnosis of parkinson’s disease. Cancer Causes Control 2007;18(7):705–11.
204. Gao X, Simon KC, Han J, Schwarzschild MA, Ascherio A. Family history of melanoma and Parkinson disease risk. Neurology 2009;73(16):1286–91.
205. Gao X, Simon KC, Han J, Schwarzschild MA, Ascherio A. Genetic determinants of hair color and parkinson's disease risk. Ann Neurol 2009;65(1):76–82.
206. O'Reilly EJ, Gao X, Weisskopf MG, Chen H, Schwarzschild MA, Spiegelman D, et al. Plasma urate and Parkinson's disease in women. Am J Epidemiol 2010;172(6):666–70.
207. Weisskopf M, O'Reilly E, Chen H, Schwarzschild M, Ascherio A. Plasma urate and risk of Parkinson's disease. Am J Epidemiol 2007;166(5):561–7.
208. Davis JW, Grandinetti A, Waslien CI, Ross GW, White LR, Morens DM. Observations on serum uric acid levels and the risk of idiopathic Parkinson's disease. Am J Epidemiol 1996;144(5):480–4.
209. de Lau LML, Koudstaal PJ, Hofman A, Breteler MMB. Serum uric acid levels and the risk of Parkinson disease. Ann Neurol 2005;58(5):797–800.
210. Alonso A, Rodríguez LAG, Logroscino G, Hernán MA. Gout and risk of Parkinson disease: a prospective study. Neurology 2007;69(17):1696–1700.
211. Hu G, Antikainen R, Jousilahti P, Kivipelto M, Tuomilehto J. Total cholesterol and the risk of Parkinson disease. Neurology 2008;70(21):1972–9.
212. Huang X, Chen H, Miller WC, Mailman RB, Woodard JL, Chen PC, et al. Lower low-density lipoprotein cholesterol levels are associated with Parkinson's disease. Mov Disord 2007;22(3):377–81.
213. Huang X, Abbott RD, Petrovitch H, Mailman RB, Ross GW. Low LDL cholesterol and increased risk of Parkinson's disease: Prospective results from Honolulu-Asia Aging Study. Mov Disord 2008;23(7):1013–8.
214. Abbott RD, Ross GW, White LR, Nelson JS, Masaki KH, Tanner CM, et al. Midlife adiposity and the future risk of Parkinson's disease. Neurology 2002;59(7):1051–7.
215. Hu G, Jousilahti P, Nissinen A, Antikainen R, Kivipelto M, Tuomilehto J. Body mass index and the risk of Parkinson disease. Neurology 2006;67(11):1955–9.
216. Chen H, Zhang SM, Schwarzschild MA, Hernán MA, Willet WC, Ascherio A. Obesity and the risk of Parkinson's disease. Am J Epidemiol 2004;159(6):547–555.
217. Logroscino G, Sesso HD, Paffenbarger RS, Lee IM. Body mass index and risk of Parkinson's disease: A prospective cohort study. Am J Epidemiol 2007;166(10):1186–90.
218. Ragonese P, D'Amelio M, Callari G, Di Benedetto N, Palmeri B, Mazzola MA, et al. Body mass index does not change before Parkinson’s disease onset. Eur J Neurol 2008;15(9):965–8.
219. Xu Q, Park Y, Huang X, Hollenbeck A, Blair A, Schatzkin A, et al. Physical activities and future risk of Parkinson disease. Neurology 2010;75(4):341–8.
220. Sasco AJ, Paffenbarger RS, Gendre I, Wing AL. The role of physical exercise in the occurrence of Parkinson's disease. Arch Neurol 1992;49(4):360–5.
221. de Lau LML, Koudstaal PJ, Hofman A, Breteler MMB. Serum cholesterol levels and the risk of Parkinson's disease. Am J Epidemiol 2006;164:998–1002
222. Sasco AJ, Paffenbarger RS. Measles infection and Parkinson's disease. Am J Epidemiol 1985;122(6):1017–31.
223. Bower JH, Maraganore DM, Peterson BJ, Ahlskog JE, Rocca WA. Immunologic diseases, anti-inflammatory drugs, and Parkinson disease: A case-control study. Neurology 2006;67(3):494–6.
224. Savica R, Grossardt BR, Carlin JM, Icen M, Bower JH, Ahlskog JE, et al. Anemia or low hemoglobin levels preceding Parkinson disease: a case-control study. Neurology 2009;73(17):1381–7.
225. Wahner AD, Bronstein JM, Bordelon YM, Ritz B. Nonsteroidal anti-inflammatory drugs may protect against Parkinson disease. Neurology 2007;69(19):1836–42.
226. Ton TG, Heckbert SR, Longstreth WT, Rossing MA, Kukull WA, Franklin GM, et al. Nonsteroidal anti-inflammatory drugs and risk of Parkinson's disease. Mov Disord 2006;21(7):964–9.
227. Hernán MA, Logroscino G, García Rodríguez LA. Nonsteroidal anti-inflammatory drugs and the incidence of Parkinson disease. Neurology 2006;66(7):1097–9.
228. Chen H, Zhang SM, Hernán MA, Schwarzschild MA, Willett WC, Colditz GA, et al. Nonsteroidal anti-inflammatory drugs and the risk of Parkinson disease. Arch Neurol 2003;60(8):1059–64.
229. Chen H, Jacobs E, Schwarzschild MA, McCullough ML, Calle EE, Thun MJ, et al. Nonsteroidal antiinflammatory drug use and the risk for Parkinson's disease. Ann Neurol 2005;58(6):963–7.
230. Bornebroek M, de Lau LML, Haag MDM, Koudstaal PJ, Hofman A, Stricker BHC, et al. Nonsteroidal anti-inflammatory drugs and the risk of Parkinson disease. Neuroepidemiology 2007;28(4):193–6.
231. Etminan M, Carleton B, Samii A. Non-steroidal anti-inflammatory drug use and the risk of Parkinson disease: A retrospective cohort study. J Clin Neurosci 2008;15(5):576–7.
232. Samii A, Carleton BC, Etminan M. Statin use and the risk of Parkinson disease: a nested case control study. J Clin Neurosci 2008;15(11):1272–3.
233. Ritz B, Manthripragada AD, Qian L, Schernhammer E, Wermuth L, Olsen J, et al. Statin use and Parkinson's disease in Denmark. Mov Disord 2010;25(9):1210–6.
234. Wahner AD, Bronstein JM, Bordelon YM, Ritz B. Statin use and the risk of Parkinson disease. Neurology 2008;70(16):1418–22.
235. Marder K, Tang MX, Alfaro B, Mejia H, Cote L, Jacobs D, et al. Postmenopausal estrogen use and Parkinson's disease with and without dementia. Neurology 1998;50(4):1141–3.
236. Benedetti MD, Maraganore DM, Bower JH, McDonnell SK, Peterson BJ, Ahlskog JE, et al. Hysterectomy, menopause, and estrogen use preceding Parkinson's disease: An exploratory case-control study. Mov Disord 2001;16(5):830–7.
237. Martignoni E, Nappi RE, Citterio A, Calandrella D, Zangaglia R, Mancini F, et al. Reproductive life milestones in women with Parkinson's disease. Funct Neurol 2003;18(4):211–7.
238. Currie LJ, Harrison MB, Trugman JM, Bennett JP, Wooten GF. Postmenopausal estrogen use affects risk for Parkinson disease. Arch Neurol 2004;61(6):886–8.
239. Ragonese P, D'Amelio M, Salemi G, Aridon P, Gammino M, Epifanio A, et al. Risk of Parkinson disease in women: effect of reproductive characteristics. Neurology 2004;62(11):2010–4.
240. Popat RA, van den Eeden SK, Tanner CM, McGuire V, Bernstein AL, Bloch DA, et al. Effect of reproductive factors and postmenopausal hormone use on the risk of Parkinson disease. Neurology 2005;65(3):383–90.
241. Simon KC, Chen H, Gao X, Schwarzschild MA, Ascherio A. Reproductive factors, exogenous estrogen use, and risk of Parkinson's disease. Mov Disord 2009;24(9):1359–65.
242. Ton TGN, Heckbert SR, Longstreth WT, Rossing MA, Kukull WA, Franklin GM, et al. Calcium channel blockers and beta-blockers in relation to Parkinson's disease. Parkinsonism Relat Disord 2007;13(3):165–9.
243. Louis ED, Benito-Leon J, Bermejo-Pareja F. Antihypertensive agents and risk of Parkinson's disease, essential tremor and dementia: A population-based prospective study (NEDICES). Neuroepidemiology 2009;33(3):286–92.
244. Ritz B, Rhodes SL, Qian L, Schernhammer E, Olsen J, Friis S. L-type calcium channel blockers and Parkinson's disease in Denmark. Ann Neurol 2010;25(9):600-6.
245. Simon KC, Gao X, Chen H, Schwarzschild MA, Ascherio A. Calcium channel blocker use and risk of Parkinson's disease. Mov Disord 2010;25(12):1818–22.
246. Benedetti MD, Bower JH, Maraganore DM, McDonnell SK, Peterson BJ, Ahlskog JE, et al. Smoking, alcohol, and coffee consumption preceding Parkinson's disease: a case-control study. Neurology 2000;55:1350–8.
247. Fukishima W, Miyake Y, Tanaka K, Sasaki S, Kiyohara C, Tsuboi Y, et al. Alcohol drinking and risk of Parkinson's disease: a case-control study in Japan. BMC Neurology 2010;10:111.
248. Tan EK, Chai A, Lum SY, Shen H, Tan C, Teoh ML, et al. Monoamine oxidase B polymorphism, cigarette smoking and risk of Parkinson's disease: a study in an Asian population. Am J Med Genetics 2003;120B:58-62
249. Skeie GO, Muller B, Haugarvoll K, Larsen JP, Tysnes OB. Differential effect of environmental risk factors on postural instability gait difficulties and tremor dominant Parkinson's disease. Mov Disord 2010;25(12):1847-52
