# Supplementary material for: Meta-Analysis of Early Nonmotor Features and Risk Factors for Parkinson Disease
Source: Ann Neurol. 2012 Oct 15;72(6):893–901. doi: 10.1002/ana.23687 (PMC3556649; doi:10.1002/ana.23687)
Supplement: Supplementary file 2 [file ana0072-0893-SD2.doc]

|  | **Table 1 - Details of Studies included in the meta-analysis** | | | | | |  |  |  |  |  |  |  |  |  |  |
| --- | --- | --- | --- | --- | --- | --- | --- | --- | --- | --- | --- | --- | --- | --- | --- | --- |
|  |  |  |  |  |  |  |  |  |  |  |  |  |  |  |  |  |
|  | Case control studies - First degree relative with PD | | | | | |  |  |  |  |  |  |  |  |  |  |
|  |  |  |  |  |  |  |  |  |  |  |  |  |  |  |  |  |
| **Ref** | **Year** | **First Author** | **Country** | **Study period** | **Resource** | **Cases** | **Male** | **Female** | **Mean Age** | **Controls** | **Male** | **Female** | **Mean Age** | **Definition** | **Exposure assessment** | **Adjusted for** |
| 70 | 1973 | Martin | US | - | University of Minnesota Hospital | 130 | 80 | 50 | - | 115 | 43 | 72 | - | Neurologist diagnosed PD (2 of 4 cardinal signs) | Structured questionnaire | None |
| 57 | 1986 | Alonso | Mexico | 1986 | INNN, Mexico City | 105 | - | - | - | 100 | 50 | 50 | - | Neurologist diagnosed PD (2 of 4 cardinal signs) | Structured interview | None |
| 78 | 1993 | Semchuk | Canada | 1984-1987 | Calgary Residents Longterm Care Registry | 130 | 75 | 55 | 68.5 | 260 | 150 | 110 | 68.3 | Neurologist diagnosed PD | Structured interview | Head trauma, herbicide use |
| 68 | 1996 | Marder | US | - | PD Registry, Residents of Northern Manhatten, New York | 233 | 123 | 110 | 73.6 | 1172 | 312 | 860 | 75.6 | Formal diagnostic criteria | Structured interview | Sex, ethnicity and relation to proband |
| 72 | 1997 | Mickel | US | - | Marshfield Neurology Clinic | 101 | 70 | 31 | 67.8 | 101 | 31 | 70 | 65.6 | Neurologist-diagnosed PD + medical record review | Structured interview | None |
| 71 | 1998 | McCann | Australia | - | Clinics and Residential Care Centres, Queensland and New South Wales | 224 | 131 | 93 | 70.3 | 310 | 193 | 117 | 68.9 | PD diagnosed according to Calne criteria | Structured questionnaire | None |
| 77 | 1999 | Rybicki | US | 1991-1995 | Henry Ford Health System Cohort, Detroit | 144 | - | - | - | 464 | - | - | - | Neurologist diagnosed PD | Structured interview | None |
| 81 | 1999 | Taylor | US | - | Movement Disorder Centre at Boston Medical Centre | 140 | 88 | 52 | 66.2 | 147 | 90 | 57 | 66.9 | Neurologist diagnosed PD (Ward and Gibb criteria) | Structured interview | None |
| 58 | 2000 | Autere | Finland | 1996 | Oulu University Hospital | 268 | 133 | 135 | - | 210 | 103 | 107 | - | Medical record review PD (Brain Bank criteria) | Structured interview | None |
| 61 | 2000 | Elbaz | Europe | - | EUROPARKINSON study | 127 | 57 | 70 | 78 | 306 | 137 | 169 | 78 | Screened for Parkinsonism, then examined by neurologist to confirm PD | Structured interview | Age, sex, centre, sibship size, education level |
| 67 | 2000 | la Fuente-Fernandez | Spain | 1999 | Neurology Clinic Hospital A. Marcide | 299 | 145 | 154 | 70.5 | 295 | 134 | 161 | 68.1 | Diagnosed definite PD and levodopa responsive | Structured interview | Age, sex |
| 74 | 2000 | Preux | France | 1995-1996 | Limoges University Hospital, France | 140 | - | - | - | 280 | - | - | - | Physician examined (UK Brain Bank Criteria) | Structured interview | Smoking, coffee, tea, urban area, toxic products |
| 62 | 2001 | Herishanu | Israel | 1989-1995 | PD clinic of Soroka University Medical Centre | 93 | - | - | - | 93 | - | - | - | Progressive disorder, 2 or more cardinal signs of PD | Structured questionnaire | None |
| 65 | 2001 | Kuopio | Finland | 1992-1995 | Turku city and 9 rural municipalities | 119 | 61 | 58 | 68.7 | 238 | 122 | 116 | 69.2 | Neurologist examined (UK Brain Bank Criteria) | Structured interview | None |
| 73 | 2002 | Payami | US | - | Movement Disorder Centre at Oregon Health Sciences University | 460 | 277 | 183 | - | 114 | 63 | 51 | - | Formal diagnostic criteria | Structured questionnaire | Age |
| 82 | 2002 | Zorzon | Italy | 1998 | Center for Parkinson's disease and Movement Disorders, Trieste | 136 | 62 | 74 | 70 | 272 | 124 | 148 | 69 | Neurologist diagnosed PD (2 of 4 cardinal signs) | Structured interview | Smoking |
| 60 | 2003 | Duzcan | Turkey | 2000 | Kizilcaboluk-Denizli | 36 | 17 | 19 | - | 108 | 51 | 57 | - | Neurologist diagnosed Parkinsonism (2 of 4 cardinal signs) + levodopa response | Structured questionnaire | None |
| 66 | 2003 | Kurz | Norway | 1992-1993 | 9 Municipalities in Rogaland, Western Norway | 245 | 120 | 125 | 73.5 | 100 | 49 | 51 | 72.8 | Formal diagnostic criteria | Structured questionnaire | None |
| 69 | 2003 | Marder | US | - | Centre for PD, Columbia Presbyterian Medical Centre | 266 | 152 | 114 | 70.6 | 409 | 219 | 190 | 66.6 | Medical record review PD (2 of 4 cardinal signs) | Structured interview | Sex, years of education, ethnicity |
| 64 | 2004 | Kourchounov | Germany/Russia | 1994-2001 | Clinic for Nervous Diseases, Setchenov Academy for Medicine, Moscow or Schlossberg Clinic for Parkinson’s Disease and Multiple Sclerosis, Bad Laasphe, Germany | 366 | 199 | 157 | 59.1 | 412 | 227 | 185 | - | UK Brain Bank Criteria | Structured interview | None |
| 75 | 2004 | Rocca | US | 1976-1995 | Rochester Epidemiology Project, Olmsted County, Minnesota | 162 | - | - | - | 147 | - | - | - | Medical record review for PD diagnosis | Structured interview | None |
| 80 | 2004 | Spanaki | Greece | 1997-2002 | Neurology Services, University Hospital of Crete | 247 | - | - | - | 247 | - | - | - | Physician examined (2 of 3 cardinal signs) | Structured interview | None |
| 59 | 2007 | Dick | UK | 2000-2004 | GEOPARKINSON study | 767 | 426 | 341 | - | 1989 | 1057 | 932 | - | Physician confirmed or medical record review PD (Brain Bank criteria) | Structured interview | Age, sex, country, tobacco use, ever knocked unconscious |
| 76 | 2007 | Rosen | US | 1992-2005 | Research registry in Emory University's Department of Neurology | 585 | 368 | 217 | 68 | 228 | 94 | 141 | 76.4 | Neurologist diagnosed PD (2 of 4 cardinal signs) | Structured interview | Age, race and gender |
| 79 | 2010 | Shino | US | 1994-1995 | Kaiser Permanente Medical Care Programme, Northern California | 400 | 240 | 160 | 70.1 | 448 | 270 | 178 | 70.4 | Medical record review PD (2 of 4 cardinal signs) | Structured interview | Sex, white non-Hispanic and relationship |
| 63 | 2010 | Jacob | US | - | Parkinson's Environment and Genes Study | 371 | 208 | 163 | 68.1 | 402 | 202 | 200 | 65.9 | Neurologist diagnosed PD (2 of 4 cardinal signs and absence of atypical features) | Structured interview | None |
|  |  |  |  |  |  |  |  |  |  |  |  |  |  |  |  |  |
|  |  |  |  |  |  |  |  |  |  |  |  |  |  |  |  |  |
|  | Case control studies - Any family history of PD | | | | | |  |  |  |  |  |  |  |  |  |  |
|  |  |  |  |  |  |  |  |  |  |  |  |  |  |  |  |  |
| **Ref** | **Year** | **First Author** | **Country** | **Study period** | **Resource** | **Cases** | **Male** | **Female** | **Mean Age** | **Controls** | **Male** | **Female** | **Mean Age** | **Definition** | **Exposure assessment** | **Matching for** |
| 78 | 1993 | Semchuk | Canada | 1989 | Calgary Residents | 130 | 75 | 55 | 68.5 | 260 | 150 | 110 | 68.3 | Neurologist diagnosed PD | Structured interview | None |
| 83 | 1993 | Wang | China | - | Department of Neurology, Tianjin General Hospital | 93 | 63 | 30 | 61 | 186 | 126 | 60 | 60 | Neurologist diagnosed PD (2 or more cardinal signs) | Structured interview | None |
| 84 | 1994 | Morano | Spain | 1989-1990 | General Hospitals in Caceres, Spain | 74 | 33 | 41 | 68.2 | 148 | 66 | 82 | 67.5 | Diagnostic criteria for PD | Structured questionnaire | None |
| 85 | 1995 | Bonifati | Italy | 1993 | Neurology Clinic Rome | 100 | 61 | 39 | 68 | 100 | 39 | 61 | 66 | 2 of 3 cardinal signs of PD | Structured interview | None |
| 86 | 1995 | Vieregge | Germany | - | Lubeck | 66 | - | - | - | 72 | - | - | - | - | Interview | None |
| 87 | 1996 | de Michele | Italy | - | Department of Neurology Federico II University in Naples | 116 | 77 | 39 | 62.5 | 232 | 116 | 116 | 62.4 | 2 of 3 cardinal signs of PD | Structured questionnaire | None |
| 88 | 1996 | Seidler | Germany | - | Nine Neurology clinics across Germany | 380 | 251 | 129 | 56.2 | 359 | - | - | 56.5 | Neurologist diagnosed (UK Brain Bank Criteria) | Structured interview | Smoking, education |
| 89 | 1998 | Chan | Hong Kong | - | 2 Hospitals in Hong Kong | 215 | 114 | 101 | - | 313 | 171 | 142 | - | Neurologist diagnosed PD (Maranganore criteria) | Structured interview | Smoking, pesticides, tea, rural living, well water, dietary factors, farming |
| 71 | 1998 | McCann | Australia | - | Clinics and Residential Care Centres, Queensland and New South Wales | 224 | 131 | 93 | 70.3 | 310 | 193 | 117 | 68.9 | Calne criteria | Questionnaire | Age, sex, rural residency, well water, HTN, stroke |
| 77 | 1999 | Rybicki | US | 1991-1995 | Henry Ford Health System Cohort, Detroit | 144 | 89 | 55 | 70 | 464 | - | - |  | Neurologist diagnosed PD | Interview with structured questionnaire | None |
| 90 | 1999 | Werneck | Brazil | 1996-1997 | Neurology Department of IASERJ Central Hospital | 92 | 41 | 51 | 70.6 | 110 | 47 | 63 | 68.4 | Neurologist diagnosed PD (Calne criteria) | Structured questionnaire | None |
| 91 | 2001 | Behari | India | 1994-1998 | Movement Disorder clinic of AIIMS, New Dehli | 377 | 301 | 76 | 56.8 | 377 | 271 | 106 | 56.6 | Neurologist diagnosed PD (2 of 3 cardinal signs) | Structured questionnaire | Age |
| 65 | 2001 | Kuopio | Finland | 1992-1995 | Turku city and 9 rural municipalities | 119 | 61 | 58 | 68.7 | 238 | 122 | 116 | 69.2 | Neurologist examined (UK Brain Bank Criteria) | Interview | None |
| 60 | 2003 | Duzcan | Turkey | 2000 | Kizilcaboluk-Denizli | 36 | 17 | 19 | - | 108 | 51 | 57 | - | Neurologist diagnosed Parkinsonism (2 of 4 cardinal signs) + levodopa response | Questionnaire | None |
| 66 | 2003 | Kurz | Norway | 1992-1993 | 9 Municipalities in Rogaland, Western Norway | 245 | 120 | 125 | 73.5 | 100 | 49 | 51 | 72.8 | Formal diagnostic criteria | Questionnaire | None |
| 64 | 2004 | Kourchounov | Germany/ Russia | 1994-2001 | Clinic for Nervous Diseases, Setchenov Academy for Medicine, Moscow or Schlossberg Clinic for Parkinson’s Disease and Multiple Sclerosis, Bad Laasphe, Germany | 366 | 199 | 157 | 59.1 | 412 | 227 | 185 | - | UK Brain Bank Criteria | Living relatives contacted | None |
| 92 | 2005 | Galanaud | France | 1998-1999 | Mutualite Sociale Agricole, French health insurance system | 247 | 138 | 109 | 69 | 676 | 377 | 299 | 69 | Neurologist diagnosed PD (2 or more cardinal signs) | Structured interview | Age, sex, education, alcohol, farming, pesticides, smoking |
| 76 | 2007 | Rosen | US | 1992-2005 | Research registry in Emory University's Department of Neurology | 585 | 368 | 217 | 68 | 228 | 94 | 141 | 76.4 | Neurologist diagnosed PD (2 or more cardinal signs) | Structured interview | Age, race, gender |
| 93 | 2010 | Sanyal | India | - | Movement Disorders clinic in Kolkata | 175 | 140 | 35 | 55.2 | 350 | 280 | 70 | 55 | 3 of 4 cardinal signs of PD | Structured interview | Pesticides, rural living, depression, toxins, smoking |
|  |  |  |  |  |  |  |  |  |  |  |  |  |  |  |  |  |
|  |  |  |  |  |  |  |  |  |  |  |  |  |  |  |  |  |
|  | Case control studies - Any family history of tremor | | | | | |  |  |  |  |  |  |  |  |  |  |
|  |  |  |  |  |  |  |  |  |  |  |  |  |  |  |  |  |
| **Ref** | **Year** | **First Author** | **Country** | **Study period** | **Resource** | **Cases** | **Male** | **Female** | **Mean Age** | **Controls** | **Male** | **Female** | **Mean Age** | **Definition** | **Exposure assessment** | **Matching for** |
| 94 | 1986 | Lang | Canada | - | Movement Disorders clinic, Toronto Western Hospital | 159 | 101 | 58 | 62.9 | 104 | 64 | 50 | 62.8 | Diagnosed PD | Interview | None |
| 78 | 1993 | Semchuk | Canada | 1989 | Calgary Residents | 130 | 75 | 55 | 68.5 | 260 | 150 | 110 | 68.3 | Neurologist diagnosed PD | Structured interview | None |
| 84 | 1994 | Morano | Spain | 1989-1990 | General Hospitals in Caceres, Spain | 74 | 33 | 41 | 68.2 | 148 | 66 | 82 | 67.5 | Diagnostic criteria for PD | Structured questionnaire | None |
| 95 | 1995 | Jankovic | US | 1980-1995 | Movement Disorders clinic, Baylor College of Medicine, Houston | 391 | 214 | 177 | 65 | 104 | 45 | 59 | 64 | Diagnosed PD | Interview | None |
| 86 | 1995 | Vieregge | Germany | - | Lubeck | 66 | - | - | - | 72 | - | - | - | Not specified | Interview | None |
| 87 | 1996 | de Michele | Italy | - | Department of Neurology Federico II University in Naples | 116 | 77 | 39 | 62.5 | 232 | 116 | 116 | 62.4 | 2 of 3 cardinal signs of PD | Structured questionnaire | None |
| 77 | 1999 | Rybicki | US | 1991-1995 | Henry Ford Health System Cohort, Detroit | 144 | 89 | 55 | 70 | 464 | - | - |  | Neurologist diagnosed PD | Interview with structured questionnaire | None |
| 81 | 1999 | Taylor | US | - | Movement Disorder Centre at Boston Medical Centre | 140 | 88 | 52 | 66.2 | 147 | 90 | 57 | 66.9 | Neurologist diagnosed PD (Ward and Gibb criteria) | Structured interview | Birth cohort, sex, head injury, FHx PD, depression, education, rural living, urban living, pesticides, well water, smoking, vitamins |
| 82 | 2002 | Zorzon | Italy | 1998 | Center for Parkinson's disease and Movement Disorders, Trieste | 136 | 62 | 74 | 70 | 272 | 124 | 148 | 69 | Neurologist diagnosed PD (2 of 4 cardinal signs) | Structured interview | Smoking, FHx of PD, maternal age, anaesthesia, farming, well water |
| 79 | 2010 | Shino | US | 1994-1995 | Kaiser Permanente Medical Care Programme, Northern California | 400 | 240 | 160 | 70.1 | 448 | 270 | 178 | 70.4 | Medical record review PD (2 of 4 cardinal signs) | Structured interview | Sex, white non-Hispanic and relationship |
|  |  |  |  |  |  |  |  |  |  |  |  |  |  |  |  |  |
|  |  |  |  |  |  |  |  |  |  |  |  |  |  |  |  |  |
|  | Case control studies - Ever smoking versus never | | | | | |  |  |  |  |  |  |  |  |  |  |
|  |  |  |  |  |  |  |  |  |  |  |  |  |  |  |  |  |
| **Ref** | **Year** | **First Author** | **Country** | **Study period** | **Resource** | **Cases** | **Male** | **Female** | **Mean Age** | **Controls** | **Male** | **Female** | **Mean Age** | **Definition** | **Exposure assessment** | **Matching for** |
| 96 | 1968 | Nefzger | US | - | Veterans Administration Central Office, Washington | 198 | 198 | - | - | 198 | 198 | - | - | Neurologist diagnosed PD | Structured interview | None |
| 98 | 1971 | Kessler | US | 1965-1969 | Baltimore hospital | 468 | 243 | 225 | - | 468 | 243 | 225 | - | Physician-diagnosed PD | Structured interview | None |
| 97 | 1972 | Kessler | US | 1967-1969 | Private physician referrals in Baltimore area | 228 | 122 | 106 | - | 228 | 122 | 106 | - | Physician-diagnosed PD | Structured interview | None |
| 99 | 1980 | Baumann | US | 1975-1978 | University and Veterans Administration hospitals | 237 | - | - | - | 474 | - | - | - | Neurologist diagnosed PD | Structured interview | None |
| 100 | 1980 | Marttila | Finland | 1972-1973 | Southwestern Finland | 443 | - | - | - | 443 | - | - | - | Not specified | Structured interview | None |
| 101 | 1982 | Godwin-Austen | UK | - | General Practices around the UK | 350 | 179 | 171 | - | 350 | 179 | 171 | - | Not specified | Questionnaire | None |
| 102 | 1987 | Rajput | US | 1967-1979 | Rochester Residents | 118 | 54 | 64 | - | 236 | 108 | 128 | - | Medical record review | Medical record review | None |
| 103 | 1987 | Tanner | China | - | Beijing and Guangzhou, Peoples Republic of China | 100 | 77 | 23 | 57.2 | 200 | 154 | 46 | 56.8 | Neurologist diagnosed | Structured interview | None |
| 104 | 1989 | Ho | Hong Kong | - | 10 Old Age Homes in Shatin and Tai Po | 35 | 11 | 24 | - | 105 | - | - | - | Trained medical students using diagnostic criteria | Structured interview | None |
| 105 | 1989 | Hofman | Holland | 1981-1986 | Sentinel Practices of the Netherlands Institute for General Practice | 86 | - | - | - | 172 | - | - | - | Neurologist diagnosed | Structured questionnaire | Age, gender |
| 106 | 1989 | Ngim | Singapore | 1985-1987 | Singapore General Hospital and National University Hospital | 54 | 36 | 18 | - | 95 | 60 | 35 | - | Neurologist diagnosed | Structured interview | None |
| 107 | 1990 | Hertzman | Canada | - | British Columbia | 57 | - | - | - | 122 | - | - | - | Neurologist diagnosed | Structured questionnaire | Age, sex |
| 108 | 1990 | Sasco | US | 1972-1978 | Havard University and University of Pennsylvania Alumni | 96 | - | - | - | 384 | - | - | - | Physician-diagnosed PD | Structured questionnaire | None |
| 109 | 1991 | Wechsler | US | - | Neurology Clinic at University of Washington and PD support groups | 34 | - | - | 68.4 | 22 | - | - | 58.9 | Not specified | Structured questionnaire | None |
| 110 | 1992 | Busenbark | US | - | Movement Disorders Center, University of Kansas | 16 | 5 | 11 | 52.1 | 16 | 4 | 12 | 51.5 | 2 of 3 cardinal signs of PD | Questionnaire | None |
| 111 | 1992 | Jimenez-Jimenez | Spain | - | Hospital General Gregario Maranon, Madrid | 128 | 67 | 61 | 66.8 | 256 | 134 | 122 | 64.8 | Not specified | Structured questionnaire | None |
| 78 | 1993 | Semchuk | Canada | 1984-1987 | Calgary Residents Longterm Care Registry | 130 | 75 | 55 | 68.5 | 260 | 150 | 110 | 68.3 | Neurologist diagnosed PD | Structured interview | FHx PD, FHx ET, head trauma, herbicide use |
| 83 | 1993 | Wang | China | - | Department of Neurology, Tianjin General Hospital | 93 | 63 | 30 | 61 | 186 | 126 | 60 | 60 | Neurologist diagnosed PD (2 or more cardinal signs) | Structured interview | None |
| 112 | 1994 | Mayeux | US | 1988-1990 | Washington Heights and Inwood communities of New York City | 150 | 75 | 75 | 71.3 | 180 | 35 | 145 | 74.6 | Neurologist diagnosed | Structured interview | Age, gender |
| 113 | 1995 | Martyn | UK | - | 42 General Practices, Hertfordshire | 172 | 108 | 64 | 70.8 | 343 | 220 | 123 | 70.7 | Neurologist or geriatrician diagnosed PD | Structured questionnaire | None |
| 87 | 1996 | de Michele | Italy | - | Department of Neurology Federico II University in Naples | 116 | 77 | 39 | 62.5 | 232 | 116 | 116 | 62.4 | 2 of 3 cardinal signs of PD | Structured questionnaire | None |
| 114 | 1997 | Hellenbrand | Germany | 1987-1992 | 9 Neurology clinics in Germany | 380 | - | - | 56.2 | 379 | - | - | 56.5 | Neurologist diagnosed (UK Brain Bank Criteria) | Structured interview | Education |
| 115 | 1997 | Liou | China | 1993-1995 | Movement Disorder clinic, National Taiwan University Hospital | 120 | 65 | 55 | 63.1 | 240 | 130 | 110 | 63.5 | Neurologist diagnosed PD (2 of 4 cardinal signs) | Structured questionnaire | None |
| 116 | 1997 | Tzourino | Europe | - | EUROPARKINSON study | 193 | 82 | 111 | - | 535 | - | - | - | Neurologist diagnosed PD (2 of 4 cardinal signs) | Structured questionnaire | Age, sex, dementia |
| 89 | 1998 | Chan | Hong Kong | - | 2 Hospitals in Hong Kong | 215 | 114 | 101 | - | 313 | 171 | 142 | - | Neurologist diagnosed PD (Maranganore criteria) | Structured interview | Smoking, FHx, tea, rural living, dietary factors, farming |
| 117 | 1998 | de Palma | Italy | - | Institute of Neurology, University of Palma | 100 | 59 | 41 | 66.6 | 200 | 118 | 82 | 64.2 | UK Brain Bank Criteria | Structured questionnaire | None |
| 71 | 1998 | McCann | Australia | - | Clinics and Residential Care Centres, Queensland and New South Wales | 224 | 131 | 93 | 70.3 | 310 | 193 | 117 | 68.9 | PD diagnosed according to Calne criteria | Questionnaire | None |
| 118 | 1998 | Smargiassi | Italy | - | Emilia-Romagna Region, Italy | 86 | 50 | 36 | 66.4 | 86 | 48 | 38 | 63.1 | Neurologist diagnosed (UK Brain Bank Criteria) | Structured questionnaire | None |
| 119 | 1999 | Gorell | US | 1991-1995 | Henry Ford Health System Cohort, Detroit | 144 | - | - | - | 464 | - | - | - | Neurologist diagnosed PD | Interview with structured questionnaire | Age, sex, race |
| 120 | 1999 | Kuopio | Finland | 1994-1996 | Nine rural municipalities in Finland | 123 | 63 | 60 | 68.7 | 246 | 126 | 120 | 69.3 | Neurologist diagnosed | Structured interview | None |
| 90 | 1999 | Werneck | Brazil | 1996-1997 | Neurology Department of IASERJ Central Hospital | 92 | 41 | 51 | 70.6 | 110 | 47 | 63 | 68.4 | Neurologist diagnosed PD (Calne criteria) | Structured questionnaire | None |
| 74 | 2000 | Preux | France | 1995-1996 | Limoges University Hospital, France | 140 | - | - | 71.1 | 280 | - | - | 70.5 | Physician examined (UK Brain Bank Criteria) | Structured interview | FHx, coffee, tea, urban area, toxic products |
| 121 | 2000 | Vanacore | Europe | 1994-1998 | Multicentre European Study | 140 | 81 | 59 | 66.3 | 134 | 72 | 62 | 65.3 | UK Brain Bank Criteria | Structured interview | Sex, age, centre of recruitment |
| 91 | 2001 | Behari | India | 1994-1998 | Movement Disorder clinic of AIIMS, New Dehli | 377 | 301 | 76 | 56.8 | 377 | 271 | 106 | 56.6 | Neurologist diagnosed PD (2 of 3 cardinal signs) | Structured questionnaire | Age |
| 62 | 2001 | Herishanu | Israel | 1989-1995 | PD clinic of Soroka University Medical Centre | 93 | - | - | - | 93 | - | - | - | Progressive disorder, 2 or more cardinal signs of PD | Interview with structured questionnaire | None |
| 122 | 2001 | Paganini-Hill | US | 1981-1998 | Leisure World Cohort, California | 395 | - | - | 75 | 2320 | - | - | 75 | Medical record, death certificate and survey | Structured questionnaire | None |
| 82 | 2002 | Zorzon | Italy | 1998 | Center for Parkinson's disease and Movement Disorders, Trieste | 136 | 62 | 74 | 70 | 272 | 124 | 148 | 69 | Neurologist diagnosed PD (2 of 4 cardinal signs) | Structured interview | Smoking |
| 123 | 2003 | Baldereschi | Italy | 1992-1993 | Italian Longitudinal Study on Aging | 113 | 58 | 57 | 78.1 | 4383 | 2247 | 2136 | 74.5 | Medical record (2 of 4 cardinal signs) | Structured questionnaire | Age, education, pesticides |
| 124 | 2003 | Baldi | France | 1997-1999 | Gironde and Dordogne | 84 | 44 | 40 | 75.6 | 252 | 132 | 120 | 75.5 | UK Brain Bank Criteria | Structured interview | Education, smoking |
| 125 | 2003 | Dong | China | 1997-2001 | Rural communities in Beijing | 114 | 60 | 54 | 70.1 | 205 | 96 | 109 | 74 | Calne criteria | Structured questionnaire | None |
| 60 | 2003 | Duzcan | Turkey | 2000 | Kizilcaboluk-Denizli | 36 | 17 | 19 | - | 108 | 51 | 57 | - | Neurologist diagnosed Parkinsonism (2 of 4 cardinal signs) + levodopa response | Questionnaire | None |
| 126 | 2003 | Pals | Belgium | - | Patient support groups and 3 Flemish universities | 423 | 256 | 167 | 67 | 205 | 70 | 135 | - | Neurologist diagnosed (3 of 4 cardinal signs) | Structured questionnaire | Age, gender, FHx |
| 37 | 2003 | Ragonese | Italy | - | Neurological Clinics Palermo and Messina, Sicily | 150 | 68 | 82 | - | 150 | 68 | 82 | - | 2 of 4 cardinal signs | Structured questionnaire | Education |
| 248 | 2003 | Tan | Singapore | - | Singapore | 230 | 145 | 85 | 66 | 241 | 142 | 99 | 64 | Standardised diagnostic criteria | Questionnaire | None |
| 92 | 2005 | Galanaud | France | 1998-1999 | Mutualite Sociale Agricole, French health insurance system | 247 | 138 | 109 | 69 | 676 | 377 | 299 | 69 | Neurologist diagnosed PD (2 or more cardinal signs) | Structured interview | Age, education |
| 128 | 2006 | Ma | China | 1985-2000 | Linxian, Henan Province | 85 | 40 | 45 | - | 340 | 160 | 180 | - | Neurologist diagnosed (UK Brain Bank Criteria) | Structured questionnaire | None |
| 129 | 2006 | Powers | US | 1992-2005 | Group Health Cooperative, Seattle area | 352 | 217 | 135 | 69 | 484 | 298 | 186 | 71 | Neurologist diagnosed or medical record review | Structured questionnaire | Age, ethnicity, coffee, NSAIDs, state |
| 59 | 2007 | Dick | UK | 2000-2004 | GEOPARKINSON study | 649 | - | - | - | 1587 | - | - | - | Physician/medical record (UK Brain Bank criteria) | Structured interview | Age, sex, country, unconscious, 1st degree relative |
| 130 | 2007 | Frigerio | US | 1976-1995 | Rochester Epidemiology Project | 193 | 120 | 73 | - | 193 | 120 | 73 | - | 2 of 4 cardinal signs | Structured interview | Age, sex |
| 131 | 2007 | Kamel | US | 1993-2003 | Agricultural Health Study | 78 | - | - | - | 55931 | - | - | - | Physician diagnosed PD | Structured questionnaire | None |
| 49 | 2008 | Becker | UK | 1994-2005 | General Practice Research Database, UK | 3637 | 2167 | 1470 | - | 3637 | 2167 | 1470 | - | Read-coded diagnosis of PD in database | Drug prescriptions in the database | None |
| 132 | 2008 | Facheris | US | 1996-2006 | Department of Neurology, Mayo Clinic, Rochester | 158 | 93 | 65 | 63.1 | 158 | 93 | 65 | 62.4 | Neurologist diagnosed PD | Structured interview | None |
| 133 | 2008 | Petersen | Faroe Islands | 2005 | Faroe Islands | 79 | 43 | 36 | 74.4 | 154 | 85 | 69 | 75.2 | Neurologist diagnosed | Structured questionnaire | Smoking |
| 134 | 2008 | Powers | US | 1996-2005 | NeuroGenetics Research Consortium (NGRC) | 1186 | 790 | 396 | 69.6 | 928 | 374 | 554 | 70.6 | Neurologist diagnosed PD | Structured questionnaire | Age, ethnicity, coffee, NSAIDs, state |
| 135 | 2009 | D'Amelio | Italy | - | Neurological Department, Palermo | 318 | 153 | 165 | 66.7 | 318 | 153 | 168 |  | 2 of 4 cardinal signs | Structured questionnaire | None |
| 136 | 2009 | Gatto | US | 2001-2007 | Parkinson's Environment and Genes Study | 368 | 207 | 161 | - | 341 | 176 | 165 | - | Neurologist diagnosed PD (2 of 4 cardinal signs and absence of atypical features) | Telephone interview | None |
| 137 | 2009 | Tanner | US | 2004-2007 | 8 North American Movement Disorder clinics | 519 | 309 | 210 | 65 | 511 | 302 | 209 | 65 | Neurologist diagnosed PD (2 of 4 cardinal signs) | Structured interview | Age, sex, race, smoking, caffeine, alcohol, head injury |
| 138 | 2010 | Fang | US | 1995-2006 | NIH-AARP Diet and Health Study | 992 | 744 | 248 | 64.4 | 279958 | 164491 | 115467 | 61.8 | Neurologist diagnosed | Structured questionnaire | None |
| 93 | 2010 | Sanyal | India | - | Movement Disorders clinic in Kolkata | 175 | 140 | 35 | 55.2 | 350 | 280 | 70 | 55 | 3 of 4 cardinal signs of PD | Structured interview | FHx, pesticide exposure, rural living, depression, other toxins |
| 249 | 2010 | Skeie | Norway | 2004-2006 | 4 counties in Norway | 212 | 126 | 86 | - | 175 | 104 | 71 | 67.5 | Gelb diagnostic criteria | Structured interview | None |
| 139 | 2010 | Tanaka | Japan | - | 4 Japanese hospitals | 249 | 93 | 156 | 68.5 | 369 | 141 | 228 | 66.6 | Neurologist diagnosed (UK Brain Bank Criteria) | Structured questionnaire | Age, sex, region, education, occupational exposure |
|  |  |  |  |  |  |  |  |  |  |  |  |  |  |  |  |  |
|  | Cohort studies - Ever smoking versus never | | | | | |  |  |  |  |  |  |  |  |  |  |
|  |  |  |  |  |  |  |  |  |  |  |  |  |  |  |  |  |
| **Ref** | **Year** | **Author** | **Country** | **Mean follow up (yrs)** | **Resource** | **Cohort** | **Male** | **Female** | **Cases** | **Male** | **Female** | **Mean Age** |  | **Definition** | **Exposure Assessment** | **Matching for** |
| 36 | 1996 | Morens | US | 29 | Honolulu Heart Program | 8006 | 8006 | - | 92 | 92 | - | - |  | Neurologist diagnosed | Structured questionnaire | Age |
| 143 | 2001 | Hernan | US | - | HPFS & NHS | 173229 | 51529 | 121700 | 280 | 128 | 152 | - |  | Neurologist diagnosed | Structured questionnaire | Age |
| 144 | 2007 | Thacker | US | - | Cancer Prevention Study II Nutrition Cohort | 143325 | 63348 | 79977 | 413 | 263 | 142 | - |  | Neurologist diagnosed | Structured questionnaire | Age |
| 145 | 2008 | Tan | Singapore | 7 | Singapore Chinese Health Study | 63257 | - | - | 157 | 83 | 74 | 61.8 |  | Medical record review | Structured questionnaire | Age, year of interview, gender, dialect, education |
| 146 | 2010 | Chen | US | - | NIH-AARP DH Cohort | 305468 | 178955 | 126513 | 1662 | 1228 | 434 | 63.8 |  | Neurologist diagnosed | Structured questionnaire | Age, race, caffeine, gender |
|  |  |  |  |  |  |  |  |  |  |  |  |  |  |  |  |  |
|  |  |  |  |  |  |  |  |  |  |  |  |  |  |  |  |  |
|  | Case control studies - Current smoking versus never | | | | | |  |  |  |  |  |  |  |  |  |  |
|  |  |  |  |  |  |  |  |  |  |  |  |  |  |  |  |  |
| **Ref** | **Year** | **First Author** | **Country** | **Study period** | **Resource** | **Cases** | **Male** | **Female** | **Mean Age** | **Controls** | **Male** | **Female** | **Mean Age** | **Definition** | **Exposure assessment** | **Matching for** |
| 96 | 1968 | Nefzger | US | - | Veterans Administration Central Office, Washington | 198 | 198 | - | - | 198 | 198 | - | - | Neurologist diagnosed PD | Structured interview | None |
| 98 | 1971 | Kessler | US | 1965-1969 | Balitmore | 468 | 243 | 225 | - | 468 | 243 | 225 | - | Physician-diagnosed PD | Structured interview | None |
| 100 | 1980 | Marttila | Finland | 1972-1973 | Southwestern Finland | 443 | - | - | - | 443 | - | - | - | Not specified | Structured interview | None |
| 101 | 1982 | Godwin-Austen | UK | - | General Practices around the UK | 350 | 179 | 171 | - | 350 | 179 | 171 | - | Not specified | Questionnaire | None |
| 102 | 1987 | Rajput | US | 1967-1979 | Rochester Residents | 118 | 54 | 64 | - | 236 | 108 | 128 | - | Medical record review | Medical record review | None |
| 105 | 1989 | Hofman | Holland | 1981-1986 | Sentinel Practices of the Netherlands Institute for General Practice | 86 | - | - | - | 172 | - | - | - | Neurologist diagnosed | Structured questionnaire | Age, gender |
| 108 | 1990 | Sasco | US | 1972-1978 | Havard University and University of Pennsylvania Alumni | 96 | - | - | - | 384 | - | - | - | Physician-diagnosed PD | Structured questionnaire | None |
| 112 | 1994 | Mayeux | US | 1988-1990 | Washington Heights and Inwood communities of New York City | 150 | 75 | 75 | 71.3 | 180 | 35 | 145 | 74.6 | Neurologist diagnosed | Structured interview | Age, gender |
| 113 | 1995 | Martyn | UK | - | 42 General Practices, Hertfordshire | 172 | 108 | 64 | 70.8 | 343 | 220 | 123 | 70.7 | Neurologist or geriatrician diagnosed PD | Structured questionnaire | None |
| 114 | 1997 | Hellenbrand | Germany | 1987-1992 | 9 Neurology clinics in Germany | 380 | - | - | 56.2 | 379 | - | - | 56.5 | Neurologist diagnosed (UK Brain Bank Criteria) | Structured interview | Education |
| 116 | 1997 | Tzourino | Europe | - | EUROPARKINSON study | 193 | 82 | 111 | - | 535 | - | - | - | Neurologist diagnosed PD (2 of 4 cardinal signs) | Structured questionnaire | Age, sex, dementia |
| 89 | 1998 | Chan | Hong Kong | - | 2 Hospitals in Hong Kong | 215 | 114 | 101 | - | 313 | 171 | 142 | - | Neurologist diagnosed PD (Maranganore criteria) | Structured interview | Pesticides, FHx, tea, rural living, dietary factors, farming |
| 120 | 1999 | Kuopio | Finland | 1994-1996 | Nine rural municipalities in Finland | 123 | 63 | 60 | 68.7 | 246 | 126 | 120 | 69.3 | Neurologist diagnosed | Structured interview | None |
| 246 | 2000 | Benedetti | US | 1976-1995 | Rochester Epidemiology Project | 196 | 121 | 75 | - | 196 | 121 | 75 | - | 2 of 4 cardinal signs | Structured interview | None |
| 122 | 2001 | Paganini-Hill | US | 1981-1998 | Leisure World Cohort, California | 395 | - | - | 75 | 2320 | - | - | 75 | Medical record, death certificate and survey | Structured questionnaire | None |
| 141 | 2002 | Checkoway | US | 1992-2000 | Group Health Cooperative, Washington State | 210 | 131 | 79 | 70 | 347 | 225 | 122 | 71 | 2 of 4 cardinal signs | Structured questionnaire | Age, ethnicity, education, gender |
| 125 | 2003 | Dong | China | 1997-2001 | Rural communities in Beijing | 114 | 60 | 54 | 70.1 | 205 | 96 | 109 | 74 | Calne criteria | Structured questionnaire | None |
| 37 | 2003 | Ragonese | Italy | - | Neurological Clinics Palermo and Messina, Sicily | 150 | 68 | 82 | - | 150 | 68 | 82 | - | 2 of 4 cardinal signs | Structured questionnaire | Education |
| 92 | 2005 | Galanaud | France | 1998-1999 | Mutualite Sociale Agricole, French health insurance system | 247 | 138 | 109 | 69 | 676 | 377 | 299 | 69 | Neurologist diagnosed PD (2 or more cardinal signs) | Structured interview | Age, sex, education, alcohol, farming, pesticides, smoking |
| 131 | 2007 | Kamel | US | 1993-2003 | Agricultural Health Study | 78 | - | - | - | 55931 | - | - | - | Physician diagnosed PD | Structured questionnaire | Age, state, participant |
| 49 | 2008 | Becker | UK | 1994-2005 | General Practice Research Database, UK | 3637 | 2167 | 1470 | - | 3637 | 2167 | 1470 | - | Read-coded diagnosis of PD in database | Drug prescriptions in the database | Age, sex, BMI, co-morbidity |
| 133 | 2008 | Petersen | Faroe Islands | 2005 | Faroe Islands | 79 | 43 | 36 | 74.4 | 154 | 85 | 69 | 75.2 | Neurologist diagnosed | Structured questionnaire | Age, sex |
| 134 | 2008 | Powers | US | 1996-2005 | NeuroGenetics Research Consortium (NGRC) | 1186 | 790 | 396 | 69.6 | 928 | 374 | 554 | 70.6 | Neurologist diagnosed PD | Structured questionnaire | Age, ethnicity, coffee, NSAIDs, state |
| 136 | 2009 | Gatto | US | 2001-2007 | Parkinson's Environment and Genes Study | 368 | 207 | 161 | - | 341 | 176 | 165 | - | Neurologist diagnosed PD (2 of 4 cardinal signs and absence of atypical features) | Telephone interview | None |
| 138 | 2010 | Fang | US | 1995-2006 | NIH-AARP Diet and Health Study | 992 | 744 | 248 | 64.4 | 279958 | 164491 | 115467 | 61.8 | Neurologist diagnosed | Structured questionnaire | None |
| 139 | 2010 | Tanaka | Japan | - | 4 Japanese hospitals | 249 | 93 | 156 | 68.5 | 369 | 141 | 228 | 66.6 | Neurologist diagnosed (UK Brain Bank Criteria) | Structured questionnaire | Sex, age, region of residence, education, occupational exposure |
|  |  |  |  |  |  |  |  |  |  |  |  |  |  |  |  |  |
|  | Cohort studies - Current smoking versus never | | | | | |  |  |  |  |  |  |  |  |  |  |
|  |  |  |  |  |  |  |  |  |  |  |  |  |  |  |  |  |
| **Ref** | **Year** | **Author** | **Country** | **Mean follow up (yrs)** | **Resource** | **Cohort** | **Male** | **Female** | **Cases** | **Male** | **Female** | **Mean Age** |  | **Definition** | **Exposure Assessment** | **Matching for** |
| 143 | 2001 | Hernan | US | - | HPFS & NHS | 173229 | 51529 | 121700 | 280 | 128 | 152 | - |  | Neurologist diagnosed | Structured questionnaire | Age |
| 144 | 2007 | Thacker | US | - | Cancer Prevention Study II Nutrition Cohort | 143325 | 63348 | 79977 | 413 | 263 | 142 | - |  | Neurologist diagnosed | Structured questionnaire | Age, sex |
| 142 | 2008 | Saaksjarvi | Finland | 22 | Finnish Mobile Clinic Health Examination Survey | 6710 | 3192 | 3518 | 101 | 46 | 55 | 62.7 |  | Neurologist diagnosed | Structured questionnaire | Age, sex |
| 145 | 2008 | Tan | Singapore | 7 | Singapore Chinese Health Study | 63257 | - | - | 157 | 83 | 74 | 61.8 |  | Medical record review | Structured questionnaire | Age, year, gender, dialect, education |
| 140 | 2009 | Chen | US | - | ARIC Cohort | 15036 | - | - | 95 | - | - | - |  | Medical record review | Structured questionnaire | Age, sex, race |
| 146 | 2010 | Chen | US | - | NIH-AARP DH Cohort | 305468 | 178955 | 126513 | 1662 | 1228 | 434 | 63.8 |  | Neurologist diagnosed | Structured questionnaire | Age, race, caffeine intake, gender |
|  |  |  |  |  |  |  |  |  |  |  |  |  |  |  |  |  |
|  |  |  |  |  |  |  |  |  |  |  |  |  |  |  |  |  |
|  | Case control studies - Past smoking versus never | | | | | |  |  |  |  |  |  |  |  |  |  |
|  |  |  |  |  |  |  |  |  |  |  |  |  |  |  |  |  |
| **Ref** | **Year** | **First Author** | **Country** | **Study period** | **Resource** | **Cases** | **Male** | **Female** | **Mean Age** | **Controls** | **Male** | **Female** | **Mean Age** | **Definition** | **Exposure assessment** | **Matching for** |
| 96 | 1968 | Nefzger | US | - | Veterans Administration Central Office, Washington | 198 | 198 | - | - | 198 | 198 | - | - | Neurologist diagnosed PD | Structured interview | None |
| 98 | 1971 | Kessler | US | 1965-1969 | Balitmore | 468 | 243 | 225 | - | 468 | 243 | 225 | - | Physician-diagnosed PD | Structured interview | None |
| 100 | 1980 | Marttila | Finland | 1972-1973 | Southwestern Finland | 443 | - | - | - | 443 | - | - | - | Not specified | Structured interview | None |
| 101 | 1982 | Godwin-Austen | UK | - | General Practices around the UK | 350 | 179 | 171 | - | 350 | 179 | 171 | - | Not specified | Questionnaire | None |
| 102 | 1987 | Rajput | US | 1967-1979 | Rochester Residents | 118 | 54 | 64 | - | 236 | 108 | 128 | - | Medical record review | Medical record review | None |
| 105 | 1989 | Hofman | Holland | 1981-1986 | Sentinel Practices of the Netherlands Institute for General Practice | 86 | - | - | - | 172 | - | - | - | Neurologist diagnosed | Structured questionnaire | Age, gender |
| 108 | 1990 | Sasco | US | 1972-1978 | Havard University and University of Pennsylvania Alumni | 96 | - | - | - | 384 | - | - | - | Physician-diagnosed PD | Structured questionnaire | None |
| 112 | 1994 | Mayeux | US | 1988-1990 | Washington Heights and Inwood communities of New York City | 150 | 75 | 75 | 71.3 | 180 | 35 | 145 | 74.6 | Neurologist diagnosed | Structured interview | Age, gender |
| 113 | 1995 | Martyn | UK | - | 42 General Practices, Hertfordshire | 172 | 108 | 64 | 70.8 | 343 | 220 | 123 | 70.7 | Neurologist or geriatrician diagnosed PD | Structured questionnaire | None |
| 114 | 1997 | Hellenbrand | Germany | 1987-1992 | 9 Neurology clinics in Germany | 380 | - | - | 56.2 | 379 | - | - | 56.5 | Neurologist diagnosed (UK Brain Bank Criteria) | Structured interview | Education |
| 116 | 1997 | Tzourino | Europe | - | EUROPARKINSON study | 193 | 82 | 111 | - | 535 | - | - | - | Neurologist diagnosed PD (2 of 4 cardinal signs) | Structured questionnaire | Age, sex, dementia |
| 89 | 1998 | Chan | Hong Kong | - | 2 Hospitals in Hong Kong | 215 | 114 | 101 | - | 313 | 171 | 142 | - | Neurologist diagnosed PD (Maranganore criteria) | Structured interview | Pesticides, FHx, tea, rural living, dietary factors, farming |
| 120 | 1999 | Kuopio | Finland | 1994-1996 | Nine rural municipalities in Finland | 123 | 63 | 60 | 68.7 | 246 | 126 | 120 | 69.3 | Neurologist diagnosed | Structured interview | None |
| 246 | 2000 | Benedetti | US | 1976-1995 | Rochester Epidemiology Project | 196 | 121 | 75 | - | 196 | 121 | 75 | - | 2 of 4 cardinal signs | Structured interview | None |
| 122 | 2001 | Paganini | US | 1981-1998 | Lesiure World Cohort, California | 395 | - | - | 75 | 2320 | - | - | 75 | Medical record, death certificate and survey | Structured questionnaire | None |
| 141 | 2002 | Checkoway | US | 1992-2000 | Group Health Cooperative, Washington State | 210 | 131 | 79 | 70 | 347 | 225 | 122 | 71 | 2 of 4 cardinal signs | Structured questionnaire | Age, ethnicity, education, gender |
| 125 | 2003 | Dong | China | 1997-2001 | Rural communities in Beijing | 114 | 60 | 54 | 70.1 | 205 | 96 | 109 | 74 | Calne criteria | Structured questionnaire | None |
| 37 | 2003 | Ragonese | Italy | - | Neurological Clinics Palermo and Messina, Sicily | 150 | 68 | 82 | - | 150 | 68 | 82 | - | 2 of 4 cardinal signs | Structured questionnaire | Education |
| 92 | 2005 | Galanaud | France | 1998-1999 | Mutualite Sociale Agricole, French health insurance system | 247 | 138 | 109 | 69 | 676 | 377 | 299 | 69 | Neurologist diagnosed PD (2 or more cardinal signs) | Structured interview | Age, sex, education, alcohol, farming, pesticides, smoking |
| 131 | 2007 | Kamel | US | 1993-2003 | Agricultural Health Study | 78 | - | - | - | 55931 | - | - | - | Physician diagnosed PD | Structured questionnaire | Age, state, participant |
| 49 | 2008 | Becker | UK | 1994-2005 | General Practice Research Database, UK | 3637 | 2167 | 1470 | - | 3637 | 2167 | 1470 | - | Read-coded diagnosis of PD in database | Drug prescriptions in the database | Age, sex, BMI, co-morbidity |
| 133 | 2008 | Petersen | Faroe Islands | 2005 | Faroe Islands | 79 | 43 | 36 | 74.4 | 154 | 85 | 69 | 75.2 | Neurologist diagnosed | Structured questionnaire | Age, sex |
| 134 | 2008 | Powers | US | 1996-2005 | NeuroGenetics Research Consortium (NGRC) | 1186 | 790 | 396 | 69.6 | 928 | 374 | 554 | 70.6 | Neurologist diagnosed PD | Structured questionnaire | Age, ethnicity, coffee, NSAIDs, state |
| 136 | 2009 | Gatto | US | 2001-2007 | Parkinson's Environment and Genes Study | 368 | 207 | 161 | - | 341 | 176 | 165 | - | Neurologist diagnosed PD (2 of 4 cardinal signs and absence of atypical features) | Telephone interview | None |
| 138 | 2010 | Fang | US | 1995-2006 | NIH-AARP Diet and Health Study | 992 | 744 | 248 | 64.4 | 279958 | 164491 | 115467 | 61.8 | Neurologist diagnosed | Structured questionnaire | None |
| 139 | 2010 | Tanaka | Japan | - | 4 Japanese hospitals | 249 | 93 | 156 | 68.5 | 369 | 141 | 228 | 66.6 | Neurologist diagnosed (UK Brain Bank Criteria) | Structured questionnaire | Sex, age, region of residence, education, occupational exposure |
|  |  |  |  |  |  |  |  |  |  |  |  |  |  |  |  |  |
|  | Cohort studies - Past smoking versus never | | | | | |  |  |  |  |  |  |  |  |  |  |
|  |  |  |  |  |  |  |  |  |  |  |  |  |  |  |  |  |
| **Ref** | **Year** | **Author** | **Country** | **Mean follow up (yrs)** | **Resource** | **Cohort** | **Male** | **Female** | **Cases** | **Male** | **Female** | **Mean Age** |  | **Definition** | **Exposure Assessment** | **Matching for** |
| 143 | 2001 | Hernan | US | - | HPFS & NHS | 173229 | 51529 | 121700 | 280 | 128 | 152 | - |  | Neurologist diagnosed | Structured questionnaire | Age |
| 144 | 2007 | Thacker | US | - | Cancer Prevention Study II Nutrition Cohort | 143325 | 63348 | 79977 | 413 | 263 | 142 | - |  | Neurologist diagnosed | Structured questionnaire | Age, sex |
| 145 | 2008 | Tan | Singapore | 7 | Singapore Chinese Health Study | 63257 | - | - | 157 | 83 | 74 | 61.8 |  | Medical record review | Structured questionnaire | Age, year, gender, dialect, education |
| 146 | 2010 | Chen | US | - | NIH-AARP DH Cohort | 305468 | 178955 | 126513 | 1662 | 1228 | 434 | 63.8 |  | Neurologist diagnosed | Structured questionnaire | Age, race, caffeine intake, gender |
|  |  |  |  |  |  |  |  |  |  |  |  |  |  |  |  |  |
|  |  |  |  |  |  |  |  |  |  |  |  |  |  |  |  |  |
|  | Case control studies - Coffee drinking versus non-drinking | | | | | |  |  |  |  |  |  |  |  |  |  |
|  |  |  |  |  |  |  |  |  |  |  |  |  |  |  |  |  |
| **Ref** | **Year** | **First Author** | **Country** | **Study period** | **Resource** | **Cases** | **Male** | **Female** | **Mean Age** | **Controls** | **Male** | **Female** | **Mean Age** | **Definition** | **Exposure assessment** | **Matching for** |
| 96 | 1968 | Nefzger | US | - | Veterans Administration Central Office, Washington | 198 | 198 | - | - | 198 | 198 | - | - | Neurologist diagnosed PD | Structured interview | None |
| 111 | 1992 | Jimenez-Jimenez | Spain | - | Hospital General Gregario Maranon, Madrid | 128 | 67 | 61 | 66.8 | 256 | 134 | 122 | 64.8 | Not specified | Structured questionnaire | None |
| 84 | 1994 | Morano | Spain | 1989-1990 | General Hospitals in Caceres, Spain | 74 | 33 | 41 | 68.2 | 148 | 66 | 82 | 67.5 | Diagnostic criteria for PD | Structured questionnaire | None |
| 74 | 2000 | Preux | France | 1995-1996 | Limoges University Hospital, France | 140 | - | - | - | 280 | - | - | - | Physician examined (UK Brain Bank Criteria) | Structured interview | Smoking, FHx, tea, urban area, toxic products |
| 122 | 2001 | Paganini-Hill | US | 1981-1998 | Lesiure World Cohort, California | 395 | - | - | 75 | 2320 | - | - | 75 | Medical record, death certificate and survey | Structured questionnaire | None |
| 37 | 2003 | Ragonese | Italy |  | Neurological Clinics Palermo and Messina, Sicily | 150 | 68 | 82 | - | 150 | 68 | 82 | - | 2 of 4 cardinal signs | Structured questionnaire | Education |
| 147 | 2004 | Nuti | Italy | - | Lucca and Pistoia, Tuscany | 190 | 106 | 84 | 63.9 | 190 | 106 | 84 | 62.8 | UK Brain Bank Criteria | Structured interview | None |
| 130 | 2007 | Frigerio | US | 1976-1995 | Rochester Epidemiology Project | 193 | 120 | 73 | - | 193 | 120 | 73 | - | 2 of 4 cardinal signs | Structured interview | Age, sex |
| 132 | 2008 | Facheris | US | 1996-2006 | Department of Neurology, Mayo Clinic, Rochester | 158 | 93 | 65 | 63.1 | 158 | 93 | 65 | 62.4 | Neurologist diagnosed PD | Structured interview | Age, gender |
| 134 | 2008 | Powers | US | 1996-2005 | NeuroGenetics Research Consortium (NGRC) | 1186 | 790 | 396 | 69.6 | 928 | 374 | 554 | 70.6 | Neurologist diagnosed PD | Structured questionnaire | Smoking, coffee, age, ethnicity and state |
| 135 | 2009 | D'Amelio | Italy | - | Neurological Department, Palermo | 318 | 153 | 165 | 66.7 | 318 | 153 | 168 |  | 2 of 4 cardinal signs | Structured questionnaire | None |
| 137 | 2009 | Tanner | US | 2004-2007 | 8 North American Movement Disorder clinics | 519 | 309 | 210 | 65 | 511 | 302 | 209 | 65 | Neurologist diagnosed PD (2 of 4 cardinal signs) | Structured interview | None |
| 249 | 2010 | Skeie | Norway | 2004-2006 | 4 counties in Norway | 212 | 126 | 86 | - | 175 | 104 | 71 | 67.5 | Gelb diagnostic criteria | Structured interview | None |
|  |  |  |  |  |  |  |  |  |  |  |  |  |  |  |  |  |
|  | Cohort studies - Coffee drinking versus non-drinking | | | | | |  |  |  |  |  |  |  |  |  |  |
|  |  |  |  |  |  |  |  |  |  |  |  |  |  |  |  |  |
| **Ref** | **Year** | **Author** | **Country** | **Mean follow up (yrs)** | **Resource** | **Cohort** | **Male** | **Female** | **Cases** | **Male** | **Female** | **Mean Age** |  | **Definition** | **Exposure Assessment** | **Matching for** |
| 148 | 2000 | Ross | Hawaii | 27 | Honolulu Heart Program | 8004 | 8004 | - | 102 | 102 | - | - |  | Diagnosed by 2 neurologists | Dietary recall questionnaire | Age, smoking |
| 149 | 2001 | Ascherio | US | 10 | Health Professionals' Follow Up Study | 47351 | 47351 | - | 157 | 157 | - | - |  | Neurologist diagnosed | Structured questionnaire | Age, smoking, BMI, alcohol, physical activity |
| 149 | 2001 | Ascherio | US | 16 | Nurses' Health Study | 88565 | - | 88565 | 131 | - | 131 | - |  | Neurologist diagnosed | Structured questionnaire | Age, smoking, BMI, alcohol, physical activity |
| 150 | 2004 | Ascherio | US | - | Cancer Prevention Study II | 539222 | 301164 | 238058 | 1249 | 909 | 340 | - |  | Death certificates including PD as a diagnosis | Structured questionnaire | None |
| 151 | 2007 | Hu | Finland | 12.9 | Population surveys | 29335 | 14293 | 15042 | 200 | 102 | 98 | - |  | Neurologist diagnosed | Structured questionnaire | Age, study year, BMI, BP, cholesterol, education, physical activity, smoking, alcohol, tea, diabetes mellitus |
| 142 | 2008 | Saaksjarvi | Finland | 22 | Finnish Mobile Clinic Health Examination Survey | 6710 | 3192 | 3518 | 101 | 46 | 55 | 62.7 |  | Neurologist diagnosed | Structured questionnaire | Age, sex, marital status, education, community density, alcohol, physical activity, smoking, BMI |
|  |  |  |  |  |  |  |  |  |  |  |  |  |  |  |  |  |
|  |  |  |  |  |  |  |  |  |  |  |  |  |  |  |  |  |
|  | Case control studies - Alcohol drinking versus non-drinking | | | | | |  |  |  |  |  |  |  |  |  |  |
|  |  |  |  |  |  |  |  |  |  |  |  |  |  |  |  |  |
| **Ref** | **Year** | **First Author** | **Country** | **Study period** | **Resource** | **Cases** | **Male** | **Female** | **Mean Age** | **Controls** | **Male** | **Female** | **Mean Age** | **Definition** | **Exposure assessment** | **Matching for** |
| 104 | 1989 | Ho | Hong Kong | - | 10 Old Age Homes in Shatin and Tai Po | 35 | 11 | 24 | - | 105 | - | - | - | Trained medical students using diagnostic criteria | Structured interview | None |
| 111 | 1992 | Jimenez-Jimenez | Spain | - | Hospital General Gregario Maranon, Madrid | 128 | 67 | 61 | 66.8 | 256 | 134 | 122 | 64.8 | Not specified | Structured questionnaire | None |
| 84 | 1994 | Morano | Spain | 1989-1990 | General Hospitals in Caceres, Spain | 74 | 33 | 41 | 68.2 | 148 | 66 | 82 | 67.5 | Diagnostic criteria for PD | Structured questionnaire | None |
| 112 | 1994 | Mayeux | US | 1988-1990 | Washington Heights and Inwood communities of New York City | 150 | 75 | 75 | 71.3 | 180 | 35 | 145 | 74.6 | Neurologist diagnosed | Structured interview | Age, gender |
| 115 | 1997 | Liou | China | 1993-1995 | Movement Disorder clinic, National Taiwan University Hospital | 120 | 65 | 55 | 63.1 | 240 | 130 | 110 | 63.5 | Neurologist diagnosed PD (2 of 4 cardinal signs) | Structured questionnaire | None |
| 118 | 1998 | Smargiassi | Italy | - | Emilia-Romagna Region, Italy | 86 | 50 | 36 | 66.4 | 86 | 48 | 38 | 63.1 | Neurologist diagnosed (UK Brain Bank Criteria) | Structured questionnaire | None |
| 119 | 1999 | Gorell | US | 1991-1995 | Henry Ford Health System Cohort, Detroit | 144 | - | - | - | 464 | - | - | - | Neurologist diagnosed PD | Interview with structured questionnaire | None |
| 91 | 2001 | Behari | India | 1994-1998 | Movement Disorder clinic of AIIMS, New Dehli | 377 | 301 | 76 | 56.8 | 377 | 271 | 106 | 56.6 | Neurologist diagnosed PD (2 of 3 cardinal signs) | Structured questionnaire | Age |
| 122 | 2001 | Paganini-Hill | US | 1981-1998 | Lesiure World Cohort, California | 395 | - | - | 75 | 2320 | - | - | 75 | Medical record, death certificate and survey | Structured questionnaire | None |
| 141 | 2002 | Checkoway | US | 1992-2000 | Group Health Cooperative, Washington State | 210 | 131 | 79 | 70 | 347 | 225 | 122 | 71 | 2 of 4 cardinal signs | Structured questionnaire | None |
| 37 | 2003 | Ragonese | Italy | - | Neurological Clinics Palermo and Messina, Sicily | 150 | 68 | 82 | - | 150 | 68 | 82 | - | 2 of 4 cardinal signs | Structured questionnaire | Education |
| 60 | 2003 | Duzcan | Turkey | 2000 | Kizilcaboluk-Denizli | 36 | 17 | 19 | - | 108 | 51 | 57 | - | Neurologist diagnosed Parkinsonism (2 of 4 cardinal signs) + levodopa response | Questionnaire | None |
| 152 | 2004 | Hernan | UK | 1995-2000 | General Practice Research Database | 1019 | - | - | - | 10123 | - | - | - | 2 of 4 cardinal signs | Computer record review | None |
| 92 | 2005 | Galanaud | France | 1998-1999 | Mutualite Sociale Agricole, French health insurance system | 247 | 138 | 109 | 69 | 676 | 377 | 299 | 69 | Neurologist diagnosed PD (2 or more cardinal signs) | Structured interview | Age, Education |
| 59 | 2007 | Dick | UK | 2000-2004 | GEOPARKINSON study | 649 | - | - | - | 1587 | - | - | - | Physician/medical record (UK Brain Bank criteria) | Structured interview | Age, sex, country, tobacco, unconscious, 1st degree relative |
| 130 | 2007 | Frigerio | US | 1976-1995 | Rochester Epidemiology Project | 193 | 120 | 73 | - | 193 | 120 | 73 | - | 2 of 4 cardinal signs | Structured interview | Age, sex |
| 131 | 2007 | Kamel | US | 1993-2003 | Agricultural Health Study | 78 | - | - | - | 55931 | - | - | - | Physician diagnosed PD | Structured questionnaire | Age, state, type of participant |
| 153 | 2009 | Brighina | US | 1996-2006 | Department of Neurology, Mayo Clinic in Rochester, MN | 893 | 557 | 336 | 67.9 | 893 | 490 | 403 | 67.2 | Neurologist diagnosed PD | Structured interview | Age, sex, education, smoking, coffee |
| 135 | 2009 | D'Amelio | Italy | - | Neurological Department, Palermo | 318 | 153 | 165 | 66.7 | 318 | 153 | 168 |  | 2 of 4 cardinal signs | Structured questionnaire | None |
| 137 | 2009 | Tanner | US | 2004-2007 | 8 North American Movement Disorder clinics | 519 | 309 | 210 | 65 | 511 | 302 | 209 | 65 | Neurologist diagnosed PD (2 of 4 cardinal signs) | Structured interview | None |
| 247 | 2010 | Fukushima | Japan | 2006-2008 | 11 hospitals in Japan | 214 | 73 | 141 | 67.9 | 327 | 114 | 213 | 66.4 | Neurologist diagnosed (UK Brain Bank Criteria) | Structured questionnaire | None |
| 249 | 2010 | Skeie | Norway | 2004-2006 | 4 counties in Norway | 212 | 126 | 86 | - | 175 | 104 | 71 | 67.5 | Gelb diagnostic criteria | Structured interview | None |
|  |  |  |  |  |  |  |  |  |  |  |  |  |  |  |  |  |
|  | Cohort studies - Alcohol drinking versus non-drinking | | | | | |  |  |  |  |  |  |  |  |  |  |
|  |  |  |  |  |  |  |  |  |  |  |  |  |  |  |  |  |
| **Ref** | **Year** | **Author** | **Country** | **Mean follow up (yrs)** | **Resource** | **Cohort** | **Male** | **Female** | **Cases** | **Male** | **Female** | **Mean Age** |  | **Definition** | **Exposure Assessment** | **Matching for** |
| 38 | 1994 | Grandinetti | US | 26 | Honolulu Heart Program | 8006 | 8006 | - | 58 | 58 | - | - |  | Neurologist diagnosed | Structured questionnaire | Smoking, coffee, HTN, diabetes, cholesterol |
| 154 | 2003 | Hernan | US | - | HPFS and NHS | 136089 | 47367 | 88722 | 415 | 248 | 167 | - |  | Neurologist diagnosed | Structured questionnaire | Age, smoking, caffeine |
|  |  |  |  |  |  |  |  |  |  |  |  |  |  |  |  |  |
|  |  |  |  |  |  |  |  |  |  |  |  |  |  |  |  |  |
|  | Case control studies -Tea drinking versus non-drinking | | | | | |  |  |  |  |  |  |  |  |  |  |
|  |  |  |  |  |  |  |  |  |  |  |  |  |  |  |  |  |
| **Ref** | **Year** | **First Author** | **Country** | **Study period** | **Resource** | **Cases** | **Male** | **Female** | **Mean Age** | **Controls** | **Male** | **Female** | **Mean Age** | **Definition** | **Exposure assessment** | **Matching for** |
| 104 | 1989 | Ho | Hong Kong | - | 10 Old Age Homes in Shatin and Tai Po | 35 | 11 | 24 | - | 105 | - | - | - | Trained medical students using diagnostic criteria | Structured interview | None |
| 84 | 1994 | Morano | Spain | 1989-1990 | General Hospitals in Caceres, Spain | 74 | 33 | 41 | 68.2 | 148 | 66 | 82 | 67.5 | Diagnostic criteria for PD | Structured questionnaire | None |
| 74 | 2000 | Preux | France | 1995-1996 | Limoges University Hospital, France | 140 | - | - | - | 280 | - | - | - | Physician examined (UK Brain Bank Criteria) | Structured interview | Smoking, coffee, tea, urban area, toxic products |
| 197 | 2003 | Chan | Hong Kong | - | 2 Hospitals in Hong Kong | 215 | 114 | 101 | - | 313 | 171 | 142 | - | Neurologist diagnosed PD (Maranganore criteria) | Structured interview | Pesticide exposure, family history |
| 249 | 2010 | Skeie | Norway | 2004-2006 | 4 counties in Norway | 212 | 126 | 86 | - | 175 | 104 | 71 | 67.5 | Gelb diagnostic criteria | Structured interview | None |
|  |  |  |  |  |  |  |  |  |  |  |  |  |  |  |  |  |
|  | Cohort study - Tea drinking versus non-drinking | | | | | |  |  |  |  |  |  |  |  |  |  |
|  |  |  |  |  |  |  |  |  |  |  |  |  |  |  |  |  |
| **Ref** | **Year** | **Author** | **Country** | **Mean follow up (yrs)** | **Resource** | **Cohort** | **Male** | **Female** | **Cases** | **Male** | **Female** | **Mean Age** |  | **Definition** | **Exposure Assessment** | **Matching for** |
| 151 | 2007 | Hu | Finland | 12.9 | Population surveys | 29335 | 14293 | 15042 | 200 | 102 | 98 | - |  | Neurologist diagnosed | Structured questionnaire | None |
|  |  |  |  |  |  |  |  |  |  |  |  |  |  |  |  |  |
|  |  |  |  |  |  |  |  |  |  |  |  |  |  |  |  |  |
|  | Case control studies - Anxiety or depression preceding | | | | | |  |  |  |  |  |  |  |  |  |  |
|  |  |  |  |  |  |  |  |  |  |  |  |  |  |  |  |  |
| **Ref** | **Year** | **First Author** | **Country** | **Study period** | **Resource** | **Cases** | **Male** | **Female** | **Mean Age** | **Controls** | **Male** | **Female** | **Mean Age** | **Definition** | **Exposure assessment** | **Matching for** |
| 155 | 1993 | Hubble | US | - | Hayes and Kansas City | 63 | 34 | 29 | - | 75 | 34 | 41 | - | Neurologist diagnosed (2 of 4 cardinal signs) | Structured questionnaire | Pesticide use, neurological family history |
| 71 | 1998 | McCann | Australia | - | Clinics and Residential Care Centres, Queensland and New South Wales | 224 | 131 | 93 | 70.3 | 310 | 193 | 117 | 68.9 | PD diagnosed according to Calne criteria | Questionnaire | None |
| 81 | 1999 | Taylor | US | - | Movement Disorder Centre at Boston Medical Centre | 140 | 88 | 52 |  | 147 | 90 | 57 |  | Neurologist diagnosed PD (Ward and Gibb criteria) | Structured interview | Birth cohort, sex |
| 156 | 2000 | Shiba | US | 1976-1995 | Rochester Epidemiology Project | 196 | 121 | 75 | - | 196 | 121 | 75 | - | 2 of 4 cardinal signs | Structured interview | None |
| 91 | 2001 | Behari | India | 1994-1998 | Movement Disorder clinic of AIIMS, New Dehli | 377 | 301 | 76 | 56.8 | 377 | 271 | 106 | 56.6 | Neurologist diagnosed PD (2 of 3 cardinal signs) | Structured questionnaire | Age |
| 157 | 2003 | Leentjens | Holland | 1985-2000 | Registration Network Family Practices | 338 | 162 | 176 | - | 32077 | - | - | - | ICPC code for Parkinson's in database | ICPC code for depression in database | None |
| 59 | 2007 | Dick | UK | 2000-2004 | GEOPARKINSON study | 649 | - | - | - | 1587 | - | - | - | Physician/medical record (UK Brain Bank criteria) | Structured interview | Age, sex, country, smoking, unconscious, 1st degree relative |
| 158 | 2009 | Alonso | UK | 1995-2001 | General Practice Research Database, UK | 999 | - | - | - | 6261 | - | - | - | Computer diagnosis of PD in database | Drug prescriptions | Age, sex, practice, time of follow up |
| 63 | 2010 | Jacob | US | - | UCLA Parkinson's Environment and Genes (PEG) Study | 371 | 208 | 163 | 68.1 | 402 | 200 | 202 | 65.9 | Neurologist diagnosed (2 of 4 cardinal signs) | Structured interview | Age, sex, race, smoking, education |
| 93 | 2010 | Sanyal | India | - | Movement Disorders clinic in Kolkata | 175 | 140 | 35 | 55.2 | 350 | 280 | 70 | 55 | 3 of 4 cardinal signs of PD | Structured interview | Pesticides, rural living, FHx, toxins, smoking |
| 138 | 2010 | Fang | US | 1995-2006 | NIH-AARP Diet and Health Study | 992 | 744 | 248 | 64.4 | 279958 | 164491 | 115467 | 61.8 | Neurologist diagnosed | Structured questionnaire | None |
|  |  |  |  |  |  |  |  |  |  |  |  |  |  |  |  |  |
|  | Cohort studies - Anxiety or depression preceding | | | | | |  |  |  |  |  |  |  |  |  |  |
|  |  |  |  |  |  |  |  |  |  |  |  |  |  |  |  |  |
| **Ref** | **Year** | **Author** | **Country** | **Mean follow up (yrs)** | **Resource** | **Cohort** | **Male** | **Female** | **Cases** | **Male** | **Female** | **Mean Age** |  | **Definition** | **Exposure Assessment** | **Matching for** |
| 159 | 2003 | Weisskopf | US | 12 | Health Professionals Follow-Up Study | 35815 | 35815 | - | 189 | 189 | - | - |  | Neurologist diagnosed | Structured Questionnaire | Age, smoking, caffeine |
| 160 | 2006 | Brandt-Christensen | Denmark | 4.5 | Danish Civil Registration System | 1293789 | - | - | 11498 | - | - | - |  | Antiparkinson drug prescriptions | Antidepressant drug prescriptions | None |
|  |  |  |  |  |  |  |  |  |  |  |  |  |  |  |  |  |
|  |  |  |  |  |  |  |  |  |  |  |  |  |  |  |  |  |
|  | Case control study - Constipation preceding | | | | | |  |  |  |  |  |  |  |  |  |  |
|  |  |  |  |  |  |  |  |  |  |  |  |  |  |  |  |  |
| **Ref** | **Year** | **First Author** | **Country** | **Study period** | **Resource** | **Cases** | **Male** | **Female** | **Mean Age** | **Controls** | **Male** | **Female** | **Mean Age** | **Definition** | **Exposure assessment** | **Matching for** |
| 198 | 2009 | Savica | US | 1976-1995 | Rochester Epidemiology Project, Olmsted County, Minnesota | 196 | 121 | 75 | 71 | 196 | 121 | 75 | - | Medical record review (2 of 4 cardinal signs) | Medical record review | Smoking, coffee |
|  |  |  |  |  |  |  |  |  |  |  |  |  |  |  |  |  |
|  | Cohort study - Constipation preceding | | | | | |  |  |  |  |  |  |  |  |  |  |
|  |  |  |  |  |  |  |  |  |  |  |  |  |  |  |  |  |
| **Ref** | **Year** | **Author** | **Country** | **Mean follow up (yrs)** | **Resource** | **Cohort** | **Male** | **Female** | **Cases** | **Male** | **Female** | **Mean Age** |  | **Definition** | **Exposure Assessment** | **Matching for** |
| 199 | 2001 | Abbott | US | 12 | Honolulu Heart Program | 6790 | 6790 | - | 96 | 96 | - | - |  | Neurologist diagnosed | Structured Questionnaire | Age, smoking, coffee, laxative, jogging, fruit, vegetables, grains |
|  |  |  |  |  |  |  |  |  |  |  |  |  |  |  |  |  |
|  |  |  |  |  |  |  |  |  |  |  |  |  |  |  |  |  |
|  | Case control studies - Diabetes preceding | | | | | |  |  |  |  |  |  |  |  |  |  |
|  |  |  |  |  |  |  |  |  |  |  |  |  |  |  |  |  |
| **Ref** | **Year** | **First Author** | **Country** | **Study period** | **Resource** | **Cases** | **Male** | **Female** | **Mean Age** | **Controls** | **Male** | **Female** | **Mean Age** | **Definition** | **Exposure assessment** | **Matching for** |
| 97 | 1972 | Kessler | US | 1967-1969 | Private physician referrals in Baltimore area | 228 | 122 | 106 | - | 228 | 122 | 106 | - | Physician-diagnosed PD | Structured interview | None |
| 104 | 1989 | Ho | Hong Kong | - | 10 Old Age Homes in Shatin and Tai Po | 35 | 11 | 24 | - | 105 | - | - | - | Trained medical students using diagnostic criteria | Structured interview | None |
| 84 | 1994 | Morano | Spain | 1989-1990 | General Hospitals in Caceres, Spain | 74 | 33 | 41 | 68.2 | 148 | 66 | 82 | 67.5 | Diagnostic criteria for PD | Structured questionnaire | None |
| 62 | 2001 | Herishanu | Israel | 1989-1995 | PD clinic of Soroka University Medical Centre | 93 | - | - | - | 93 | - | - | - | Progressive disorder, 2 or more cardinal signs of PD | Interview with structured questionnaire | None |
| 129 | 2006 | Powers | US | 1992-2005 | Group Health Cooperative, Seattle area | 352 | 217 | 135 | 69 | 484 | 298 | 186 | 71 | Neurologist diagnosed or medical record review | Structured questionnaire | Age, ethnicity, education, smoking |
| 161 | 2008 | Becker | UK | 1994-2005 | General Practice Research Database, UK | 3637 | 2167 | 1470 | - | 3637 | 2167 | 1470 | - | Read-coded diagnosis of PD in database | Drug prescriptions in the database | BMI, smoking, comorbidities, diuretics, statins |
| 135 | 2009 | D'Amelio | Italy | - | Neurological Department, Palermo | 318 | 153 | 165 | 66.7 | 318 | 153 | 168 |  | 2 of 4 cardinal signs | Structured questionnaire | BMI, smoking, alcohol, coffee, education |
| 162 | 2009 | Rugbjerg | Denmark | 1986-2006 | Danish Hospital Register | 13695 | 7423 | 6272 | - | 68445 | 37101 | 31344 | - | ICD code for Parkinson's disease | Medical Record | None |
| 163 | 2010 | Miyake | Japan | 2006-2008 | 11 hospitals in Japan | 249 | 93 | 156 | 68.5 | 368 | 141 | 227 | 66.6 | Neurologist diagnosed (UK Brain Bank Criteria) | Structured questionnaire | Age, sex, region of residence, smoking, education, exercise, BMI, energy intake, cholesterol, vit E, alcohol, coffee and glycaemic index |
|  |  |  |  |  |  |  |  |  |  |  |  |  |  |  |  |  |
|  | Cohort studies - Diabetes preceding | | | | | |  |  |  |  |  |  |  |  |  |  |
|  |  |  |  |  |  |  |  |  |  |  |  |  |  |  |  |  |
| **Ref** | **Year** | **Author** | **Country** | **Mean follow up (yrs)** | **Resource** | **Cohort** | **Male** | **Female** | **Cases** | **Male** | **Female** | **Mean Age** |  | **Definition** | **Exposure Assessment** | **Matching for** |
| 38 | 1994 | Grandinetti | US | 26 | Honolulu Heart Program | 8006 | 8006 | - | 58 | 58 | - | - |  | Neurologist diagnosed | Structured questionnaire | Smoking, coffee, HTN, alcohol, cholesterol |
| 151 | 2007 | Hu | Finland | 12.9 | Population surveys | 29335 | 14293 | 15042 | 200 | 102 | 98 | - |  | Neurologist diagnosed | Structured questionnaire | Age, study year, BMI, BP, cholesterol, education, physical activity, smoking, alcohol, tea, coffee |
| 165 | 2007 | Simon | US | - | HPFS and NHS | 171879 | 50833 | 121046 | 530 | 266 | 264 | - |  | Neurologist diagnosed | Structured questionnaire | Age, smoking |
| 166 | 2008 | Driver | US | 23 | Physicians Health Study | 21841 | 21841 | - | 556 | 556 | - | - |  | Not specified | Structured questionnaire | Age, smoking |
|  |  |  |  |  |  |  |  |  |  |  |  |  |  |  |  |  |
|  |  |  |  |  |  |  |  |  |  |  |  |  |  |  |  |  |
|  | Case control studies - Cancer preceding | | | | | |  |  |  |  |  |  |  |  |  |  |
|  |  |  |  |  |  |  |  |  |  |  |  |  |  |  |  |  |
| **Ref** | **Year** | **First Author** | **Country** | **Study period** | **Resource** | **Cases** | **Male** | **Female** | **Mean Age** | **Controls** | **Male** | **Female** | **Mean Age** | **Definition** | **Exposure assessment** | **Matching for** |
| 97 | 1972 | Kessler | US | 1967-1969 | Private physician referrals in Baltimore area | 228 | 122 | 106 | - | 228 | 122 | 106 | - | Physician-diagnosed PD | Structured interview | None |
| 102 | 1987 | Rajput | US | 1967-1979 | Rochester Residents | 118 | 54 | 64 | - | 236 | 108 | 128 | - | Medical record review | Medical record review | None |
| 200 | 2002 | Elbaz | US | 1976-1995 | Rochester Epidemiology Project, Olmsted County, Minnesota | 196 | 121 | 75 | 71 | 196 | 121 | 75 | - | Medical record review (2 of 4 cardinal signs) | Medical record review | None |
| 201 | 2004 | D'Amelio | Italy | 2001-2002 | Neurological clinics in Palermo and Messina | 222 | 95 | 127 | 67.7 | 222 | 95 | 127 | - | 2 of 4 cardinal signs | Structured questionnaire | Smoking, alcohol, coffee |
| 202 | 2006 | Olsen | Denmark | 1977-1998 | Danish Hospital Register | 8090 | 4263 | 3827 | - | 32320 | 17012 | 15308 | - | ICD code for Parkinson's disease | Linked to Danish Cancer Registry | None |
| 129 | 2006 | Powers | US | 1992-2005 | Group Health Cooperative, Seattle area | 352 | 217 | 135 | 69 | 484 | 298 | 186 | 71 | Neurologist diagnosed or medical record review | Structured questionnaire | None |
| 203 | 2007 | Driver | US | 1982-2005 | Physicians Health Study | 487 | 487 | - | 60.6 | 487 | 487 | - | 60.6 | Medical record review | Structured questionnaire | Smoking, alcohol, BMI, exercise |
|  |  |  |  |  |  |  |  |  |  |  |  |  |  |  |  |  |
|  |  |  |  |  |  |  |  |  |  |  |  |  |  |  |  |  |
|  | Case control studies - Hypertension preceding | | | | | |  |  |  |  |  |  |  |  |  |  |
|  |  |  |  |  |  |  |  |  |  |  |  |  |  |  |  |  |
| **Ref** | **Year** | **First Author** | **Country** | **Study period** | **Resource** | **Cases** | **Male** | **Female** | **Mean Age** | **Controls** | **Male** | **Female** | **Mean Age** | **Definition** | **Exposure assessment** | **Matching for** |
| 97 | 1972 | Kessler | US | 1967-1969 | Private physician referrals in Baltimore area | 228 | 122 | 106 | - | 228 | 122 | 106 | - | Physician-diagnosed PD | Structured interview | None |
| 102 | 1987 | Rajput | US | 1967-1979 | Rochester Residents | 118 | 54 | 64 | - | 236 | 108 | 128 | - | - | Medical record review | None |
| 104 | 1989 | Ho | Hong Kong | - | 10 Old Age Homes in Shatin and Tai Po | 35 | 11 | 24 | - | 105 | - | - | - | Trained medical students using diagnostic criteria | Structured interview | None |
| 84 | 1994 | Morano | Spain | 1989-1990 | General Hospitals in Caceres, Spain | 74 | 33 | 41 | 68.2 | 148 | 66 | 82 | 67.5 | Diagnostic criteria for PD | Structured questionnaire | None |
| 71 | 1998 | McCann | Australia | - | Clinics and Residential Care Centres, Queensland and New South Wales | 224 | 131 | 93 | 70.3 | 310 | 193 | 117 | 68.9 | Calne criteria | Questionnaire | Age, sex, rural residency, well water, FHx, stroke |
| 62 | 2001 | Herishanu | Israel | 1989-1995 | PD clinic of Soroka University Medical Centre | 93 | - | - | - | 93 | - | - | - | Progressive disorder, 2 or more cardinal signs of PD | Interview with structured questionnaire | None |
| 122 | 2001 | Paganini-Hill | US | 1981-1998 | Leisure World Cohort, California | 395 | - | - | 75 | 2320 | - | - | 75 | Hospital record and death certificate | Structured questionnaire | None |
| 129 | 2006 | Powers | US | 1992-2005 | Group Health Cooperative, Seattle area | 352 | 217 | 135 | 69 | 484 | 298 | 186 | 71 | Neurologist diagnosed or medical record review | Structured questionnaire | None |
| 161 | 2008 | Becker | UK | 1994-2005 | General Practice Research Database, UK | 3637 | 2167 | 1470 | - | 3637 | 2167 | 1470 | - | Read-coded diagnosis of PD in database | Drug prescriptions in the database | Age, sex, BMI, smoking, comorbidities |
| 163 | 2010 | Miyake | Japan | 2006-2008 | 11 hospitals in Japan | 249 | 93 | 156 | 68.5 | 368 | 141 | 227 | 66.6 | Neurologist diagnosed (UK Brain Bank Criteria) | Structured questionnaire | Age, sex, region of residence, smoking, education, exercise, BMI, energy intake, cholesterol, vitamin E, alcohol, coffee and glycaemic index |
|  |  |  |  |  |  |  |  |  |  |  |  |  |  |  |  |  |
|  | Cohort studies - Hypertension preceding | | | | | |  |  |  |  |  |  |  |  |  |  |
|  |  |  |  |  |  |  |  |  |  |  |  |  |  |  |  |  |
| **Ref** | **Year** | **Author** | **Country** | **Mean follow up (yrs)** | **Resource** | **Cohort** | **Male** | **Female** | **Cases** | **Male** | **Female** | **Mean Age** |  | **Definition** | **Exposure Assessment** | **Matching for** |
| 38 | 1994 | Grandinetti | US | 26 | Honolulu Heart Program | 8006 | 8006 | - | 58 | 58 | - | - |  | Neurologist diagnosed | Structured questionnaire | Smoking, coffee, diabetes, alcohol, cholesterol |
| 165 | 2007 | Simon | US | - | HPFS and NHS | 171879 | 50833 | 121046 | 530 | 266 | 264 | - |  | Neurologist diagnosed | Structured questionnaire | Age, smoking |
|  |  |  |  |  |  |  |  |  |  |  |  |  |  |  |  |  |
|  |  |  |  |  |  |  |  |  |  |  |  |  |  |  |  |  |
|  | Case control studies - Gastric ulcer preceding | | | | | |  |  |  |  |  |  |  |  |  |  |
|  |  |  |  |  |  |  |  |  |  |  |  |  |  |  |  |  |
| **Ref** | **Year** | **First Author** | **Country** | **Study period** | **Resource** | **Cases** | **Male** | **Female** | **Mean Age** | **Controls** | **Male** | **Female** | **Mean Age** | **Definition** | **Exposure assessment** | **Matching for** |
| 97 | 1972 | Kessler | US | 1967-1969 | Private physician referrals in Baltimore area | 228 | 122 | 106 | - | 228 | 122 | 106 | - | Physician-diagnosed PD | Structured interview | None |
| 62 | 2001 | Herishanu | Israel | 1989-1995 | PD clinic of Soroka University Medical Centre | 93 | - | - | - | 93 | - | - | - | Progressive disorder, 2 or more cardinal signs of PD | Interview with structured questionnaire | None |
| 128 | 2006 | Ma | China | 1985-2000 | Linxian, Henan Province | 85 | 40 | 45 | - | 340 | 160 | 180 | - | Neurologist diagnosed (UK Brain Bank Criteria) | Structured questionnaire | BMI, meat consumption, smoking |
|  |  |  |  |  |  |  |  |  |  |  |  |  |  |  |  |  |
|  |  |  |  |  |  |  |  |  |  |  |  |  |  |  |  |  |
|  | Case control studies - Prior general anaesthetic | | | | | |  |  |  |  |  |  |  |  |  |  |
|  |  |  |  |  |  |  |  |  |  |  |  |  |  |  |  |  |
| **Ref** | **Year** | **First Author** | **Country** | **Study period** | **Resource** | **Cases** | **Male** | **Female** | **Mean Age** | **Controls** | **Male** | **Female** | **Mean Age** | **Definition** | **Exposure assessment** | **Matching for** |
| 105 | 1989 | Hofman | Holland | 1981-1986 | General Practices in Netherlands | 86 | - | - | - | 172 | - | - | - | Neurologist diagnosed | Structured questionnaire | Age, gender |
| 87 | 1996 | de Michele | Italy | - | Department of Neurology Federico II University in Naples | 116 | 77 | 39 | 62.5 | 232 | 116 | 116 | 62.4 | 2 of 3 cardinal signs of PD | Structured questionnaire | None |
| 88 | 1996 | Seidler | Germany | - | Nine Neurology clinics across Germany | 380 | 251 | 129 | 56.2 | 359 | - | - | 56.5 | Neurologist diagnosed (UK Brain Bank Criteria) | Structured interview | Smoking, education |
| 118 | 1998 | Smargiassi | Italy | - | Emilia-Romagna Region, Italy | 86 | 50 | 36 | 66.4 | 86 | 48 | 38 | 63.1 | Neurologist diagnosed (UK Brain Bank Criteria) | Structured questionnaire | None |
| 82 | 2002 | Zorzon | Italy | 1998 | Center for Parkinson's disease and Movement Disorders, Trieste | 136 | 62 | 74 | 70 | 272 | 124 | 148 | 69 | Neurologist diagnosed PD (2 of 4 cardinal signs) | Structured interview | Smoking, FHx of PD or ET, maternal age, farming, well water |
| 59 | 2007 | Dick | UK | 2000-2004 | GEOPARKINSON study | 767 | 426 | 341 | 69.8 | 1989 | 1057 | 932 | 69.8 | Physician/medical record (UK Brain Bank criteria) | Interview | Age, sex, country, tobacco use, ever knocked unconscious, FHx |
|  |  |  |  |  |  |  |  |  |  |  |  |  |  |  |  |  |
|  |  |  |  |  |  |  |  |  |  |  |  |  |  |  |  |  |
|  | Case control study - Head injury preceding | | | | | |  |  |  |  |  |  |  |  |  |  |
|  |  |  |  |  |  |  |  |  |  |  |  |  |  |  |  |  |
| **Ref** | **Year** | **Author** | **Country** | **Study period** | **Resource** | **Cases** | **Male** | **Female** | **Mean Age** | **Control** | **Male** | **Female** | **Mean Age** | **Definition** | **Exposure Assessment** | **Matching for** |
| 97 | 1972 | Kessler | US | 1967-1969 | Private physician referrals in Baltimore area | 228 | 122 | 106 | - | 228 | 122 | 106 | - | Physician-diagnosed PD | Structured interview | None |
| 103 | 1987 | Tanner | China | - | Beijing and Guangzhou, Peoples Republic of China | 100 | 77 | 23 | 57.2 | 200 | 154 | 46 | 56.8 | Neurologist diagnosed | Structured interview | None |
| 105 | 1989 | Hofman | Holland | 1981-1986 | Sentinel Practices of the Netherlands Institute for General Practice | 86 | - | - | - | 172 | - | - | - | Neurologist diagnosed | Structured questionnaire | Age, gender |
| 167 | 1991 | Factor | US | - | Movement Disorder Clinic, University of Miami and Albany Medical College | 97 | 58 | 39 | 68.6 | 64 | 23 | 41 | 63 | Neurologist diagnosis (2 of 4 cardinal signs) | Structured questionnaire | None |
| 113 | 1995 | Martyn | UK | - | 42 General Practices, Hertfordshire | 172 | 108 | 64 | 70.8 | 343 | 220 | 123 | 70.7 | Neurologist or geriatrician diagnosed PD | Structured questionnaire | None |
| 168 | 1995 | Semchuk | Canada | 1989 | Calgary Residents | 130 | 75 | 55 | 68.5 | 260 | 150 | 110 | 68.3 | Neurologist diagnosed PD | Structured interview | FHx, herbicides |
| 87 | 1996 | de Michele | Italy | - | Department of Neurology Federico II University in Naples | 116 | 77 | 39 | 62.5 | 232 | 116 | 116 | 62.4 | 2 of 3 cardinal signs of PD | Structured questionnaire | None |
| 71 | 1998 | McCann | Australia | - | Clinics and Residential Care Centres, Queensland and New South Wales | 224 | 131 | 93 | 70.3 | 310 | 193 | 117 | 68.9 | Calne criteria | Questionnaire | None |
| 118 | 1998 | Smargiassi | Italy | - | Emilia-Romagna Region, Italy | 86 | 50 | 36 | 66.4 | 86 | 48 | 38 | 63.1 | Neurologist diagnosed (UK Brain Bank Criteria) | Structured questionnaire | None |
| 120 | 1999 | Kuopio | Finland | 1994-1996 | Nine rural municipalities in Finland | 123 | 63 | 60 | 68.7 | 246 | 126 | 120 | 69.3 | Neurologist diagnosed | Structured interview | None |
| 81 | 1999 | Taylor | US | - | Movement Disorder Centre at Boston Medical Centre | 140 | 88 | 52 | 66.2 | 147 | 90 | 57 | 66.9 | Neurologist diagnosed (UK Brain Bank Criteria) | Structured interview | Birth cohort, sex, FHx, depression, education, urban/suburban/rural living, pesticides, herbicides, well water, smoking, vitamins |
| 82 | 2002 | Zorzon | Italy | 1998 | Center for Parkinson's disease and Movement Disorders, Trieste | 136 | 62 | 74 | 70 | 272 | 124 | 148 | 69 | Neurologist diagnosed (2 of 4 cardinal signs) | Structured interview | Smoking |
| 60 | 2003 | Duzcan | Turkey | 2000 | Kizilcaboluk-Denizli | 36 | 17 | 19 | - | 108 | 51 | 57 | - | Neurologist diagnosed Parkinsonism (2 of 4 cardinal signs) | Questionnaire | None |
| 123 | 2003 | Baldereschi | Italy | 1992-1993 | Italian Longitudinal Study on Aging | 113 | 58 | 57 | 78.1 | 4383 | 2247 | 2136 | 74.5 | Medical record (2 of 4 cardinal signs) | Structured questionnaire | Age, gender, years of schooling, smoking, pesticide use license |
| 169 | 2003 | Bower | US | 1976-1995 | Rochester Epidemiology Project, Olmsted County, Minnesota | 196 | 121 | 75 | 71 | 196 | 121 | 75 | - | Medical record review (2 of 4 cardinal signs) | Medical record review | None |
| 59 | 2007 | Dick | UK | 2000-2004 | GEOPARKINSON study | 767 | 426 | 341 | 69.8 | 1989 | 1057 | 932 | 69.8 | Physician/medical record (UK Brain Bank criteria) | Interview | Age, sex, country, tobacco use, FHx |
| 170 | 2008 | Rugbjerg | Denmark | 1986-2006 | Danish National Hospital Register | 13695 | 7423 | 6272 | 73 | 68445 | 37101 | 31344 | - | ICD codes for PD in hospital register | ICD codes for hospital contact with head injury | None |
| 137 | 2009 | Tanner | US | 2004-2007 | Movement Disorder Centres in North America | 519 | 309 | 210 | 65 | 511 | 302 | 209 | 65 | 2 of 4 cardinal signs | Structured interview | None |
| 93 | 2010 | Sanyal | India | - | Movement Disorders clinic in Kolkata | 175 | 140 | 35 | 55.2 | 350 | 280 | 70 | 55 | 3 of 4 cardinal signs of PD | Structured interview | None |
|  |  |  |  |  |  |  |  |  |  |  |  |  |  |  |  |  |
|  | Case control studies - NSAIDs use | | | | | |  |  |  |  |  |  |  |  |  |  |
|  |  |  |  |  |  |  |  |  |  |  |  |  |  |  |  |  |
| **Ref** | **Year** | **Author** | **Country** | **Study period** | **Resource** | **Cases** | **Male** | **Female** | **Mean Age** | **Control** | **Male** | **Female** | **Mean Age** | **Definition** | **Exposure Assessment** |  |
| 223 | 2006 | Bower | US | 1976-1995 | Rochester Epidemiology Project, Olmsted County, Minnesota | 196 | 121 | 75 | 71 | 196 | 121 | 75 | - | Medical record review (2 of 4 cardinal signs) | Medical record review |  |
| 226 | 2006 | Ton | US | 1992-2002 | Group Health Cooperative, Seattle area | 206 | 121 | 85 | 69.2 | 383 | 239 | 144 | 69.4 | 2 of 4 cardinal signs | GHC pharmacy database |  |
| 227 | 2006 | Hernan | UK | 1995-2001 | General Practice Research Database | 1258 | 765 | 493 | 70.7 | 6638 | 3923 | 2715 | 68.7 | Computerised record review | Computer record review |  |
| 225 | 2007 | Wahner | US | 2001-2006 | 3 rural Californian counties (Fresno, Tulare, Kern) | 293 | 157 | 136 | 70 | 286 | 146 | 140 | 69 | Neurologist diagnosed PD (2 of 3 cardinal signs) | Structured questionnaire |  |
| 134 | 2008 | Powers | US | 1996-2005 | NeuroGenetics Research Consortium (NGRC) | 1186 | 790 | 396 | 69.6 | 928 | 374 | 554 | 70.6 | Neurologist diagnosed PD | Structured questionnaire |  |
|  |  |  |  |  |  |  |  |  |  |  |  |  |  |  |  |  |
|  | Cohort studies - NSAIDs use | | | | | |  |  |  |  |  |  |  |  |  |  |
|  |  |  |  |  |  |  |  |  |  |  |  |  |  |  |  |  |
| **Ref** | **Year** | **Author** | **Country** | **Mean follow up (yrs)** | **Resource** | **Cohort** | **Male** | **Female** | **Cases** | **Male** | **Female** | **Mean Age** |  | **Definition** | **Exposure Assessment** | **Matching for** |
| 228 | 2003 | Chen | US | - | HPFS and NHS | 142902 | 44057 | 98845 | 414 | 236 | 178 | - |  | Neurologist diagnosed (2 or 3 cardinal signs) | Structured questionnaire | Age, smoking, caffeine, alcohol |
| 229 | 2005 | Chen | US | - | Cancer Prevention Study II Nutrition Cohort | 146948 | 65657 | 81291 | 413 | - | - | - |  | Neurologist diagnosed | Structured questionnaire | Age, sex, smoking |
| 230 | 2007 | Bornebroek | Netherlands | - | The Rotterdam Study | 6512 | 2667 | 3845 | 88 | - | - | - |  | Physician diagnosed | Pharmacy databases | Age, sex, smoking, coffee |
| 231 | 2008 | Etminan | US | - | British Columbia Linked Health Database | 697078 | - | - | 5010 | - | - | - |  | Two prescriptions for PD medication | British Columbia Linked Health Database | None |
|  |  |  |  |  |  |  |  |  |  |  |  |  |  |  |  |  |
|  |  |  |  |  |  |  |  |  |  |  |  |  |  |  |  |  |
|  | Case control studies - Aspirin use | | | | | |  |  |  |  |  |  |  |  |  |  |
|  |  |  |  |  |  |  |  |  |  |  |  |  |  |  |  |  |
| **Ref** | **Year** | **First Author** | **Country** | **Study period** | **Resource** | **Cases** | **Male** | **Female** | **Mean Age** | **Controls** | **Male** | **Female** | **Mean Age** | **Definition** | **Exposure assessment** | **Matching for** |
| 223 | 2006 | Bower | US | 1976-1995 | Rochester Epidemiology Project, Olmsted County, Minnesota | 196 | 121 | 75 | 71 | 196 | 121 | 75 | - | Medical record review (2 of 4 cardinal signs) | Medical record review | None |
| 227 | 2006 | Hernan | UK | 1995-2001 | General Practice Research Database | 1258 | 765 | 493 | 70.7 | 6638 | 3923 | 2715 | 68.7 | Computerised record review | Computer record review | None |
| 226 | 2006 | Ton | US | 1992-2002 | Group Health Cooperative, Seattle area | 206 | 121 | 85 | 69.2 | 383 | 239 | 144 | 69.4 | 2 of 4 cardinal signs | GHC pharmacy database | Age, sex, smoking, duration of enrolment, clinic |
| 225 | 2007 | Wahner | US | 2001-2006 | 3 rural Californian counties (Fresno, Tulare, Kern) | 293 | 157 | 136 | 70 | 286 | 146 | 140 | 69 | Neurologist diagnosed PD (2 of 3 cardinal signs) | Structured questionnaire | Gender, race, age at diagnosis, smoking, education, other NSAID use |
|  |  |  |  |  |  |  |  |  |  |  |  |  |  |  |  |  |
|  | Cohort studies - Aspirin use | | | | | |  |  |  |  |  |  |  |  |  |  |
|  |  |  |  |  |  |  |  |  |  |  |  |  |  |  |  |  |
| **Ref** | **Year** | **Author** | **Country** | **Mean follow up (yrs)** | **Resource** | **Cohort** | **Male** | **Female** | **Cases** | **Male** | **Female** | **Mean Age** |  | **Definition** | **Exposure Assessment** | **Matching for** |
| 228 | 2003 | Chen | US | - | HPFS & NHS | 142902 | 44057 | 98845 | 415 | 236 | 179 | - |  | Neurologist Diagnosed | Structured questionnaire | Smoking, coffee, alcohol |
| 229 | 2005 | Chen | US | - | Cancer Prevention Study II Nutrition Cohort | 146948 | 65657 | 81291 | 413 | - | - | - |  | Neurologist Diagnosed + Medical Record | Structured questionnaire | Age, sex, smoking |
|  |  |  |  |  |  |  |  |  |  |  |  |  |  |  |  |  |
|  |  |  |  |  |  |  |  |  |  |  |  |  |  |  |  |  |
|  | Case control study - Acetaminophen/Paracetamol use | | | | | |  |  |  |  |  |  |  |  |  |  |
|  |  |  |  |  |  |  |  |  |  |  |  |  |  |  |  |  |
| **Ref** | **Year** | **First Author** | **Country** | **Study period** | **Resource** | **Cases** | **Male** | **Female** | **Mean Age** | **Controls** | **Male** | **Female** | **Mean Age** | **Definition** | **Exposure assessment** | **Matching for** |
| 227 | 2006 | Hernan | UK | 1995-2001 | General Practice Research Database | 1258 | 765 | 493 | 70.7 | 6638 | 3923 | 2715 | 68.7 | Computerised record review | Computer record review | None |
|  |  |  |  |  |  |  |  |  |  |  |  |  |  |  |  |  |
|  | Cohort study - Acetaminophen/Paracetamol use | | | | | |  |  |  |  |  |  |  |  |  |  |
|  |  |  |  |  |  |  |  |  |  |  |  |  |  |  |  |  |
| **Ref** | **Year** | **Author** | **Country** | **Mean follow up (yrs)** | **Resource** | **Cohort** | **Male** | **Female** | **Cases** | **Male** | **Female** | **Mean Age** |  | **Definition** | **Exposure Assessment** | **Matching for** |
| 229 | 2005 | Chen | US | - | Cancer Prevention Study II Nutrition Cohort | 146948 | 65657 | 81291 | 413 | - | - | - |  | Neurologist Diagnosed + Medical Record | Structured questionnaire | Age, sex, smoking |
|  |  |  |  |  |  |  |  |  |  |  |  |  |  |  |  |  |
|  |  |  |  |  |  |  |  |  |  |  |  |  |  |  |  |  |
|  | Case control study - Statin use | | | | | |  |  |  |  |  |  |  |  |  |  |
|  |  |  |  |  |  |  |  |  |  |  |  |  |  |  |  |  |
| **Ref** | **Year** | **Author** | **Country** | **Study period** | **Resource** | **Cases** | **Male** | **Female** | **Mean Age** | **Control** | **Male** | **Female** | **Mean Age** | **Definition** | **Exposure Assessment** | **Matching for** |
| 212 | 2007 | Huang | US | 2002-2004 | Movement Disorder Clinic at University of North Carolina | 124 | 69 | 55 | 67.9 | 112 | 50 | 62 | 65.7 | Published criteria for PD | Structured interview | Age, gender, smoking |
| 234 | 2008 | Wahner | US | 2001-2007 | UCLA PEG Study | 312 | 166 | 146 | 70 | 342 | 168 | 174 | 69 | ICD codes for PD in hospital register | Questionnaire | Age, sex, smoking, race, education, county |
| 232 | 2008 | Samii | Canada | 1997-2003 | British Columbia Linked Health Databases | 4756 | 2678 | 2078 | 73.2 | 19024 | 7800 | 11224 | 72.4 | Physician diagnosis and at least 2 prescriptions for PD medication | Database screened for prescription drugs (statins) | None |
| 49 | 2008 | Becker | UK | 1994-2005 | General Practice Research Database, UK | 3637 | 2167 | 1470 | - | 3637 | 2167 | 1470 | - | Read-coded diagnosis of PD in database | Drug prescriptions in the database | None |
| 233 | 2010 | Ritz | Denmark | 2001-2006 | Danish Hospital Register | 1931 | 1121 | 810 | 72.2 | 9651 | 5603 | 4048 | 72.2 | Danish hospital register, diagnosed by ICD classification | National prescriptions database for statin prescriptions | Age, sex, COPD, Charlson index |
|  |  |  |  |  |  |  |  |  |  |  |  |  |  |  |  |  |
|  |  |  |  |  |  |  |  |  |  |  |  |  |  |  |  |  |
|  | Case control studies - Hormone replacement therapy | | | | | |  |  |  |  |  |  |  |  |  |  |
|  |  |  |  |  |  |  |  |  |  |  |  |  |  |  |  |  |
| **Ref** | **Year** | **First Author** | **Country** | **Study period** | **Resource** | **Cases** | **Male** | **Female** | **Mean Age** | **Controls** | **Male** | **Female** | **Mean Age** | **Definition** | **Exposure assessment** | **Matching for** |
| 235 | 1998 | Marder | US | - | Washington Heights-Inwood, New York | 87 | - | 87 | 71.2 | 989 | - | 989 | 74.2 | Published research criteria | - | Age, education, ethnicity |
| 236 | 2001 | Benedetti | US | 1976-1995 | Rochester Epidemiology Project, Olmsted County, Minnesota | 72 | - | 72 |  | 72 | - | 72 |  | Medical record review (2 of 4 cardinal signs) | Medical record review | Age of menopause, education |
| 237 | 2003 | Martignoni | Italy | - | Movement Disorders clinics in Pavia and Varese | 150 | - | 150 | 65.5 | 300 | - | 300 | 63.5 | Neurologist confirmed | Structured interview | None |
| 126 | 2003 | Pals | Belgium | - | Patient support groups and three Flemish universities | 423 | 256 | 167 | 67 | 205 | 70 | 135 | - | Neurologist confirmed (3 of 4 cardinal signs) | Structured questionnaire | Age, FHx |
| 238 | 2004 | Currie | US | 1999 | Movement Disorders clinic at the University of Virginia | 68 | - | 68 | 71 | 72 | - | 72 | 66 | 2 of 3 cardinal signs | Structured interview | Age at menarche, age at menopause, menses duration, number of pregnancies, OCP use and duration. |
| 239 | 2004 | Ragonese | Italy | - | Neurological clinics in Palermo and Messina | 131 | - | 131 | 68.7 | 131 | - | 131 | 68.7 | 2 of 4 cardinal signs | Structured questionnaire | Education, smoking, alcohol, coffee |
| 240 | 2005 | Popat | US | 1994-1995 | Northern California Kaiser Permanente Medical Care Program | 178 | - | 178 | 71.3 | 189 | - | 189 | 70.7 | Neurologist confirmed (medical record review) | Structured interview | Age, respondent type, smoking, age at final menstrual period, type of menopause |
|  |  |  |  |  |  |  |  |  |  |  |  |  |  |  |  |  |
|  | Cohort studies - Hormone replacement therapy | | | | | |  |  |  |  |  |  |  |  |  |  |
|  |  |  |  |  |  |  |  |  |  |  |  |  |  |  |  |  |
| **Ref** | **Year** | **Author** | **Country** | **Mean follow up (yrs)** | **Resource** | **Cohort** | **Male** | **Female** | **Cases** | **Male** | **Female** | **Mean Age** |  | **Definition** | **Exposure Assessment** | **Matching for** |
| 150 | 2004 | Ascherio | US | - | Cancer Prevention Study II | 539222 | 301164 | 238058 | 1249 | 909 | 340 | - |  | Death certificates including PD as diagnosis | Structured questionnaire | Age, smoking alcohol coffee, OCP, age at menopause, type of menopause, parity |
| 241 | 2009 | Simon | US | - | NHS | 121701 | - | 121701 | 244 | - | 244 | - |  | Neurologist diagnosed | Structured questionnaire | Smoking |
|  |  |  |  |  |  |  |  |  |  |  |  |  |  |  |  |  |
|  |  |  |  |  |  |  |  |  |  |  |  |  |  |  |  |  |
|  | Case control studies - Oophorectomy preceding | | | | | |  |  |  |  |  |  |  |  |  |  |
|  |  |  |  |  |  |  |  |  |  |  |  |  |  |  |  |  |
| **Ref** | **Year** | **First Author** | **Country** | **Study period** | **Resource** | **Cases** | **Male** | **Female** | **Mean Age** | **Controls** | **Male** | **Female** | **Mean Age** | **Definition** | **Exposure assessment** | **Matching for** |
| 236 | 2001 | Benedetti | US | 1976-1995 | Rochester Epidemiology Project, Olmsted County, Minnesota | 72 | - | 72 |  | 72 | - | 72 | - | Medical record review (2 of 4 cardinal signs) | Medical record review | HRT |
| 237 | 2003 | Martignoni | Italy | - | Movement Disorders clinics in Pavia and Varese | 150 | - | 150 | 65.5 | 300 | - | 300 | 63.5 | Neurologist confirmed | Structured interview | None |
| 239 | 2004 | Ragonese | Italy | - | Neurological clinics in Palermo and Messina | 131 | - | 131 | 68.7 | 131 | - | 131 | 68.7 | 2 of 4 cardinal signs | Structured questionnaire | Education, smoking, alcohol, coffee |
| 240 | 2005 | Popat | US | 1994-1995 | Northern California Kaiser Permanente Medical Care Program | 178 | - | 178 | 71.3 | 189 | - | 189 | 70.7 | Neurologist confirmed (medical record review) | Structured interview | Age, respondent type, smoking |
|  |  |  |  |  |  |  |  |  |  |  |  |  |  |  |  |  |
|  | Cohort study - Oophorectomy preceding | | | | | |  |  |  |  |  |  |  |  |  |  |
|  |  |  |  |  |  |  |  |  |  |  |  |  |  |  |  |  |
| **Ref** | **Year** | **Author** | **Country** | **Mean follow up (yrs)** | **Resource** | **Cohort** | **Male** | **Female** | **Cases** | **Male** | **Female** | **Mean Age** |  | **Definition** | **Exposure Assessment** | **Matching for** |
| 241 | 2009 | Simon | US | - | NHS | 121701 | - | 121701 | 244 | - | 244 | - |  | Neurologist diagnosed | Structured questionnaire | Smoking |
|  |  |  |  |  |  |  |  |  |  |  |  |  |  |  |  |  |
|  |  |  |  |  |  |  |  |  |  |  |  |  |  |  |  |  |
|  | Case control studies - Oral contraceptive pill use | | | | | |  |  |  |  |  |  |  |  |  |  |
|  |  |  |  |  |  |  |  |  |  |  |  |  |  |  |  |  |
| **Ref** | **Year** | **First Author** | **Country** | **Study period** | **Resource** | **Cases** | **Male** | **Female** | **Mean Age** | **Controls** | **Male** | **Female** | **Mean Age** | **Definition** | **Exposure assessment** | **Matching for** |
| 237 | 2003 | Martignoni | Italy | - | Movement Disorders clinics in Pavia and Varese | 150 | - | 150 | 65.5 | 300 | - | 300 | 63.5 | Neurologist confirmed | Structured interview | None |
| 240 | 2005 | Popat | US | 1994-1995 | Northern California Kaiser Permanente Medical Care Program | 178 | - | 178 | 71.3 | 189 | - | 189 | 70.7 | Neurologist confirmed (medical record review) | Structured interview | None |
|  |  |  |  |  |  |  |  |  |  |  |  |  |  |  |  |  |
|  | Cohort study - Oral contraceptive pill use | | | | | |  |  |  |  |  |  |  |  |  |  |
|  |  |  |  |  |  |  |  |  |  |  |  |  |  |  |  |  |
| **Ref** | **Year** | **Author** | **Country** | **Mean follow up (yrs)** | **Resource** | **Cohort** | **Male** | **Female** | **Cases** | **Male** | **Female** | **Mean Age** |  | **Definition** | **Exposure Assessment** | **Matching for** |
| 241 | 2009 | Simon | US | - | NHS | 121701 | - | 121701 | 244 | - | 244 | - |  | Neurologist diagnosed | Structured questionnaire | Smoking |
|  |  |  |  |  |  |  |  |  |  |  |  |  |  |  |  |  |
|  |  |  |  |  |  |  |  |  |  |  |  |  |  |  |  |  |
|  | Case control studies - Calcium channel blocker use | | | | | |  |  |  |  |  |  |  |  |  |  |
|  |  |  |  |  |  |  |  |  |  |  |  |  |  |  |  |  |
| **Ref** | **Year** | **Author** | **Country** | **Study period** | **Resource** | **Cases** | **Male** | **Female** | **Mean Age** | **Control** | **Male** | **Female** | **Mean Age** | **Definition** | **Exposure Assessment** | **Matching for** |
| 242 | 2007 | Ton | US | 1992-2002 | Group Health Cooperative, Seattle area | 206 | 121 | 85 | 69.2 | 383 | 239 | 144 | 69.4 | 2 of 4 cardinal signs | GHC pharmacy database | Age, sex, smoking, duration of enrolment, clinic, beta-blockers |
| 161 | 2008 | Becker | UK | 1994-2005 | General Practice Research Database, UK | 3637 | 2167 | 1470 | - | 3637 | 2167 | 1470 | - | Read-coded diagnosis of PD in database | Drug prescriptions in the database | BMI, smoking, comorbidities, diuretics, statins |
| 243 | 2009 | Louis | Spain | 1994-1998 | Neurological Disorders in Central Spain Study (NEDICES) | 81 | 43 | 38 | 77 | 4663 | 2001 | 2662 | 73.7 | Neurologist diagnoses (2 of 4 cardinal signs) | Structured questionnaire | Age, gender, education, depression |
| 244 | 2010 | Ritz | Denmark | 2001-2006 | Danish Hospital Register | 1931 | 1121 | 810 | 72.2 | 9651 | 5603 | 4048 | 72.2 | Danish hospital register, diagnosed by ICD classification | National prescriptions database for statin prescriptions | Age, sex, COPD, Charlson index |
|  |  |  |  |  |  |  |  |  |  |  |  |  |  |  |  |  |
|  | Cohort studies - Calcium channel blocker use | | | | | |  |  |  |  |  |  |  |  |  |  |
|  |  |  |  |  |  |  |  |  |  |  |  |  |  |  |  |  |
| **Ref** | **Year** | **Author** | **Country** | **Mean follow up (yrs)** | **Resource** | **Cohort** | **Male** | **Female** | **Cases** | **Male** | **Female** | **Mean Age** |  | **Definition** | **Exposure Assessment** | **Matching for** |
| 245 | 2010 | Simon | US | - | HPFS & NHS | 171355 | 50825 | 120530 | 514 | 324 | 190 | - |  | Neurologist Diagnosed | Structured questionnaire | Age, smoking, hypertension, caffeine, alcohol, BMI, physical activity, total calorie intake |
|  |  |  |  |  |  |  |  |  |  |  |  |  |  |  |  |  |
|  |  |  |  |  |  |  |  |  |  |  |  |  |  |  |  |  |
|  | Case control studies - Beta blocker use | | | | | |  |  |  |  |  |  |  |  |  |  |
|  |  |  |  |  |  |  |  |  |  |  |  |  |  |  |  |  |
| **Ref** | **Year** | **First Author** | **Country** | **Study period** | **Resource** | **Cases** | **Male** | **Female** | **Mean Age** | **Controls** | **Male** | **Female** | **Mean Age** | **Definition** | **Exposure assessment** | **Matching for** |
| 242 | 2007 | Ton | US | 1992-2002 | Group Health Cooperative, Seattle area | 206 | 121 | 85 | 69.2 | 383 | 239 | 144 | 69.4 | 2 of 4 cardinal signs | GHC pharmacy database | Age, sex, smoking, duration of enrolment, clinic |
| 161 | 2008 | Becker | UK | 1994-2005 | General Practice Research Database, UK | 3637 | 2167 | 1470 | - | 3637 | 2167 | 1470 | - | Read-coded diagnosis of PD in database | Drug prescriptions in the database | None |
| 244 | 2010 | Ritz | Denmark | 2001-2006 | Danish Hospital Register | 1931 | 1121 | 810 | 72.2 | 9651 | 5603 | 4048 | 72.2 | Danish hospital register, diagnosed by ICD classification | National prescriptions database for statin prescriptions | Age, sex, COPD, Charlson index, other anti-hypertensives |
|  |  |  |  |  |  |  |  |  |  |  |  |  |  |  |  |  |
|  |  |  |  |  |  |  |  |  |  |  |  |  |  |  |  |  |
|  | Case control studies - Pesticide exposure | | | | | |  |  |  |  |  |  |  |  |  |  |
|  |  |  |  |  |  |  |  |  |  |  |  |  |  |  |  |  |
| **Ref** | **Year** | **First Author** | **Country** | **Study period** | **Resource** | **Cases** | **Male** | **Female** | **Mean Age** | **Controls** | **Male** | **Female** | **Mean Age** | **Definition** | **Exposure assessment** | **Matching for** |
| 104 | 1989 | Ho | Hong Kong | - | 10 Old Age Homes in Shatin and Tai Po | 35 | 11 | 24 | - | 105 | - | - | - | Trained medical students using diagnostic criteria | Structured interview | None |
| 171 | 1990 | Golbe | US | 1986-1988 | Movement disorder centre at UMNDNJ-Robert Wood Johnson Medical School | 106 | 71 | 35 | - | 106 | 35 | 71 | - | Neurologist diagnosed PD | Structured questionnaire | None |
| 172 | 1990 | Koller | US | - | Movement Disorder clinic, University of Kansas Medical Centre | 150 | 89 | 61 | 66 | 150 | 89 | 61 | 66.3 | Neurologist diagnosed PD (2 of 4 cardinal signs) | Structured interview | None |
| 111 | 1992 | Jimenez-Jimenez | Spain | - | Hospital General Gregario Maranon, Madrid | 128 | 67 | 61 | 66.8 | 256 | 134 | 122 | 64.8 | Not specified | Structured questionnaire | None |
| 155 | 1993 | Hubble | US | - | Hayes and Kansas City | 63 | 34 | 29 | - | 75 | 34 | 41 | - | Neurologist diagnosed (2 of 4 cardinal signs) | Structured questionnaire | Neurologic family history, depression |
| 173 | 1994 | Hertzman | Canada | - | Okanagan Valley, British Columbia | 127 | 71 | 56 | 70.5 | 124 | 60 | 64 | 69.6 | Neurologist diagnosed PD (2 of 4 cardinal signs) | Structured interview | None |
| 84 | 1994 | Morano | Spain | 1989-1990 | General Hospitals in Caceres, Spain | 74 | 33 | 41 | 68.2 | 148 | 66 | 82 | 67.5 | Diagnostic criteria for PD | Structured questionnaire | None |
| 174 | 1995 | Chaturvedi | Canada | 1991-1992 | Canadian Study of Health and Aging | 87 | - | - | - | 2070 | - | - | - | No details given | Structured questionnaire | None |
| 168 | 1995 | Semchuk | Canada | 1989 | Calgary Residents | 130 | 75 | 55 | 68.5 | 260 | 150 | 110 | 68.3 | Neurologist diagnosed PD | Structured interview | FHx, head injury |
| 88 | 1996 | Seidler | Germany | - | Nine Neurology clinics across Germany | 380 | 251 | 129 | 56.2 | 359 | - | - | 56.5 | Neurologist diagnosed (UK Brain Bank Criteria) | Structured interview | Smoking, education |
| 115 | 1997 | Liou | China | 1993-1995 | Movement Disorder clinic, National Taiwan University Hospital | 120 | 65 | 55 | 63.1 | 240 | 130 | 110 | 63.5 | Neurologist diagnosed PD (2 of 4 cardinal signs) | Structured questionnaire | None |
| 89 | 1998 | Chan | Hong Kong | - | 2 Hospitals in Hong Kong | 215 | 114 | 101 | - | 313 | 171 | 142 | - | Neurologist diagnosed PD (Maranganore criteria) | Structured interview | Smoking, FHx, tea, rural living, dietary factors, farming |
| 117 | 1998 | de Palma | Italy | - | Institute of Neurology, University of Palma | 100 | 59 | 41 | 66.6 | 200 | 118 | 82 | 64.2 | UK Brain Bank Criteria | Structured questionnaire | None |
| 71 | 1998 | McCann | Australia | - | Clinics and Residential Care Centres, Queensland and New South Wales | 224 | 131 | 93 | 70.3 | 310 | 193 | 117 | 68.9 | PD diagnosed according to Calne criteria | Questionnaire | None |
| 175 | 1999 | Fall | Sweden | 1989 | Ostergotland, Southwest Sweden | 113 | - | - | - | 263 | - | - | - | Neurologist confirmed PD | Structured questionnaire | None |
| 120 | 1999 | Kuopio | Finland | 1994-1996 | Nine rural municipalities in Finland | 123 | 63 | 60 | 68.7 | 246 | 126 | 120 | 69.3 | Neurologist diagnosed | Structured interview | None |
| 81 | 1999 | Taylor | US | - | Movement Disorder Centre at Boston Medical Centre | 140 | 88 | 52 |  | 147 | 90 | 57 |  | Neurologist diagnosed PD (Ward and Gibb criteria) | Structured interview | Birth cohort, sex, head injury, FHx, depression, education, urban living, suburban living, rural living, herbicides, well water, smoking, vitamins |
| 90 | 1999 | Werneck | Brazil | 1996-1997 | Neurology Department of IASERJ Central Hospital | 92 | 41 | 51 | 70.6 | 110 | 47 | 63 | 68.4 | Neurologist diagnosed PD (Calne criteria) | Structured questionnaire | None |
| 62 | 2001 | Herishanu | Israel | 1989-1995 | PD clinic of Soroka University Medical Centre | 93 | - | - | - | 93 | - | - | - | Progressive disorder, 2 or more cardinal signs of PD | Interview with structured questionnaire | Smoking, construction work, mechanical factory work, country of birth, peptic ulcer disease |
| 82 | 2002 | Zorzon | Italy | 1998 | Center for Parkinson's disease and Movement Disorders, Trieste | 136 | 62 | 74 | 70 | 272 | 124 | 148 | 69 | Neurologist diagnosed PD (2 of 4 cardinal signs) | Structured interview | Smoking |
| 123 | 2003 | Baldereschi | Italy | 1992-1993 | Italian Longitudinal Study on Aging | 113 | 58 | 57 | 78.1 | 4383 | 2247 | 2136 | 74.5 | Medical record (2 of 4 cardinal signs) | Structured questionnaire | Age, gender, education, smoking |
| 124 | 2003 | Baldi | France | 1997-1999 | Gironde and Dordogne | 84 | 44 | 40 | 75.6 | 252 | 132 | 120 | 75.5 | UK Brain Bank Criteria | Structured interview | Education, smoking |
| 60 | 2003 | Duzcan | Turkey | 2000 | Kizilcaboluk-Denizli | 36 | 17 | 19 | - | 108 | 51 | 57 | - | Neurologist diagnosed Parkinsonism (2 of 4 cardinal signs) + levodopa response | Questionnaire | None |
| 176 | 2004 | Gorell | US | 1988-1992 | Henry Ford Health System, Detroit | 144 | - | - | - | 464 | - | - | - | ICD code for PD, medical record review | Structured interview | Age, sex, race, smoking |
| 147 | 2004 | Nuti | Italy | - | Lucca and Pistoia, Tuscany | 190 | 106 | 84 | 63.9 | 190 | 106 | 84 | 62.8 | UK Brain Bank Criteria | Structured interview | None |
| 92 | 2005 | Galanaud | France | 1998-1999 | Mutualite Sociale Agricole, French health insurance system | 247 | 138 | 109 | 69 | 676 | 377 | 299 | 69 | Neurologist diagnosed PD (2 or more cardinal signs) | Structured interview | Age, education |
| 177 | 2006 | Frigerio | US | 1976-1995 | Rochester Epidemiology Project, MN | 149 | 90 | 59 | - | 129 | - | - | - | Medical record review (2 of 4 cardinal signs) | Medical record review | None |
| 59 | 2007 | Dick | UK | 2000-2004 | GEOPARKINSON study | 649 | - | - | - | 1587 | - | - | - | Physician/medical record (UK Brain Bank criteria) | Structured interview | Age, sex, country, tobacco, unconscious, 1st degree relative |
| 178 | 2007 | Fong | Taiwan | 2001-2003 | Neurology Department of Dalin Tzu Chi General Hospital, Chia Yi | 153 | 72 | 81 | 71.7 | 155 | 66 | 89 | 70 | Neurologist diagnosed (3 of 4 cardinal signs) | Structured interview | Sex |
| 131 | 2007 | Kamel | US | 1993-2003 | Agricultural Health Study | 78 | - | - | - | 55931 | - | - | - | Physician diagnosed PD | Structured questionnaire | Age, state |
| 133 | 2008 | Petersen |  | 2005 | Faroe Islands | 79 | 43 | 36 | 74.4 | 154 | 85 | 69 | 75.2 | Neurologist diagnosed | Structured questionnaire | Smoking |
| 137 | 2009 | Tanner | US | 2004-2007 | 8 North American Movement Disorder clinics | 519 | 309 | 210 | 65 | 511 | 302 | 209 | 65 | Neurologist diagnosed PD (2 of 4 cardinal signs) | Structured interview | Age, sex, race, smoking, caffeine, alcohol, head injury |
| 179 | 2010 | Firestone | US | 1992-2006 | Group Health Cooperative, Seattle area | 404 | 252 | 152 | 69 | 526 | 326 | 200 | 71 | Neurologist diagnosed or medical record review | Structured questionnaire | Age, ethnicity, smoking |
| 93 | 2010 | Sanyal | India | - | Movement Disorders clinic in Kolkata | 175 | 140 | 35 | 55.2 | 350 | 280 | 70 | 55 | 3 of 4 cardinal signs of PD | Structured interview | FHx, rural living, depression, toxins, smoking |
| 249 | 2010 | Skeie | Norway | 2004-2006 | 4 counties in Norway | 212 | 126 | 86 | - | 175 | 104 | 71 | 67.5 | Gelb diagnostic criteria | Structured interview | None |
| 180 | 2010 | Hristina | Serbia | 2001-2005 | Institute of Neurology, School of Medicine, Belgrade University | 110 | 63 | 47 | - | 220 | 126 | 94 | - | Neurologist diagnosed PD (2 of 4 cardinal signs) | Structured questionnaire | Gardening, dyes, naptha, well water, spring water, service sector worker |
|  |  |  |  |  |  |  |  |  |  |  |  |  |  |  |  |  |
|  | Cohort studies - Pesticide exposure | | | | | |  |  |  |  |  |  |  |  |  |  |
|  |  |  |  |  |  |  |  |  |  |  |  |  |  |  |  |  |
| **Ref** | **Year** | **Author** | **Country** | **Mean follow up (yrs)** | **Resource** | **Cohort** | **Male** | **Female** | **Cases** | **Male** | **Female** | **Mean Age** |  | **Definition** | **Exposure Assessment** | **Matching for** |
| 181 | 2003 | Baldi | France | 10 | PAQUID study | 1507 | - | - | 24 | 10 | 14 |  |  | Neurologist diagnosed | Structured questionnaire | Education, smoking |
| 182 | 2006 | Ascherio | US | - | Cancer Prevention Study II Nutrition Cohort | 143325 | - | - | 413 | - | - | - |  | Neurologist diagnosed | Structured questionnaire | Age, sex, smoking |
|  |  |  |  |  |  |  |  |  |  |  |  |  |  |  |  |  |
|  |  |  |  |  |  |  |  |  |  |  |  |  |  |  |  |  |
|  | Case control studies - Farming or agricultural occupation | | | | | |  |  |  |  |  |  |  |  |  |  |
|  |  |  |  |  |  |  |  |  |  |  |  |  |  |  |  |  |
| **Ref** | **Year** | **First Author** | **Country** | **Study period** | **Resource** | **Cases** | **Male** | **Female** | **Mean Age** | **Controls** | **Male** | **Female** | **Mean Age** | **Definition** | **Exposure assessment** | **Matching for** |
| 104 | 1989 | Ho | Hong Kong | - | 10 Old Age Homes in Shatin and Tai Po | 35 | 11 | 24 | - | 105 | - | - | - | Trained medical students using diagnostic criteria | Structured interview | None |
| 172 | 1990 | Koller | US | - | Movement Disorder clinic, University of Kansas Medical Centre | 150 | 89 | 61 | 66 | 150 | 89 | 61 | 66.3 | Neurologist diagnosed PD (2 of 4 cardinal signs) | Structured interview | None |
| 183 | 1991 | Semchuk | Canada | 1984-1987 | Calgary Residents | 130 | 75 | 55 | 68.5 | 260 | 150 | 110 | 68.3 | Neurologist diagnosed PD (2 of 4 cardinal signs) | Structured interview | Rural living, well water |
| 109 | 1991 | Wechsler | US | - | Neurology Clinic at University of Washington and PD support groups | 34 | - | - | 68.4 | 25 | - | - | 58.9 | - | Structured questionnaire | None |
| 173 | 1994 | Hertzman | Canada | - | Okanagan Valley, British Columbia | 127 | 71 | 56 | 70.5 | 124 | 60 | 64 | 69.6 | Neurologist diagnosed PD (2 of 4 cardinal signs) | Structured interview | None |
| 84 | 1994 | Morano | Spain | 1989-1990 | General Hospitals in Caceres, Spain | 74 | 33 | 41 | 68.2 | 148 | 66 | 82 | 67.5 | Diagnostic criteria for PD | Structured questionnaire | None |
| 184 | 1996 | Rocca | Italy | 1987 | Sicily | 62 | 27 | 32 | - | 124 | - | - | - | Neurologist diagnosed PD (2 of 4 cardinal signs) | Structured interview | None |
| 88 | 1996 | Seidler | Germany | - | Nine Neurology clinics across Germany | 380 | 251 | 129 | 56.2 | 359 | - | - | 56.5 | Neurologist diagnosed (UK Brain Bank Criteria) | Structured interview | Smoking, education |
| 115 | 1997 | Liou | China | 1993-1995 | Movement Disorder clinic, National Taiwan University Hospital | 120 | 65 | 55 | 63.1 | 240 | 130 | 110 | 63.5 | Neurologist diagnosed PD (2 of 4 cardinal signs) | Structured questionnaire | None |
| 89 | 1998 | Chan | Hong Kong | - | 2 Hospitals in Hong Kong | 215 | 114 | 101 | - | 313 | 171 | 142 | - | Neurologist diagnosed PD (Maranganore criteria) | Structured interview | Smoking, pesticides, tea, FHx, rural living, dietary factors, well water |
| 175 | 1999 | Fall | Sweden | 1989 | Ostergotland, Southwest Sweden | 113 | - | - | - | 263 | - | - | - | Neurologist confirmed PD | Structured questionnaire | None |
| 120 | 1999 | Kuopio | Finland | 1994-1996 | Nine rural municipalities in Finland | 123 | 63 | 60 | 68.7 | 246 | 126 | 120 | 69.3 | Neurologist diagnosed | Structured interview | None |
| 91 | 2001 | Behari | India | 1994-1998 | Movement Disorder clinic of AIIMS, New Dehli | 377 | 301 | 76 | 56.8 | 377 | 271 | 106 | 56.6 | Neurologist diagnosed PD (2 of 3 cardinal signs) | Structured questionnaire | None |
| 185 | 2001 | Kirkey | US | 1988-1992 | Henry Ford Health System, Detroit | 144 | 90 | 54 | - | 464 | 293 | 171 | - | Neurologist diagnosed PD | Structured interview | None |
| 82 | 2002 | Zorzon | Italy | 1998 | Center for Parkinson's disease and Movement Disorders, Trieste | 136 | 62 | 74 | 70 | 272 | 124 | 148 | 69 | Neurologist diagnosed PD (2 of 4 cardinal signs) | Structured interview | Smoking, FHx of PD or ET, maternal age, anaesthesia, well water |
| 60 | 2003 | Duzcan | Turkey | 2000 | Kizilcaboluk-Denizli | 36 | 17 | 19 | - | 108 | 51 | 57 | - | Neurologist diagnosed Parkinsonism (2 of 4 cardinal signs) + levodopa response | Questionnaire | None |
| 186 | 2005 | Frigerio | US | 1976-1995 | Rochester Epidemiology Project, Olmsted County, Minnesota | 196 | 121 | 75 | 71 | 196 | 121 | 75 | - | Medical record review (2 of 4 cardinal signs) | Medical record review | Sex, age |
| 92 | 2005 | Galanaud | France | 1998-1999 | Mutualite Sociale Agricole, French health insurance system | 247 | 138 | 109 | 69 | 676 | 377 | 299 | 69 | Neurologist diagnosed PD (2 or more cardinal signs) | Structured interview | Age, sex, education, alcohol, FHx, pesticides, smoking |
| 187 | 2005 | Park | South Korea | 2001 | Five nuerological clinics in Seoul and Busan | 367 | 177 | 190 | - | 309 | 198 | 111 | - | Neurologist diagnosed (Gibb and Lees) | Structured questionnaire | Sex, age, smoking, education |
| 188 | 2007 | Dick | UK | 2000-2004 | GEOPARKINSON study | 649 | - | - | - | 1587 | - | - | - | Physician/medical record (UK Brain Bank criteria) | Structured interview | Age, gender, tobacco, 1st degree relative |
| 137 | 2009 | Tanner | US | 2004-2007 | 8 North American Movement Disorder clinics | 519 | 309 | 210 | 65 | 511 | 302 | 209 | 65 | Neurologist diagnosed PD (2 of 4 cardinal signs) | Structured interview | Sex, age, ethnicity, smoking, caffeine, alcohol, head injury |
| 179 | 2010 | Firestone | US | 1992-2006 | Group Health Cooperative, Seattle area | 404 | 252 | 152 | 69 | 526 | 326 | 200 | 71 | Neurologist diagnosed or medical record review | Structured interview | Age, ethnicity, smoking |
| 249 | 2010 | Skeie | Norway | 2004-2006 | 4 counties in Norway | 212 | 126 | 86 | - | 175 | 104 | 71 | 67.5 | Gelb diagnostic criteria | Structured interview | None |
| 93 | 2010 | Sanyal | India | - | Movement Disorders clinic in Kolkata | 175 | 140 | 35 | 55.2 | 350 | 280 | 70 | 55 | 3 of 4 cardinal signs of PD | Structured interview | None |
|  |  |  |  |  |  |  |  |  |  |  |  |  |  |  |  |  |
|  | Cohort study - Farming or agricultural occupation | | | | | |  |  |  |  |  |  |  |  |  |  |
|  |  |  |  |  |  |  |  |  |  |  |  |  |  |  |  |  |
| **Ref** | **Year** | **Author** | **Country** | **Mean follow up (yrs)** | **Resource** | **Cohort** | **Male** | **Female** | **Cases** | **Male** | **Female** | **Mean Age** |  | **Definition** | **Exposure Assessment** | **Matching for** |
| 181 | 2003 | Baldi | France | 10 | PAQUID study | 1507 | - | - | 24 | 10 | 14 | - |  | Neurologist diagnosed | Structured questionnaire | Education, smoking |
|  |  |  |  |  |  |  |  |  |  |  |  |  |  |  |  |  |
|  |  |  |  |  |  |  |  |  |  |  |  |  |  |  |  |  |
|  | Case control studies - Well water exposure | | | | | |  |  |  |  |  |  |  |  |  |  |
|  |  |  |  |  |  |  |  |  |  |  |  |  |  |  |  |  |
| **Ref** | **Year** | **First Author** | **Country** | **Study period** | **Resource** | **Cases** | **Male** | **Female** | **Mean Age** | **Controls** | **Male** | **Female** | **Mean Age** | **Definition** | **Exposure assessment** | **Matching for** |
| 189 | 1989 | Tanner | China | - | 3 Hospitals in Beijing and Guangzhou | 100 | 77 | 23 | 57.2 | 200 | 154 | 46 | - | Neurologist diagnosed PD (2 of 4 cardinal signs) | Structured interview | None |
| 171 | 1990 | Golbe | US | 1986-1988 | Movement disorder centre at UMNDNJ-Robert Wood Johnson Medical School | 106 | 71 | 35 | - | 106 | 35 | 71 | - | Neurologist diagnosed PD | Structured questionnaire | None |
| 172 | 1990 | Koller | US | - | Movement Disorder clinic, University of Kansas Medical Centre | 150 | 89 | 61 | 66 | 150 | 89 | 61 | 66.3 | Neurologist diagnosed PD (2 of 4 cardinal signs) | Structured interview | None |
| 183 | 1991 | Semchuk | Canada | 1984-1987 | Calgary Residents | 130 | 75 | 55 | 68.5 | 260 | 150 | 110 | 68.3 | Neurologist diagnosed PD (2 of 4 cardinal signs) | Structured interview | Farming, rural living |
| 109 | 1991 | Wechsler | US | - | Neurology Clinic at University of Washington and PD support groups | 34 | - | - | 68.4 | 25 | - | - | 58.9 | - | Structured questionnaire | None |
| 111 | 1992 | Jimenez-Jimenez | Spain | - | Hospital General Gregario Maranon, Madrid | 128 | 67 | 61 | 66.8 | 256 | 134 | 122 | 64.8 | Not specified | Structured questionnaire | None |
| 83 | 1993 | Wang | China | - | Department of Neurology, Tianjin General Hospital | 93 | 63 | 30 | 61 | 186 | 126 | 60 | 60 | Neurologist diagnosed PD (2 or more cardinal signs) | Structured interview | None |
| 173 | 1994 | Hertzman | Canada | - | Okanagan Valley, British Columbia | 127 | 71 | 56 | 70.5 | 124 | 60 | 64 | 69.6 | Neurologist diagnosed PD (2 of 4 cardinal signs) | Structured interview | None |
| 84 | 1994 | Morano | Spain | 1989-1990 | General Hospitals in Caceres, Spain | 74 | 33 | 41 | 68.2 | 148 | 66 | 82 | 67.5 | Diagnostic criteria for PD | Structured questionnaire | None |
| 87 | 1996 | de Michele | Italy | - | Department of Neurology Federico II University in Naples | 116 | 77 | 39 | 62.5 | 232 | 116 | 116 | 62.4 | 2 of 3 cardinal signs of PD | Structured questionnaire | None |
| 88 | 1996 | Seidler | Germany | - | Nine Neurology clinics across Germany | 380 | 251 | 129 | 56.2 | 359 | - | - | 56.5 | Neurologist diagnosed (UK Brain Bank Criteria) | Structured interview | Smoking, education |
| 115 | 1997 | Liou | China | 1993-1995 | Movement Disorder clinic, National Taiwan University Hospital | 120 | 65 | 55 | 63.1 | 240 | 130 | 110 | 63.5 | Neurologist diagnosed PD (2 of 4 cardinal signs) | Structured questionnaire | None |
| 89 | 1998 | Chan | Hong Kong | - | 2 Hospitals in Hong Kong | 215 | 114 | 101 | - | 313 | 171 | 142 | - | Neurologist diagnosed PD (Maranganore criteria) | Structured interview | Smoking, pesticides, tea, FHx, rural living, dietary factors, farming |
| 190 | 1998 | Gorell | US | 1991-1995 | Henry Ford Health System Cohort, Detroit | 144 | 89 | 55 | 70 | 464 | - | - |  | Neurologist diagnosed PD | Interview with structured questionnaire | Race, sex, age, smoking |
| 71 | 1998 | McCann | Australia | - | Clinics and Residential Care Centres, Queensland and New South Wales | 224 | 131 | 93 | 70.3 | 310 | 193 | 117 | 68.9 | Calne criteria | Questionnaire | Age, sex, rural residency, HTN, FHx, stroke |
| 118 | 1998 | Smargiassi | Italy | - | Emilia-Romagna Region, Italy | 86 | 50 | 36 | 66.4 | 86 | 48 | 38 | 63.1 | Neurologist diagnosed (UK Brain Bank Criteria) | Structured questionnaire | None |
| 117 | 1998 | de Palma | Italy | - | Institute of Neurology, University of Palma | 100 | 59 | 41 | 66.6 | 200 | 118 | 82 | 64.2 | UK Brain Bank Criteria | Structured questionnaire | None |
| 81 | 1999 | Taylor | US | - | Movement Disorder Centre at Boston Medical Centre | 140 | 88 | 52 | 66.2 | 147 | 90 | 57 | 66.9 | Neurologist diagnosed PD (Ward and Gibb criteria) | Structured interview | Birth cohort, sex, head injury, FHx PD, FHx ET, depression, education, rural living, urban living, pesticides, smoking, vitamins |
| 90 | 1999 | Werneck | Brazil | 1996-1997 | Neurology Department of IASERJ Central Hospital | 92 | 41 | 51 | 70.6 | 110 | 47 | 63 | 68.4 | Neurologist diagnosed PD (Calne criteria) | Structured questionnaire | None |
| 91 | 2001 | Behari | India | 1994-1998 | Movement Disorder clinic of AIIMS, New Dehli | 377 | 301 | 76 | 56.8 | 377 | 271 | 106 | 56.6 | Neurologist diagnosed PD (2 of 3 cardinal signs) | Structured questionnaire | Age |
| 82 | 2002 | Zorzon | Italy | 1998 | Center for Parkinson's disease and Movement Disorders, Trieste | 136 | 62 | 74 | 70 | 272 | 124 | 148 | 69 | Neurologist diagnosed PD (2 of 4 cardinal signs) | Structured interview | Smoking, FHx of PD or ET, maternal age, anaesthesia, farming |
| 147 | 2004 | Nuti | Italy | - | Lucca and Pistoia, Tuscany | 190 | 106 | 84 | 63.9 | 190 | 106 | 84 | 62.8 | UK Brain Bank Criteria | Structured interview | None |
| 191 | 2004 | Park | Korea | 2000-2004 | University Hospital in Busan | 105 | 42 | 63 | 63.6 | 101 | 44 | 57 | 58.4 | UK Brain Bank Criteria | Structured questionnaire | None |
| 192 | 2005 | Firestone | US | 1992-2002 | Group Health Cooperative, Seattle area | 250 | 156 | 94 | 70.1 | 388 | 241 | 147 | 70.8 | Neurologist diagnosed or medical record review | Structured questionnaire | Age, sex, smoking |
| 59 | 2007 | Dick | UK | 2000-2004 | GEOPARKINSON study | 767 | 426 | 341 | 69.8 | 1989 | 1057 | 932 | 69.8 | Physician/medical record (UK Brain Bank criteria) | Interview | Tobacco, alcohol, LOC, anaesthetic, gas/smoke exposure, sleeping pills, anxiety Rx, depression Rx, FHx, solvents, pesticides, iron, manganese, copper |
| 136 | 2009 | Gatto | US | 2001-2007 | Parkinson's Environment and Genes Study | 368 | 207 | 161 | 69.6 | 341 | 176 | 165 | 67.6 | Neurologist diagnosed PD (2 of 4 cardinal signs and absence of atypical features) | Telephone interview | None |
| 180 | 2010 | Hristina | Serbia | 2001-2005 | Institute of Neurology, School of Medicine, Belgrade University | 110 | 63 | 47 | - | 220 | 126 | 94 | - | Neurologist diagnosed PD (2 of 4 cardinal signs) | Structured questionnaire | Gardening, insecticide exposure, dyes, naptha, spring water, service sector worker |
| 93 | 2010 | Sanyal | India | - | Movement Disorders clinic in Kolkata | 175 | 140 | 35 | 55.2 | 350 | 280 | 70 | 55 | 3 of 4 cardinal signs of PD | Structured interview | None |
|  |  |  |  |  |  |  |  |  |  |  |  |  |  |  |  |  |
|  | Case control studies - Rural living | | | | | |  |  |  |  |  |  |  |  |  |  |
|  |  |  |  |  |  |  |  |  |  |  |  |  |  |  |  |  |
| **Ref** | **Year** | **Author** | **Country** | **Study period** | **Resource** | **Cases** | **Male** | **Female** | **Mean Age** | **Control** | **Male** | **Female** | **Mean Age** | **Definition** | **Exposure assessment** | **Matching for** |
| 104 | 1989 | Ho | Hong Kong | - | 10 Old Age Homes in Shatin and Tai Po | 35 | 11 | 24 | - | 105 | - | - | - | Trained medical students using diagnostic criteria | Structured interview | None |
| 171 | 1990 | Golbe | US | 1986-1988 | Movement disorder centre at UMNDNJ-Robert Wood Johnson Medical School | 106 | 71 | 35 | - | 106 | 35 | 71 | - | Neurologist diagnosed PD | Structured questionnaire | None |
| 172 | 1990 | Koller | US | - | Movement Disorder clinic, University of Kansas Medical Centre | 150 | 89 | 61 | 66 | 150 | 89 | 61 | 66.3 | Neurologist diagnosed PD (2 of 4 cardinal signs) | Structured interview | None |
| 183 | 1991 | Semchuk | Canada | 1984-1987 | Calgary Residents | 130 | 75 | 55 | 68.5 | 260 | 150 | 110 | 68.3 | Neurologist diagnosed PD (2 of 4 cardinal signs) | Structured interview | Farming, well water |
| 111 | 1992 | Jimenez-Jimenez | Spain | - | Hospital General Gregario Maranon, Madrid | 128 | 67 | 61 | 66.8 | 256 | 134 | 122 | 64.8 | Not specified | Structured questionnaire | None |
| 83 | 1993 | Wang | China | - | Department of Neurology, Tianjin General Hospital | 93 | 63 | 30 | 61 | 186 | 126 | 60 | 60 | Neurologist diagnosed PD (2 or more cardinal signs) | Structured interview | None |
| 84 | 1994 | Morano | Spain | 1989-1990 | General Hospitals in Caceres, Spain | 74 | 33 | 41 | 68.2 | 148 | 66 | 82 | 67.5 | Diagnostic criteria for PD | Structured questionnaire | None |
| 88 | 1996 | Seidler | Germany | - | Nine Neurology clinics across Germany | 380 | 251 | 129 | 56.2 | 359 | - | - | 56.5 | Neurologist diagnosed (UK Brain Bank Criteria) | Structured interview | Smoking, education |
| 115 | 1997 | Liou | China | 1993-1995 | Movement Disorder clinic, National Taiwan University Hospital | 120 | 65 | 55 | 63.1 | 240 | 130 | 110 | 63.5 | Neurologist diagnosed PD (2 of 4 cardinal signs) | Structured questionnaire | None |
| 71 | 1998 | McCann | Australia | - | Clinics and Residential Care Centres, Queensland and New South Wales | 224 | 131 | 93 | 70.3 | 310 | 193 | 117 | 68.9 | Calne criteria | Questionnaire | Age, sex, well water, HTN, FHx, stroke |
| 117 | 1998 | de Palma | Italy | - | Institute of Neurology, University of Palma | 100 | 59 | 41 | 66.6 | 200 | 118 | 82 | 64.2 | UK Brain Bank Criteria | Structured questionnaire | None |
| 118 | 1998 | Smargiassi | Italy | - | Emilia-Romagna Region, Italy | 86 | 50 | 36 | 66.4 | 86 | 48 | 38 | 63.1 | Neurologist diagnosed (UK Brain Bank Criteria) | Structured questionnaire | None |
| 90 | 1999 | Werneck | Brazil | 1996-1997 | Neurology Department of IASERJ Central Hospital | 92 | 41 | 51 | 70.6 | 110 | 47 | 63 | 68.4 | Neurologist diagnosed PD (Calne criteria) | Structured questionnaire | None |
| 74 | 2000 | Preux | France | 1995-1996 | Limoges University Hospital, France | 140 | - | - | - | 280 | - | - | - | Physician examined (UK Brain Bank Criteria) | Structured interview | Smoking, coffee, tea, FHx, toxic products |
| 91 | 2001 | Behari | India | 1994-1998 | Movement Disorder clinic of AIIMS, New Dehli | 377 | 301 | 76 | 56.8 | 377 | 271 | 106 | 56.6 | Neurologist diagnosed PD (2 of 3 cardinal signs) | Structured questionnaire | Age |
| 82 | 2002 | Zorzon | Italy | 1998 | Center for Parkinson's disease and Movement Disorders, Trieste | 136 | 62 | 74 | 70 | 272 | 124 | 148 | 69 | Neurologist diagnosed PD (2 of 4 cardinal signs) | Structured interview | Smoking |
| 147 | 2004 | Nuti | Italy | - | Lucca and Pistoia, Tuscany | 190 | 106 | 84 | 63.9 | 190 | 106 | 84 | 62.8 | UK Brain Bank Criteria | Structured interview | None |
| 93 | 2010 | Sanyal | India | - | Movement Disorders clinic in Kolkata | 175 | 140 | 35 | 55.2 | 350 | 280 | 70 | 55 | 3 of 4 cardinal signs of PD | Structured interview | Pesticides, FHx, depression, toxins, smoking |
|  |  |  |  |  |  |  |  |  |  |  |  |  |  |  |  |  |
|  | Cohort study - Rural living | | | | | |  |  |  |  |  |  |  |  |  |  |
|  |  |  |  |  |  |  |  |  |  |  |  |  |  |  |  |  |
| **Ref** | **Year** | **Author** | **Country** | **Mean follow up (yrs)** | **Resource** | **Cohort** | **Male** | **Female** | **Cases** | **Male** | **Female** | **Mean Age** |  | **Definition** | **Exposure Assessment** | **Matching for** |
| 181 | 2003 | Baldi | France | 10 | PAQUID study | 1507 | - | - | 24 | 10 | 14 |  |  | Neurologist diagnosed | Structured questionnaire | Education, smoking |
