# Supplementary material for: Meta-Analysis of Early Nonmotor Features and Risk Factors for Parkinson Disease
Source: Ann Neurol. 2012 Oct 15;72(6):893–901. doi: 10.1002/ana.23687 (PMC3556649; doi:10.1002/ana.23687)
Supplement: Supplementary file 3 [file ana0072-0893-SD3.doc]

|  | **Table 2 - Odds ratios and relative risks of studies included in the meta-analysis** | | | | | | | | |  |  |  |  |  |  |  |  |
| --- | --- | --- | --- | --- | --- | --- | --- | --- | --- | --- | --- | --- | --- | --- | --- | --- | --- |
|  |  |  |  |  |  |  |  |  |  |  |  |  |  |  |  |  |  |
|  | First degree relative with PD | | | | | |  |  |  |  |  |  |  |  |  |  |  |
|  |  |  |  | Relative risk |  | Overall |  |  |  |  |  |  |  |  |  |  |  |
| Ref |  | Year | First author | (95% CI) |  | weight |  |  |  |  |  |  |  |  |  |  |  |
|  | **Case-control studies** | | |  |  |  |  |  |  |  |  |  |  |  |  |  |  |
| 70 |  | 1973 | Martin | 2·45 (1·21 to 5·27) |  | 3.90 |  |  |  |  |  |  |  |  |  |  |  |
| 57 |  | 1986 | Alonso | 10·42 (1·42 to 456·64) | | 0.44 |  |  |  |  |  |  |  |  |  |  |  |
| 78 |  | 1993 | Semchuck | 2·23 (0·94 to 5·29) |  | 3.23 |  |  |  |  |  |  |  |  |  |  |  |
| 68 |  | 1996 | Marder | 2·30 (1·30 to 4·00) |  | 5.03 |  |  |  |  |  |  |  |  |  |  |  |
| 72 |  | 1997 | Mickel | 3·69 (1·36 to 10·00) |  | 2.68 |  |  |  |  |  |  |  |  |  |  |  |
| 71 |  | 1998 | McCann | 2·36 (1·15 to 4·96) |  | 3.92 |  |  |  |  |  |  |  |  |  |  |  |
| 77 |  | 1999 | Rybicki | 4·00 (2·00 to 8·10) |  | 4.11 |  |  |  |  |  |  |  |  |  |  |  |
| 81 |  | 1999 | Taylor | 3·40 (1·82 to 6·30) |  | 4.61 |  |  |  |  |  |  |  |  |  |  |  |
| 58 |  | 2000 | Autere | 2·90 (1·30 to 6·40) |  | 3.56 |  |  |  |  |  |  |  |  |  |  |  |
| 61 |  | 2000 | Elbaz | 3·50 (1·60 to 7·60) |  | 3.66 |  |  |  |  |  |  |  |  |  |  |  |
| 67 |  | 2000 | la Fuente-Fernandez | 4·25 (2·20 to 8·73) |  | 4.17 |  |  |  |  |  |  |  |  |  |  |  |
| 74 |  | 2000 | Preux | 9·30 (2·60 to 32·60) |  | 1.89 |  |  |  |  |  |  |  |  |  |  |  |
| 62 |  | 2001 | Herishanu | 2·44 (0·53 to 15·03) |  | 1.20 |  |  |  |  |  |  |  |  |  |  |  |
| 65 |  | 2001 | Kuopio | 2·70 (1·30 to 5·90) |  | 3.78 |  |  |  |  |  |  |  |  |  |  |  |
| 73 |  | 2002 | Payami | 3·92 (1·59 to 9·68) |  | 3.06 |  |  |  |  |  |  |  |  |  |  |  |
| 82 |  | 2002 | Zorzon | 41·70 (12·20 to 142·50) | | 1.98 |  |  |  |  |  |  |  |  |  |  |  |
| 60 |  | 2003 | Duzcan | 7·48 (2·52 to 22·17) |  | 2.37 |  |  |  |  |  |  |  |  |  |  |  |
| 66 |  | 2003 | Kurz | 4·51 (1·35 to 23·58) |  | 1.56 |  |  |  |  |  |  |  |  |  |  |  |
| 69 |  | 2003 | Marder | 2·70 (1·70 to 4·40) |  | 5.69 |  |  |  |  |  |  |  |  |  |  |  |
| 64 |  | 2004 | Korchounov | 3·70 (2·52 to 5·52) |  | 6.36 |  |  |  |  |  |  |  |  |  |  |  |
| 75 |  | 2004 | Rocca | 1·54 (0·86 to 2·76) |  | 4.88 |  |  |  |  |  |  |  |  |  |  |  |
| 80 |  | 2004 | Spanaki | 3·37 (1·90 to 6·04) |  | 4.91 |  |  |  |  |  |  |  |  |  |  |  |
| 59 |  | 2007 | Dick | 4·63 (3·21 to 6·69) |  | 6.56 |  |  |  |  |  |  |  |  |  |  |  |
| 76 |  | 2007 | Rosen | 2·20 (1·20 to 4·00) |  | 4.74 |  |  |  |  |  |  |  |  |  |  |  |
| 79 |  | 2010 | Shino | 3·40 (1·90 to 5·90) |  | 5.00 |  |  |  |  |  |  |  |  |  |  |  |
| 63 |  | 2010 | Jacob | 1·70 (1·12 to 2·24) |  | 6.73 |  |  |  |  |  |  |  |  |  |  |  |
|  |  |  |  |  |  |  |  |  |  |  |  |  |  |  |  |  |  |
|  | Overall | |  | 3·23 (2·65 to 3·93) | | I2=52·2%, p=0·001 |  |  |  |  |  |  |  |  |  |  |  |
|  |  |  |  |  |  |  |  |  |  |  |  |  |  |  |  |  |  |
|  | Any family history of PD | | | | | |  |  |  |  |  |  |  |  |  |  |  |
|  |  |  |  | Relative risk |  | Overall |  |  |  |  |  |  |  |  |  |  |  |
| Ref |  | Year | First author | (95% CI) |  | weight |  |  |  |  |  |  |  |  |  |  |  |
|  | **Case-control studies** | | |  |  |  |  |  |  |  |  |  |  |  |  |  |  |
| 78 |  | 1993 | Semchuk | 5·80 (2·60 to 12·80) |  | 5.61 |  |  |  |  |  |  |  |  |  |  |  |
| 83 |  | 1993 | Wang | 4·30 (1·70 to 11·30) |  | 4.68 |  |  |  |  |  |  |  |  |  |  |  |
| 84 |  | 1994 | Morano | 3·90 (1·30 to 12·20) |  | 3.81 |  |  |  |  |  |  |  |  |  |  |  |
| 85 |  | 1995 | Bonifati | 4·95 (2·05 to 11·94) |  | 5.07 |  |  |  |  |  |  |  |  |  |  |  |
| 86 |  | 1995 | Vieregge | 7·10 (0·80 to 60·60) |  | 1.37 |  |  |  |  |  |  |  |  |  |  |  |
| 87 |  | 1996 | de Michele | 14·60 (7·20 to 29·60) | | 6.26 |  |  |  |  |  |  |  |  |  |  |  |
| 88 |  | 1996 | Seidler | 5·00 (2·40 to 10·70) |  | 5.96 |  |  |  |  |  |  |  |  |  |  |  |
| 89 |  | 1998 | Chan | 4·00 (0·80 to 20·40) |  | 2.23 |  |  |  |  |  |  |  |  |  |  |  |
| 71 |  | 1998 | McCann | 3·70 (2·00 to 6·60) |  | 7.13 |  |  |  |  |  |  |  |  |  |  |  |
| 77 |  | 1999 | Rybicki | 4·20 (2·30 to 7·60) |  | 7.13 |  |  |  |  |  |  |  |  |  |  |  |
| 90 |  | 1999 | Werneck | 14·10 (3·00 to 91·40) | | 2.05 |  |  |  |  |  |  |  |  |  |  |  |
| 91 |  | 2001 | Behari | 7·00 (2·09 to 36·63) |  | 2.70 |  |  |  |  |  |  |  |  |  |  |  |
| 65 |  | 2001 | Kuopio | 2·80 (1·60 to 5·00) |  | 7.36 |  |  |  |  |  |  |  |  |  |  |  |
| 60 |  | 2003 | Duzcan | 4·40 (1·90 to 10·45) |  | 5.25 |  |  |  |  |  |  |  |  |  |  |  |
| 66 |  | 2003 | Kurz | 5·24 (2·01 to 17·31) |  | 4.01 |  |  |  |  |  |  |  |  |  |  |  |
| 64 |  | 2004 | Kourchounov | 3·50 (2·50 to 4·90) |  | 9.33 |  |  |  |  |  |  |  |  |  |  |  |
| 92 |  | 2005 | Galanaud | 1·90 (1·20 to 2·90) |  | 8.46 |  |  |  |  |  |  |  |  |  |  |  |
| 76 |  | 2007 | Rosen | 2·40 (1·50 to 3·90) |  | 8.14 |  |  |  |  |  |  |  |  |  |  |  |
| 93 |  | 2010 | Sanyal | 21·40 (6·36 to 70·10) | | 3.48 |  |  |  |  |  |  |  |  |  |  |  |
|  | Overall | |  | 4·45 (3·39 to 5·83) | | I2=57·3%, p=0·001 |  |  |  |  |  |  |  |  |  |  |  |
|  |  |  |  |  |  |  |  |  |  |  |  |  |  |  |  |  |  |
|  | Any family history of tremor | | | | | |  |  |  |  |  |  |  |  |  |  |  |
|  |  |  |  | Relative risk |  | Overall |  |  |  |  |  |  |  |  |  |  |  |
| Ref |  | Year | First author | (95% CI) |  | weight |  |  |  |  |  |  |  |  |  |  |  |
|  | **Case-control studies** | | |  |  |  |  |  |  |  |  |  |  |  |  |  |  |
| 94 |  | 1986 | Lang | 3·30 (1·30 to 8·40) |  | 8.01 |  |  |  |  |  |  |  |  |  |  |  |
| 78 |  | 1993 | Semchuk | 2·40 (1·20 to 4·70) |  | 14.96 |  |  |  |  |  |  |  |  |  |  |  |
| 84 |  | 1994 | Morano | 3·10 (1·20 to 8·40) |  | 7.36 |  |  |  |  |  |  |  |  |  |  |  |
| 95 |  | 1995 | Jankovic | 2·20 (1·10 to 4·30) |  | 15.00 |  |  |  |  |  |  |  |  |  |  |  |
| 86 |  | 1995 | Vieregge | 3·60 (0·90 to 14·00) |  | 3.70 |  |  |  |  |  |  |  |  |  |  |  |
| 87 |  | 1996 | de Michele | 3·10 (1·50 to 6·20) |  | 13.85 |  |  |  |  |  |  |  |  |  |  |  |
| 77 |  | 1999 | Rybicki | 2·40 (1·30 to 4·60) |  | 17.46 |  |  |  |  |  |  |  |  |  |  |  |
| 81 |  | 1999 | Taylor | 4·00 (1·20 to 13·50) |  | 4.76 |  |  |  |  |  |  |  |  |  |  |  |
| 82 |  | 2002 | Zorzon | 10·80 (2·60 to 43·70) | | 3.50 |  |  |  |  |  |  |  |  |  |  |  |
| 79 |  | 2010 | Shino | 1·90 (0·90 to 4·30) |  | 11.40 |  |  |  |  |  |  |  |  |  |  |  |
|  | Overall | |  | 2·74 (2·10 to 3·57) | | I2=0·0%, p=0·736 |  |  |  |  |  |  |  |  |  |  |  |
|  |  |  |  |  |  |  |  |  |  |  |  |  |  |  |  |  |  |
|  | Smoking | | |  |  |  |  |  |  |  |  |  |  |  |  |  |  |
|  |  |  |  | Ever smoking vs· never smoking | | | |  | Current smoking vs· never smoking | | | |  |  | Past smoking vs· never smoking | | |
|  |  |  |  |  |  | Subgroup | Overall |  |  |  |  | Subgroup | Overall |  |  | Subgroup | Overall |
| Ref |  | Year | First author | Relative risk |  | weight (%) | weight (%) |  | Relative risk |  |  | weight (%) | weight (%) |  | Relative risk | weight (%) | weight (%) |
|  | **Case-control studies** | | |  |  |  |  |  |  |  |  |  |  |  |  |  |  |
| 96 |  | 1968 | Nefzger | 0·44 (0·27 to 0·74) |  | 1·46 | 1·24 |  | 0·31 (0·18 to 0·55) | |  | 2·86 | 3·22 |  | 0·68 (0·37 to 1·21) | 2·34 | 1·68 |
| 98 |  | 1971 | Kessler | 0·48 (0·32 to 0·71) |  | 1·93 | 1·66 |  | 0·46 (0·32 to 0·66) | |  | 6·81 | 5·49 |  | 0·92 (0·67 to 1·26) | 5.00 | 3·87 |
| 97 |  | 1972 | Kessler | 0·64 (0·48 to 0·86) |  | 2·78 | 2·47 |  |  |  |  |  |  |  |  |  |  |
| 99 |  | 1980 | Baumann | 0·50 (0·36 to 0·69) |  | 2·35 | 2·05 |  |  |  |  |  |  |  |  |  |  |
| 100 |  | 1980 | Marttila | 0·74 (0·55 to 1·00) |  | 2·51 | 2·21 |  | 0·38 (0·23 to 0·62) | |  | 3·63 | 3·8 |  | 1·04 (0·73 to 1·49) | 4·45 | 3·38 |
| 101 |  | 1982 | Godwin-Austen | 0·56 (0·38 to 0·82) |  | 2.00 | 1·73 |  | 0·40 (0·25 to 0·64) | |  | 4·04 | 4·07 |  | 0·71 (0·46 to 1·09) | 3·6 | 2·67 |
| 102 |  | 1987 | Rajput | 0·70 (0·40 to 1·20) |  | 1·31 | 1·1 |  | 0·46 (0·23 to 0·92) | |  | 1·86 | 2·33 |  | 1·10 (0·60 to 2·03) | 2·24 | 1·6 |
| 103 |  | 1987 | Tanner | 0·68 (0·19 to 2·39) |  | 0·33 | 0·27 |  |  |  |  |  |  |  |  |  |  |
| 104 |  | 1989 | Ho | 0·60 (0·20 to 1·30) |  | 0·57 | 0·46 |  |  |  |  |  |  |  |  |  |  |
| 105 |  | 1989 | Hofman | 0·60 (0·30 to 1·00) |  | 1·15 | 0·96 |  | 0·70 (0·40 to 1·40) | |  | 2·27 | 2·72 |  | 0·50 (0·30 to 1·00) | 2·29 | 1·63 |
| 106 |  | 1989 | Ngim | 0·61 (0·18 to 2·03) |  | 0·36 | 0·29 |  |  |  |  |  |  |  |  |  |  |
| 107 |  | 1990 | Hertzman | 0·40 (0·19 to 0·86) |  | 0·81 | 0·67 |  |  |  |  |  |  |  |  |  |  |
| 108 |  | 1990 | Sasco | 0·97 (0·57 to 1·70) |  | 1·31 | 1·1 |  | 0·72 (0·33 to 1·63) | |  | 1·4 | 1·85 |  | 0·77 (0·34 to 1·80) | 1·36 | 0·94 |
| 109 |  | 1991 | Wechsler | 0·57 (0·16 to 1·94) |  | 0·34 | 0·28 |  |  |  |  |  |  |  |  |  |  |
| 110 |  | 1992 | Busenbark | 0·29 (0·00 to 4·27) |  | 0·05 | 0·04 |  |  |  |  |  |  |  |  |  |  |
| 111 |  | 1992 | Jimenez-Jimenez | 0·72 (0·45 to 1·13) |  | 1·64 | 1·39 |  |  |  |  |  |  |  |  |  |  |
| 78 |  | 1993 | Semchuk | 0·58 (0·33 to 1·02) |  | 1·26 | 1·05 |  |  |  |  |  |  |  |  |  |  |
| 83 |  | 1993 | Wang | 0·85 (0·54 to 1·36) |  | 1·63 | 1·39 |  |  |  |  |  |  |  |  |  |  |
| 112 |  | 1994 | Mayeux | 0·80 (0·40 to 1·50) |  | 1.00 | 0·83 |  | 0·20 (0·10 to 0·50) | |  | 1·38 | 1·83 |  | 0·90 (0·50 to 1·60) | 2·41 | 1·73 |
| 113 |  | 1995 | Martyn | 0·58 (0·39 to 0·88) |  | 1·89 | 1·62 |  | 0·49 (0·26 to 0·91) | |  | 2·27 | 2·72 |  | 0·61 (0·40 to 0·94) | 3·64 | 2·7 |
| 87 |  | 1996 | de Michele | 0·36 (0·17 to 0·73) |  | 0·86 | 0·71 |  |  |  |  |  |  |  |  |  |  |
| 114 |  | 1997 | Hellenbrand | 0·50 (0·30 to 0·70) |  | 1·8 | 1·54 |  | 0·20 (0·10 to 0·40) | |  | 1·86 | 2·33 |  | 0·80 (0·50 to 1·20) | 3·53 | 2·62 |
| 115 |  | 1997 | Liou | 0·42 (0·25 to 0·70) |  | 1·42 | 1·2 |  |  |  |  |  |  |  |  |  |  |
| 116 |  | 1997 | Tzourio | 1·10 (0·70 to 1·80) |  | 1·59 | 1·35 |  | 0·70 (0·40 to 1·30) | |  | 2·57 | 2·98 |  | 1·40 (0·90 to 2·10) | 3·67 | 2·73 |
| 89 |  | 1998 | Chan | 0·77 (0·53 to 1·10) |  | 2·11 | 1·83 |  | 0·51 (0·26 to 1·01) | |  | 1·94 | 2·41 |  | 0·91 (0·62 to 1·33) | 4·14 | 3·12 |
| 117 |  | 1998 | de Palma | 0·54 (0·34 to 0·89) |  | 1·55 | 1·32 |  |  |  |  |  |  |  |  |  |  |
| 71 |  | 1998 | McCann | 0·70 (0·40 to 1·10) |  | 1·46 | 1·23 |  |  |  |  |  |  |  |  |  |  |
| 118 |  | 1998 | Smargiassi | 0·41 (0·22 to 0·75) |  | 1·12 | 0·93 |  |  |  |  |  |  |  |  |  |  |
| 119 |  | 1999 | Gorell | 0·59 (0·40 to 0·87) |  | 1·98 | 1·71 |  |  |  |  |  |  |  |  |  |  |
| 120 |  | 1999 | Kuopio | 0·94 (0·59 to 1·48) |  | 1·64 | 1·39 |  | 0·50 (0·18 to 1·26) | |  | 0·94 | 1·32 |  | 1·08 (0·66 to 1·76) | 3·06 | 2·23 |
| 90 |  | 1999 | Werneck | 0·39 (0·16 to 0·95) |  | 0·62 | 0·51 |  |  |  |  |  |  |  |  |  |  |
| 246 |  | 2000 | Benedetti |  |  |  |  |  | 1·14 (0·41 to 3·15) | |  | 0·86 | 1·21 |  | 0·62 (0·38 to 1·01) | 3·07 | 2·24 |
| 74 |  | 2000 | Preux | 0·50 (0·30 to 0·80) |  | 1·52 | 1·28 |  |  |  |  |  |  |  |  |  |  |
| 121 |  | 2000 | Vanacore | 0·50 (0·29 to 0·87) |  | 1·31 | 1·1 |  |  |  |  |  |  |  |  |  |  |
| 91 |  | 2001 | Behari | 0·62 (0·43 to 0·91) |  | 2·06 | 1·77 |  |  |  |  |  |  |  |  |  |  |
| 62 |  | 2001 | Herishanu | 0·36 (0·19 to 0·69) |  | 1·04 | 0·86 |  |  |  |  |  |  |  |  |  |  |
| 122 |  | 2001 | Paganini-Hill | 0·85 (0·68 to 1·06) |  | 3·05 | 2·73 |  | 0·42 (0·25 to 0·69) | |  | 3·46 | 3·68 |  | 0·92 (0·73 to 1·16) | 6·31 | 5·09 |
| 141 |  | 2002 | Checkoway |  |  |  |  |  | 0·30 (0·10 to 0·70) | |  | 0·94 | 1·32 |  | 0·60 (0·40 to 0·90) | 3·87 | 2·89 |
| 82 |  | 2002 | Zorzon | 0·70 (0·40 to 1·10) |  | 1·46 | 1·23 |  |  |  |  |  |  |  |  |  |  |
| 123 |  | 2003 | Baldereschi | 0·67 (0·40 to 1·13) |  | 1·41 | 1·19 |  |  |  |  |  |  |  |  |  |  |
| 124 |  | 2003 | Baldi | 1·97 (1·02 to 3·79) |  | 1·01 | 0·84 |  |  |  |  |  |  |  |  |  |  |
| 125 |  | 2003 | Dong | 0·49 (0·30 to 0·79) |  | 1·54 | 1·3 |  | 0·44 (0·23 to 0·86) | |  | 2·05 | 2·52 |  | 0·54 (0·30 to 0·96) | 2·41 | 1·73 |
| 60 |  | 2003 | Duzcan | 0·96 (0·44 to 2·11) |  | 0·76 | 0·63 |  |  |  |  |  |  |  |  |  |  |
| 126 |  | 2003 | Pals | 0·67 (0·44 to 1·04) |  | 1·77 | 1·52 |  |  |  |  |  |  |  |  |  |  |
| 37 |  | 2003 | Ragonese | 0·66 (0·41 to 1·05) |  | 1·6 | 1·36 |  | 0·87 (0·46 to 1·65) | |  | 2·19 | 2·65 |  | 0·59 (0·26 to 1·28) | 1·46 | 1·02 |
| 248 |  | 2003 | Tan | 0·55 (0·34 to 0·90) |  | 1·53 | 1·3 |  |  |  |  |  |  |  |  |  |  |
| 92 |  | 2005 | Galanaud | 0·60 (0·40 to 0·90) |  | 1·89 | 1·63 |  | 0·50 (0·20 to 1·00) | |  | 1·38 | 1·83 |  | 0·70 (0·40 to 1·00) | 3·34 | 2·46 |
| 128 |  | 2006 | Ma | 1·63 (0·75 to 3·51) |  | 0·78 | 0·65 |  |  |  |  |  |  |  |  |  |  |
| 129 |  | 2006 | Powers | 0·53 (0·40 to 0·71) |  | 2·59 | 2·29 |  |  |  |  |  |  |  |  |  |  |
| 59 |  | 2007 | Dick | 0·48 (0·40 to 0·58) |  | 3·3 | 2·99 |  |  |  |  |  |  |  |  |  |  |
| 130 |  | 2007 | Frigerio | 0·71 (0·46 to 1·09) |  | 1·77 | 1·51 |  |  |  |  |  |  |  |  |  |  |
| 131 |  | 2007 | Kamel | 0·92 (0·65 to 1·29) |  | 2·24 | 1·95 |  | 0·38 (0·16 to 0·78) | |  | 1·42 | 1·87 |  | 1·20 (0·83 to 1·71) | 4·39 | 3·33 |
| 49 |  | 2008 | Becker | 0·54 (0·48 to 1·61) |  | 1·14 | 0·95 |  | 0·46 (0·39 to 0·54) | |  | 33·7 | 9·25 |  | 0·61 (0·53 to 0·70) | 7·84 | 6·66 |
| 132 |  | 2008 | Facheris | 0·49 (0·37 to 0·64) |  | 2·68 | 2·37 |  |  |  |  |  |  |  |  |  |  |
| 133 |  | 2008 | Petersen | 0·92 (0·53 to 1·61) |  | 1·29 | 1·08 |  | 0·63 (0·26 to 1·55) | |  | 1·12 | 1·53 |  | 1·06 (0·58 to 1·95) | 2·26 | 1·61 |
| 134 |  | 2008 | Powers | 0·77 (0·64 to 0·93) |  | 3·3 | 2·99 |  | 0·45 (0·29 to 0·70) | |  | 4·6 | 4·41 |  | 0·77 (0·64 to 0·93) | 7·06 | 5·84 |
| 135 |  | 2009 | D'Amelio | 0·86 (0·62 to 1·19) |  | 2·34 | 2·04 |  |  |  |  |  |  |  |  |  |  |
| 136 |  | 2009 | Gatto | 0·66 (0·49 to 0·90) |  | 2·48 | 2·18 |  | 0·48 (0·27 to 0·86) | |  | 2·66 | 3·06 |  | 0·70 (0·52 to 0·96) | 5·13 | 3·99 |
| 137 |  | 2009 | Tanner | 0·72 (0·56 to 0·92) |  | 2·86 | 2·55 |  |  |  |  |  |  |  |  |  |  |
| 138 |  | 2010 | Fang | 0·86 (0·75 to 0·98) |  | 3·65 | 3·35 |  | 0·51 (0·38 to 0·68) | |  | 10·54 | 6·7 |  | 0·93 (0·81 to 1·06) | 7·91 | 6·74 |
| 93 |  | 2010 | Sanyal | 0·45 (0·26 to 0·79) |  | 1·28 | 1·08 |  |  |  |  |  |  |  |  |  |  |
| 249 |  | 2010 | Skeie | 0·63 (0·42 to 0·95) |  | 1·88 | 1·61 |  |  |  |  |  |  |  |  |  |  |
| 139 |  | 2010 | Tanaka | 0·38 (0·24 to 0·60) |  | 1·65 | 1·4 |  | 0·12 (0·05 to 0·27) | |  | 1·25 | 1·69 |  | 0·51 (0·32 to 0·82) | 3·23 | 2·37 |
|  | Subtotal | |  | 0·64 (0·60 to 0·69) | | I2=48·4%, p<0·001 |  |  | 0·46 (0·41 to 0·50) | |  | I2=31·0%, p=0·068 | |  | 0·80 (0·72 to 0·89) | I2=53·2%, p=0·001 | |
|  |  |  |  |  |  |  |  |  |  |  |  |  |  |  |  |  |  |
|  | **Cohort studies** | | |  |  |  |  |  |  |  |  |  |  |  |  |  |  |
| 36 |  | 1996 | Morens | 0·40 (0·26 to 0·61) |  | 10·69 | 1·53 |  |  |  |  |  |  |  |  |  |  |
| 143 |  | 2001 | Hernan (HPFS) | 0·49 (0·35 to 0·69) |  | 13·95 | 1·97 |  | 0·30 (0·10 to 0·80) | |  | 2·85 | 1·17 |  | 0·50 (0·40 to 0·70) | 8·77 | 4·36 |
| 143 |  | 2001 | Hernan (NHS) | 0·59 (0·43 to 0·81) |  | 14·97 | 2·1 |  | 0·40 (0·20 to 0·70) | |  | 7·85 | 2·72 |  | 0·70 (0·50 to 1·00) | 5·72 | 3·5 |
| 144 |  | 2007 | Thacker | 0·71 (0·58 to 0·87) |  | 21·11 | 2·87 |  | 0·27 (0·13 to 0·56) | |  | 5·78 | 2·14 |  | 0·78 (0·64 to 0·95) | 17·6 | 5·66 |
| 142 |  | 2008 | Saaksjarvi |  |  |  |  |  | 0·19 (0·07 to 0·52) | |  | 3·06 | 1·25 |  |  |  |  |
| 145 |  | 2008 | Tan | 0·82 (0·56 to 1·19) |  | 12·42 | 1·76 |  | 0·29 (0·16 to 0·52) | |  | 8·87 | 2·98 |  | 0·77 (0·48 to 1·23) | 3·1 | 2·37 |
| 140 |  | 2009 | Chen |  |  |  |  |  | 0·60 (0·30 to 1·00) | |  | 8·5 | 2·89 |  |  |  |  |
| 146 |  | 2010 | Chen | 0·74 (0·67 to 0·82) |  | 26·86 | 3·55 |  | 0·56 (0·45 to 0·70) | |  | 63·1 | 8·07 |  | 0·78 (0·70 to 0·86) | 64·81 | 7·23 |
|  | Subtotal | |  | 0·63 (0·53 to 0·76) | | I2=63·8%, p=0·017 |  |  | 0·47 (0·40 to 0·56) | |  | I2=49·8%, p=0·063 | |  | 0·75 (0·69 to 0·81) | I2=55·1%, p=0·063 | |
|  |  |  |  |  |  |  |  |  |  |  |  |  |  |  |  |  |  |
|  | Overall | |  | 0·64 (0·60 to 0·69) | | I2=49·6%, p<0·001 |  |  | 0·44 (0·39 to 0·50) | |  | I2=33·8%, p=0·032 | |  | 0·78 (0·71 to 0·85) | I2=52·9%, p<0·001 | |
|  |  |  |  |  |  |  |  |  |  |  |  |  |  |  |  |  |  |
|  | Coffee drinking versus non-drinking | | | | | |  |  |  |  |  |  |  |  |  |  |  |
|  |  |  |  | Relative risk |  | Subgroup | Overall |  |  |  |  |  |  |  |  |  |  |
| Ref |  | Year | First author | (95% CI) |  | weight | weight |  |  |  |  |  |  |  |  |  |  |
|  | **Case-control studies** | | |  |  |  |  |  |  |  |  |  |  |  |  |  |  |
| 96 |  | 1968 | Nefzger | 0·74 (0·58 to 0·93) |  | 15·33 | 10.19 |  |  |  |  |  |  |  |  |  |  |
| 111 |  | 1992 | Jimenez-Jimenez | 0·82 (0·50 to 1·35) |  | 8·24 | 4.89 |  |  |  |  |  |  |  |  |  |  |
| 84 |  | 1994 | Morano | 0·52 (0·22 to 1·23) |  | 3·76 | 2.09 |  |  |  |  |  |  |  |  |  |  |
| 74 |  | 2000 | Preux | 0·70 (0·40 to 1·20) |  | 7·27 | 4.25 |  |  |  |  |  |  |  |  |  |  |
| 122 |  | 2001 | Paganini-Hill | 0·85 (0·70 to 1·03) |  | 16·71 | 11.38 |  |  |  |  |  |  |  |  |  |  |
| 37 |  | 2003 | Ragonese | 0·16 (0·05 to 0·46) |  | 2·44 | 1.33 |  |  |  |  |  |  |  |  |  |  |
| 147 |  | 2004 | Nuti | 0·90 (0·51 to 1·57) |  | 7·06 | 4.11 |  |  |  |  |  |  |  |  |  |  |
| 130 |  | 2007 | Frigerio | 0·40 (0·20 to 0·79) |  | 5·34 | 3.03 |  |  |  |  |  |  |  |  |  |  |
| 132 |  | 2008 | Facheris | 1·08 (0·58 to 2·01) |  | 6·16 | 3.54 |  |  |  |  |  |  |  |  |  |  |
| 134 |  | 2008 | Powers | 0·81 (0·62 to 1·06) |  | 14·29 | 9.33 |  |  |  |  |  |  |  |  |  |  |
| 135 |  | 2009 | D'Amelio | 0·39 (0·22 to 0·67) |  | 7·15 | 4.17 |  |  |  |  |  |  |  |  |  |  |
| 137 |  | 2009 | Tanner | 0·39 (0·14 to 1·09) |  | 2·79 | 1.53 |  |  |  |  |  |  |  |  |  |  |
| 249 |  | 2010 | Skeie | 0·49 (0·19 to 1·16) |  | 3·46 | 1.91 |  |  |  |  |  |  |  |  |  |  |
|  | Subtotal | |  | 0·68 (0·57 to 0·82) | | I2=47·6%, p=0·028 |  |  |  |  |  |  |  |  |  |  |  |
|  |  |  |  |  |  |  |  |  |  |  |  |  |  |  |  |  |  |
|  | **Cohort studies** | | | | | |  |  |  |  |  |  |  |  |  |  |  |
| 148 |  | 2000 | Ross | 0·45 (0·30 to 0·71) |  | 12·08 | 5.87 |  |  |  |  |  |  |  |  |  |  |
| 149 |  | 2001 | Ascherio (HPFS) | 0·70 (0·50 to 0·90) |  | 25·96 | 8.68 |  |  |  |  |  |  |  |  |  |  |
| 149 |  | 2001 | Ascherio (NHS) | 0·80 (0·60 to 1·00) |  | 34·37 | 9.67 |  |  |  |  |  |  |  |  |  |  |
| 150 |  | 2004 | Ascherio | 0·66 (0·57 to 1·23) |  | 15·16 | 6.69 |  |  |  |  |  |  |  |  |  |  |
| 151 |  | 2007 | Hu | 0·47 (0·28 to 0·80) |  | 8·14 | 4.53 |  |  |  |  |  |  |  |  |  |  |
| 142 |  | 2008 | Saaksjarvi | 0·60 (0·29 to 1·23) |  | 4·3 | 2.80 |  |  |  |  |  |  |  |  |  |  |
|  | Subtotal | |  | 0·66 (0·57 to 0·77) | | I2=29·0%, p=0·218 |  |  |  |  |  |  |  |  |  |  |  |
|  |  |  |  |  |  |  |  |  |  |  |  |  |  |  |  |  |  |
|  | Overall | |  | 0·67 (0·58 to 0·76) | | I2=42·9%, p=0·025 |  |  |  |  |  |  |  |  |  |  |  |
|  |  |  |  |  |  |  |  |  |  |  |  |  |  |  |  |  |  |
|  | Alcohol drinking versus non-drinking | | | | | |  |  |  |  |  |  |  |  |  |  |  |
|  |  |  |  | Relative risk |  | Subgroup | Overall |  |  |  |  |  |  |  |  |  |  |
| Ref |  | Year | First author | (95% CI) |  | weight | weight |  |  |  |  |  |  |  |  |  |  |
|  | **Case-control studies** | | |  |  |  |  |  |  |  |  |  |  |  |  |  |  |
| 104 |  | 1989 | Ho | 0·70 (0·20 to 2·00) |  | 0·43 | 0.38 |  |  |  |  |  |  |  |  |  |  |
| 111 |  | 1992 | Jimenez-Jimenez | 1·03 (0·66 to 1·62) |  | 2·86 | 2.48 |  |  |  |  |  |  |  |  |  |  |
| 84 |  | 1994 | Morano | 0·74 (0·40 to 1·35) |  | 1·56 | 1.35 |  |  |  |  |  |  |  |  |  |  |
| 112 |  | 1994 | Mayeux | 0·80 (0·40 to 1·50) |  | 1·32 | 1.14 |  |  |  |  |  |  |  |  |  |  |
| 115 |  | 1997 | Liou | 0·59 (0·26 to 1·33) |  | 0·86 | 0.75 |  |  |  |  |  |  |  |  |  |  |
| 118 |  | 1998 | Smargiassi | 1·90 (0·75 to 4·79) |  | 0·67 | 0.58 |  |  |  |  |  |  |  |  |  |  |
| 119 |  | 1999 | Gorell | 0·80 (0·45 to 1·47) |  | 1·64 | 1.42 |  |  |  |  |  |  |  |  |  |  |
| 91 |  | 2001 | Behari | 0·79 (0·51 to 1·21) |  | 3·09 | 2.67 |  |  |  |  |  |  |  |  |  |  |
| 122 |  | 2001 | Paganini-Hill | 0·85 (0·67 to 1·09) |  | 9·73 | 8.43 |  |  |  |  |  |  |  |  |  |  |
| 141 |  | 2002 | Checkoway | 0·89 (0·61 to 1·28) |  | 4·19 | 3.63 |  |  |  |  |  |  |  |  |  |  |
| 37 |  | 2003 | Ragonese | 0·61 (0·39 to 0·97) |  | 2·78 | 2.40 |  |  |  |  |  |  |  |  |  |  |
| 60 |  | 2003 | Duzcan | 1·78 (0·79 to 4·01) |  | 0·87 | 0.76 |  |  |  |  |  |  |  |  |  |  |
| 152 |  | 2004 | Hernan | 1·08 (0·93 to 1·26) |  | 24·98 | 21.64 |  |  |  |  |  |  |  |  |  |  |
| 92 |  | 2005 | Galanaud | 0·70 (0·50 to 1·00) |  | 4·8 | 4.15 |  |  |  |  |  |  |  |  |  |  |
| 59 |  | 2007 | Dick | 0·92 (0·74 to 1·15) |  | 11·85 | 10.27 |  |  |  |  |  |  |  |  |  |  |
| 130 |  | 2007 | Frigerio | 1·02 (0·61 to 1·71) |  | 2·17 | 1.88 |  |  |  |  |  |  |  |  |  |  |
| 131 |  | 2007 | Kamel | 1·10 (0·70 to 1·80) |  | 2·58 | 2.24 |  |  |  |  |  |  |  |  |  |  |
| 153 |  | 2009 | Brighina | 0·88 (0·68 to 1·12) |  | 9·25 | 8.02 |  |  |  |  |  |  |  |  |  |  |
| 135 |  | 2009 | D'Amelio | 0·88 (0·64 to 1·22) |  | 5·54 | 4.80 |  |  |  |  |  |  |  |  |  |  |
| 137 |  | 2009 | Tanner | 0·97 (0·56 to 1·68) |  | 1·91 | 1.65 |  |  |  |  |  |  |  |  |  |  |
| 247 |  | 2010 | Fukushima | 1·07 (0·74 to 1·53) |  | 4·37 | 3.78 |  |  |  |  |  |  |  |  |  |  |
| 249 |  | 2010 | Skeie | 0·55 (0·34 to 0·88) |  | 2·55 | 2.21 |  |  |  |  |  |  |  |  |  |  |
|  | Subtotal | |  | 0·92 (0·85 to 0·99) | | I2=13·1%, p=0·285 |  |  |  |  |  |  |  |  |  |  |  |
|  |  |  |  |  |  |  |  |  |  |  |  |  |  |  |  |  |  |
|  | **Cohort studies** | | |  |  |  |  |  |  |  |  |  |  |  |  |  |  |
| 38 |  | 1994 | Grandinetti | 0·76 (0·45 to 1·28) |  | 13·66 | 1·73 |  |  |  |  |  |  |  |  |  |  |
| 154 |  | 2003 | Hernan | 0·79 (0·64 to 0·97) |  | 86·34 | 11·54 |  |  |  |  |  |  |  |  |  |  |
|  | Subtotal | |  | 0·79 (0·65 to 0·95) | | I2=0·0%, p=0·893 |  |  |  |  |  |  |  |  |  |  |  |
|  |  |  |  |  |  |  |  |  |  |  |  |  |  |  |  |  |  |
|  | Overall | |  | 0·90 (0·84 to 0·96) | | I2=12·7%, p=0·285 |  |  |  |  |  |  |  |  |  |  |  |
|  |  |  |  |  |  |  |  |  |  |  |  |  |  |  |  |  |  |
|  | Tea drinking versus non-drinking | | | | | |  |  |  |  |  |  |  |  |  |  |  |
|  |  |  |  | Relative risk |  | Subgroup | Overall |  |  |  |  |  |  |  |  |  |  |
| Ref |  | Year | First author | (95% CI) |  | weight | weight |  |  |  |  |  |  |  |  |  |  |
|  | **Case-control studies** | | |  |  |  |  |  |  |  |  |  |  |  |  |  |  |
| 104 |  | 1989 | Ho | 0·80 (0·30 to 2·00) |  | 13.69 | 8.52 |  |  |  |  |  |  |  |  |  |  |
| 84 |  | 1994 | Morano | 2·10 (0·65 to 6·70) |  | 10.53 | 6.19 |  |  |  |  |  |  |  |  |  |  |
| 74 |  | 2000 | Preux | 1·90 (1·10 to 3·20) |  | 22.86 | 17.10 |  |  |  |  |  |  |  |  |  |  |
| 197 |  | 2003 | Chan | 0·68 (0·48 to 0·97) |  | 27.73 | 23.24 |  |  |  |  |  |  |  |  |  |  |
| 249 |  | 2010 | Skeie | 0.84 (0.54 to 1.32) |  | 25.20 | 19.87 |  |  |  |  |  |  |  |  |  |  |
|  | Subtotal | |  | 1·04 (0·66 to 1.65) | | I2=66.9%, p=0·017 | |  |  |  |  |  |  |  |  |  |  |
|  |  |  |  |  |  |  |  |  |  |  |  |  |  |  |  |  |  |
|  | **Cohort studies** | | |  |  |  |  |  |  |  |  |  |  |  |  |  |  |
| 151 |  | 2007 | Hu | 0·94 (0·69 to 1·26) |  | 100 | 25.08 |  |  |  |  |  |  |  |  |  |  |
|  | Subtotal | |  | 0·94 (0·70 to 1·27) | |  |  |  |  |  |  |  |  |  |  |  |  |
|  |  |  |  |  |  |  |  |  |  |  |  |  |  |  |  |  |  |
|  | Overall | |  | 1·00 (0·72 to 1·38) | | I2=58·7%, p=0·034 |  |  |  |  |  |  |  |  |  |  |  |
|  |  |  |  |  |  |  |  |  |  |  |  |  |  |  |  |  |  |
|  | Anxiety or depression preceding | | | | | |  |  |  |  |  |  |  |  |  |  |  |
|  |  |  |  | Relative risk |  | Subgroup | Overall |  |  |  |  |  |  |  |  |  |  |
| Ref |  | Year | First author | (95% CI) |  | weight | weight |  |  |  |  |  |  |  |  |  |  |
|  | **Case-control studies** | | |  |  |  |  |  |  |  |  |  |  |  |  |  |  |
| 155 |  | 1993 | Hubble | 2·74 (1·07 to 7·57) |  | 2·32 | 1.52 |  |  |  |  |  |  |  |  |  |  |
| 71 |  | 1998 | McCann | 1·20 (0·70 to 2·20) |  | 5·57 | 3.81 |  |  |  |  |  |  |  |  |  |  |
| 81 |  | 1999 | Taylor | 2·46 (1·25 to 4·85) |  | 4·31 | 2.89 |  |  |  |  |  |  |  |  |  |  |
| 156 |  | 2000 | Shiba | 2·40 (1·20 to 4·80) |  | 4·16 | 2.78 |  |  |  |  |  |  |  |  |  |  |
| 91 |  | 2001 | Behari | 1·30 (0·95 to 1·79) |  | 11·36 | 8.40 |  |  |  |  |  |  |  |  |  |  |
| 157 |  | 2003 | Leentjens | 2·40 (2·10 to 2·70) |  | 18·48 | 15.22 |  |  |  |  |  |  |  |  |  |  |
| 59 |  | 2007 | Dick | 1·90 (1·44 to 2·51) |  | 12·69 | 9.57 |  |  |  |  |  |  |  |  |  |  |
| 158 |  | 2009 | Alonso | 1·85 (1·25 to 2·75) |  | 9·08 | 6.50 |  |  |  |  |  |  |  |  |  |  |
| 63 |  | 2010 | Jacob | 1·42 (1·01 to 2·00) |  | 10·57 | 7.73 |  |  |  |  |  |  |  |  |  |  |
| 93 |  | 2010 | Sanyal | 1·98 (1·18 to 5·29) |  | 3·65 | 2.43 |  |  |  |  |  |  |  |  |  |  |
| 138 |  | 2010 | Fang | 2·22 (1·92 to 2·56) |  | 17·83 | 14.53 |  |  |  |  |  |  |  |  |  |  |
|  | Subtotal | |  | 1·90 (1·62 to 2·22) | | I2=58·3%, p=0·008 | |  |  |  |  |  |  |  |  |  |  |
|  |  |  |  |  |  |  |  |  |  |  |  |  |  |  |  |  |  |
|  | **Cohort studies** | | |  |  |  |  |  |  |  |  |  |  |  |  |  |  |
| 159 |  | 2003 | Weisskopf | 1·50 (1·00 to 2·10) |  | 1·1 | 7·01 |  |  |  |  |  |  |  |  |  |  |
| 160 |  | 2006 | Brandt-Christensen | 1·79 (1·72 to 1·86) |  | 98·9 | 17·63 |  |  |  |  |  |  |  |  |  |  |
|  | Subtotal | |  | 1·79 (1·72 to 1·86) | | I2=0%, p=0·353 |  |  |  |  |  |  |  |  |  |  |  |
|  |  |  |  |  |  |  |  |  |  |  |  |  |  |  |  |  |  |
|  | Overall | |  | 1·86 (1·64 to 2·11) | | I2=68·2%, p<0·001 |  |  |  |  |  |  |  |  |  |  |  |
|  |  |  |  |  |  |  |  |  |  |  |  |  |  |  |  |  |  |
|  | Constipation preceding | | | | | |  |  |  |  |  |  |  |  |  |  |  |
|  |  |  |  | Relative risk |  | Subgroup | Overall |  |  |  |  |  |  |  |  |  |  |
| Ref |  | Year | First author | (95% CI) |  | weight | weight |  |  |  |  |  |  |  |  |  |  |
|  | **Case-control studies** | | |  |  |  |  |  |  |  |  |  |  |  |  |  |  |
| 198 |  | 2009 | Savica | 2·18 (1·32 to 3·61) |  | 100 | 67·27 |  |  |  |  |  |  |  |  |  |  |
|  | Subtotal | |  | 2·18 (1·32 to 3·61) | |  |  |  |  |  |  |  |  |  |  |  |  |
|  |  |  |  |  |  |  |  |  |  |  |  |  |  |  |  |  |  |
|  | **Cohort studies** | | |  |  |  |  |  |  |  |  |  |  |  |  |  |  |
| 199 |  | 2001 | Abbott | 2·70 (1·30 to 5·50) |  | 100 | 32·73 |  |  |  |  |  |  |  |  |  |  |
|  | Subtotal | |  | 2·70 (1·30 to 5·50) | |  |  |  |  |  |  |  |  |  |  |  |  |
|  |  |  |  |  |  |  |  |  |  |  |  |  |  |  |  |  |  |
|  | Overall | |  | 2·34 (1·55 to 3·53) | | I2=0%, p=0·633 |  |  |  |  |  |  |  |  |  |  |  |
|  |  |  |  |  |  |  |  |  |  |  |  |  |  |  |  |  |  |
|  | Diabetes preceding | | | | | |  |  |  |  |  |  |  |  |  |  |  |
|  |  |  |  | Relative risk |  | Subgroup | Overall |  |  |  |  |  |  |  |  |  |  |
| Ref |  | Year | First author | (95% CI) |  | weight | weight |  |  |  |  |  |  |  |  |  |  |
|  | **Case-control studies** | | |  |  |  |  |  |  |  |  |  |  |  |  |  |  |
| 97 |  | 1972 | Kessler | 0·58 (0·30 to 1·10) |  | 10·4 | 6.48 |  |  |  |  |  |  |  |  |  |  |
| 104 |  | 1989 | Ho | 1·60 (0·50 to 5·10) |  | 4·94 | 3.08 |  |  |  |  |  |  |  |  |  |  |
| 84 |  | 1994 | Morano | 1·39 (0·63 to 3·05) |  | 8·4 | 5.23 |  |  |  |  |  |  |  |  |  |  |
| 62 |  | 2001 | Herishanu | 0·35 (0·15 to 0·75) |  | 8·19 | 5.10 |  |  |  |  |  |  |  |  |  |  |
| 129 |  | 2006 | Powers | 0·62 (0·38 to 1·01) |  | 13·31 | 8.28 |  |  |  |  |  |  |  |  |  |  |
| 161 |  | 2008 | Becker | 0·94 (0·79 to 1·13) |  | 19·45 | 12.10 |  |  |  |  |  |  |  |  |  |  |
| 135 |  | 2009 | D'Amelio | 0·40 (0·20 to 0·80) |  | 9·72 | 6.05 |  |  |  |  |  |  |  |  |  |  |
| 162 |  | 2009 | Rugbjerg | 1·10 (0·80 to 1·50) |  | 16·93 | 10.53 |  |  |  |  |  |  |  |  |  |  |
| 163 |  | 2010 | Miyake | 0·38 (0·17 to 0·79) |  | 8·66 | 5.40 |  |  |  |  |  |  |  |  |  |  |
|  | Subtotal | |  | 0·72 (0·54 to 0·97) | | I2=64·9%, p=0·004 |  |  |  |  |  |  |  |  |  |  |  |
|  |  |  |  |  |  |  |  |  |  |  |  |  |  |  |  |  |  |
|  | **Cohort studies** | | |  |  |  |  |  |  |  |  |  |  |  |  |  |  |
| 38 |  | 1994 | Grandinetti | 1·20 (0·67 to 2·12) |  | 9·8 | 7.26 |  |  |  |  |  |  |  |  |  |  |
| 151 |  | 2007 | Hu | 1·85 (1·23 to 2·80) |  | 19·22 | 9.27 |  |  |  |  |  |  |  |  |  |  |
| 165 |  | 2007 | Simon | 1·04 (0·74 to 1·46) |  | 28·17 | 10.20 |  |  |  |  |  |  |  |  |  |  |
| 166 |  | 2008 | Driver | 1·34 (1·02 to 1·77) |  | 42·81 | 11.02 |  |  |  |  |  |  |  |  |  |  |
|  | Subtotal | |  | 1·31 (1·10 to 1·57) | | I2=34·7%, p=0·204 |  |  |  |  |  |  |  |  |  |  |  |
|  |  |  |  |  |  |  |  |  |  |  |  |  |  |  |  |  |  |
|  | Overall | |  | 0·91 (0·72 to 1·15) | | I2=70·9%, p<0·001 |  |  |  |  |  |  |  |  |  |  |  |
|  |  |  |  |  |  |  |  |  |  |  |  |  |  |  |  |  |  |
|  | Cancer preceding | | | | | |  |  |  |  |  |  |  |  |  |  |  |
|  |  |  |  | Relative risk |  | Overall |  |  |  |  |  |  |  |  |  |  |  |
| Ref |  | Year | First author | (95% CI) |  | weight |  |  |  |  |  |  |  |  |  |  |  |
|  | **Case-control studies** | | |  |  |  |  |  |  |  |  |  |  |  |  |  |  |
| 97 |  | 1972 | Kessler | 0·71 (0·22 to 2·26) |  | 0.38 |  |  |  |  |  |  |  |  |  |  |  |
| 102 |  | 1987 | Rajput | 1·30 (0·70 to 2·40) |  | 1.36 |  |  |  |  |  |  |  |  |  |  |  |
| 200 |  | 2002 | Elbaz | 0·79 (0·49 to 1·27) |  | 2.27 |  |  |  |  |  |  |  |  |  |  |  |
| 201 |  | 2004 | D'Amelio | 0·40 (0·20 to 0·70) |  | 1.31 |  |  |  |  |  |  |  |  |  |  |  |
| 202 |  | 2006 | Olsen | 1·04 (0·96 to 1·12) |  | 86.75 |  |  |  |  |  |  |  |  |  |  |  |
| 129 |  | 2006 | Powers | 0·96 (0·68 to 1·36) |  | 4.29 |  |  |  |  |  |  |  |  |  |  |  |
| 203 |  | 2007 | Driver | 0·83 (0·57 to 1·21) |  | 3.64 |  |  |  |  |  |  |  |  |  |  |  |
|  |  |  |  |  |  |  |  |  |  |  |  |  |  |  |  |  |  |
|  | Overall | |  | 1·01 (0·94 to 1·09) | | I2=50·41%, p=0·060 |  |  |  |  |  |  |  |  |  |  |  |
|  |  |  |  |  |  |  |  |  |  |  |  |  |  |  |  |  |  |
|  | Hypertension preceding | | | | | |  |  |  |  |  |  |  |  |  |  |  |
|  |  |  |  | Relative risk |  | Subgroup | Overall |  |  |  |  |  |  |  |  |  |  |
| Ref |  | Year | First author | (95% CI) |  | weight | weight |  |  |  |  |  |  |  |  |  |  |
|  | **Case-control studies** | | |  |  |  |  |  |  |  |  |  |  |  |  |  |  |
| 97 |  | 1972 | Kessler | 1·06 (0·78 to 1·43) |  | 12·35 | 10.27 |  |  |  |  |  |  |  |  |  |  |
| 102 |  | 1987 | Rajput | 0·60 (0·30 to 1·10) |  | 6·99 | 5.40 |  |  |  |  |  |  |  |  |  |  |
| 104 |  | 1989 | Ho | 0·90 (0·30 to 2·40) |  | 3·74 | 2.77 |  |  |  |  |  |  |  |  |  |  |
| 84 |  | 1994 | Morano | 1·14 (0·60 to 2·15) |  | 7·13 | 5.51 |  |  |  |  |  |  |  |  |  |  |
| 71 |  | 1998 | McCann | 0·30 (0·18 to 0·42) |  | 10·26 | 8.29 |  |  |  |  |  |  |  |  |  |  |
| 62 |  | 2001 | Herishanu | 0·48 (0·26 to 0·88) |  | 7·48 | 5.82 |  |  |  |  |  |  |  |  |  |  |
| 122 |  | 2001 | Paganini-Hill | 0·71 (0·56 to 0·89) |  | 13·56 | 11.47 |  |  |  |  |  |  |  |  |  |  |
| 129 |  | 2006 | Powers | 0·95 (0·71 to 1·27) |  | 12·57 | 10.48 |  |  |  |  |  |  |  |  |  |  |
| 161 |  | 2008 | Becker | 0·83 (0·74 to 0·92) |  | 15·18 | 13.15 |  |  |  |  |  |  |  |  |  |  |
| 163 |  | 2010 | Miyake | 0·43 (0·29 to 0·64) |  | 10·74 | 8.73 |  |  |  |  |  |  |  |  |  |  |
|  | Subtotal | |  | 0·69 (0·55 to 0·87) | | I2=77·7%, p<0·001 |  |  |  |  |  |  |  |  |  |  |  |
|  |  |  |  |  |  |  |  |  |  |  |  |  |  |  |  |  |  |
|  | **Cohort studies** | | |  |  |  |  |  |  |  |  |  |  |  |  |  |  |
| 38 |  | 1994 | Grandinetti | 1·25 (0·68 to 2·28) |  | 8·25 | 5·87 |  |  |  |  |  |  |  |  |  |  |
| 165 |  | 2007 | Simon | 0·96 (0·80 to 1·15) |  | 91·75 | 12·25 |  |  |  |  |  |  |  |  |  |  |
|  | Subtotal | |  | 0·98 (0·82 to 1·17) | | I2=0·0%, p=0·413 |  |  |  |  |  |  |  |  |  |  |  |
|  |  |  |  |  |  |  |  |  |  |  |  |  |  |  |  |  |  |
|  | Overall | |  | 0·74 (0·61 to 0·90) | | I2=76·5%, p<0·001 |  |  |  |  |  |  |  |  |  |  |  |
|  |  |  |  |  |  |  |  |  |  |  |  |  |  |  |  |  |  |
|  | Gastric ulcer preceding | | | | | |  |  |  |  |  |  |  |  |  |  |  |
|  |  |  |  | Relative risk |  | Overall |  |  |  |  |  |  |  |  |  |  |  |
| Ref |  | Year | First author | (95% CI) |  | weight |  |  |  |  |  |  |  |  |  |  |  |
|  | **Case-control studies** | | |  |  |  |  |  |  |  |  |  |  |  |  |  |  |
| 97 |  | 1972 | Kessler | 0·47 (0·25 to 0·84) |  | 38·57 |  |  |  |  |  |  |  |  |  |  |  |
| 62 |  | 2001 | Herishanu | 1·83 (0·42 to 4·36) |  | 31·85 |  |  |  |  |  |  |  |  |  |  |  |
| 128 |  | 2006 | Ma | 4·08 (1·06 to 15·60) |  | 29·59 |  |  |  |  |  |  |  |  |  |  |  |
|  | Overall | |  | 1·37 (0·36 to 5·31) | | I2=81·0%, p=0·005 |  |  |  |  |  |  |  |  |  |  |  |
|  |  |  |  |  |  |  |  |  |  |  |  |  |  |  |  |  |  |
|  | Prior general anaesthetic | | | | | |  |  |  |  |  |  |  |  |  |  |  |
|  |  |  |  | Relative risk |  | Overall |  |  |  |  |  |  |  |  |  |  |  |
| Ref |  | Year | First author | (95% CI) |  | weight |  |  |  |  |  |  |  |  |  |  |  |
|  | **Case-control studies** | | |  |  |  |  |  |  |  |  |  |  |  |  |  |  |
| 105 |  | 1989 | Hofman | 1·20 (0·60 to 2·00) |  | 14.59 |  |  |  |  |  |  |  |  |  |  |  |
| 87 |  | 1996 | de Michele | 1·05 (0·79 to 2·84) |  | 13.87 |  |  |  |  |  |  |  |  |  |  |  |
| 88 |  | 1996 | Seidler | 1·30 (0·90 to 1·90) |  | 19.38 |  |  |  |  |  |  |  |  |  |  |  |
| 118 |  | 1998 | Smargiassi | 0·72 (0·37 to 1·38) |  | 13.53 |  |  |  |  |  |  |  |  |  |  |  |
| 82 |  | 2002 | Zorzon | 2·20 (1·30 to 3·80) |  | 15.91 |  |  |  |  |  |  |  |  |  |  |  |
| 59 |  | 2007 | Dick | 0·74 (0·61 to 0·91) |  | 22.72 |  |  |  |  |  |  |  |  |  |  |  |
|  | Overall | |  | 1·10 (0·77 to 1·58) | | I2=74·2%, p=0·002 |  |  |  |  |  |  |  |  |  |  |  |
|  |  |  |  |  |  |  |  |  |  |  |  |  |  |  |  |  |  |
|  | Head injury preceding | | | | | |  |  |  |  |  |  |  |  |  |  |  |
|  |  |  |  | Relative risk |  | Overall |  |  |  |  |  |  |  |  |  |  |  |
| Ref |  | Year | First author | (95% CI) |  | weight |  |  |  |  |  |  |  |  |  |  |  |
|  | **Case-control studies** | | |  |  |  |  |  |  |  |  |  |  |  |  |  |  |
| 97 |  | 1972 | Kessler | 0·97 (0·60 to 1·58) |  | 6.54 |  |  |  |  |  |  |  |  |  |  |  |
| 103 |  | 1987 | Tanner | 2·80 (1·20 to 6·72) |  | 3.43 |  |  |  |  |  |  |  |  |  |  |  |
| 105 |  | 1989 | Hofman | 1·40 (0·60 to 2·80) |  | 3.98 |  |  |  |  |  |  |  |  |  |  |  |
| 167 |  | 1991 | Factor | 2·26 (1·04 to 4·92) |  | 3.94 |  |  |  |  |  |  |  |  |  |  |  |
| 113 |  | 1995 | Martyn | 0·60 (0·29 to 1·27) |  | 4.20 |  |  |  |  |  |  |  |  |  |  |  |
| 168 |  | 1995 | Semchuk | 3·91 (1·93 to 7·89) |  | 4.46 |  |  |  |  |  |  |  |  |  |  |  |
| 87 |  | 1996 | de Michele | 2·33 (0·97 to 5·60) |  | 3.35 |  |  |  |  |  |  |  |  |  |  |  |
| 71 |  | 1998 | McCann | 1·10 (0·70 to 1·90) |  | 6.37 |  |  |  |  |  |  |  |  |  |  |  |
| 118 |  | 1998 | Smargiassi | 2·88 (0·98 to 8·49) |  | 2.46 |  |  |  |  |  |  |  |  |  |  |  |
| 120 |  | 1999 | Kuopio | 0·89 (0·55 to 1·44) |  | 6.57 |  |  |  |  |  |  |  |  |  |  |  |
| 81 |  | 1999 | Taylor | 6·23 (2·58 to 15·07) |  | 3.32 |  |  |  |  |  |  |  |  |  |  |  |
| 82 |  | 2002 | Zorzon | 1·60 (0·80 to 2·50) |  | 5.63 |  |  |  |  |  |  |  |  |  |  |  |
| 60 |  | 2003 | Duzcan | 0·84 (0·26 to 2·74) |  | 2.14 |  |  |  |  |  |  |  |  |  |  |  |
| 123 |  | 2003 | Baldereschi | 0·85 (0·45 to 1·66) |  | 4.87 |  |  |  |  |  |  |  |  |  |  |  |
| 169 |  | 2003 | Bower | 4·30 (1·20 to 5·50) |  | 4.05 |  |  |  |  |  |  |  |  |  |  |  |
| 59 |  | 2007 | Dick | 1·52 (1·23 to 1·88) |  | 9.88 |  |  |  |  |  |  |  |  |  |  |  |
| 170 |  | 2008 | Rugbjerg | 1·50 (1·40 to 1·70) |  | 10.93 |  |  |  |  |  |  |  |  |  |  |  |
| 137 |  | 2009 | Tanner | 1·25 (0·92 to 1·69) |  | 8·76 |  |  |  |  |  |  |  |  |  |  |  |
| 93 |  | 2010 | Sanyal | 2·37 (1·27 to 4·41) |  | 5·14 |  |  |  |  |  |  |  |  |  |  |  |
|  | Overall | |  | 1·58 (1·30 to 1·91) | | I2=65·6%, p<0·001 |  |  |  |  |  |  |  |  |  |  |  |
|  |  |  |  |  |  |  |  |  |  |  |  |  |  |  |  |  |  |
|  | NSAIDs use | | | | | |  |  |  |  |  |  |  |  |  |  |  |
|  |  |  |  | Relative risk |  | Subgroup | Overall |  |  |  |  |  |  |  |  |  |  |
| Ref |  | Year | First author | (95% CI) |  | weight | weight |  |  |  |  |  |  |  |  |  |  |
|  | **Case-control studies** | | |  |  |  |  |  |  |  |  |  |  |  |  |  |  |
| 223 |  | 2006 | Bower | 0·60 (0·20 to 1·70) |  | 1.07 | 1.58 |  |  |  |  |  |  |  |  |  |  |
| 226 |  | 2006 | Ton | 0·90 (0·59 to 1·35) |  | 7.16 | 7.33 |  |  |  |  |  |  |  |  |  |  |
| 227 |  | 2006 | Hernan | 0·93 (0·80 to 1·08) |  | 54.50 | 16.46 |  |  |  |  |  |  |  |  |  |  |
| 225 |  | 2007 | Wahner | 0·52 (0·35 to 0·79) |  | 7.41 | 7.49 |  |  |  |  |  |  |  |  |  |  |
| 134 |  | 2008 | Powers | 0·83 (0·68 to 1·02) |  | 29.86 | 14.25 |  |  |  |  |  |  |  |  |  |  |
|  | Subtotal | |  | 0·86 (0·77 to 0·96) | | I2=46·7%, p=0·112 |  |  |  |  |  |  |  |  |  |  |  |
|  |  |  |  |  |  |  |  |  |  |  |  |  |  |  |  |  |  |
|  | **Cohort studies** | | |  |  |  |  |  |  |  |  |  |  |  |  |  |  |
| 228 |  | 2003 | Chen | 0·55 (0·32 to 0·96) |  | 15·02 | 5·08 |  |  |  |  |  |  |  |  |  |  |
| 229 |  | 2005 | Chen | 0·78 (0·60 to 1·02) |  | 29·45 | 13·38 |  |  |  |  |  |  |  |  |  |  |
| 230 |  | 2007 | Bornebroek | 1·50 (0·95 to 2·37) |  | 18·69 | 6·77 |  |  |  |  |  |  |  |  |  |  |
| 231 |  | 2008 | Etminan | 0·84 (0·81 to 1·09) |  | 36·84 | 20·31 |  |  |  |  |  |  |  |  |  |  |
|  | Subtotal | |  | 0·86 (0·66 to 1·12) | | I2=65·8%, p=0·033 |  |  |  |  |  |  |  |  |  |  |  |
|  |  |  |  |  |  |  |  |  |  |  |  |  |  |  |  |  |  |
|  | Overall | |  | 0·83 (0·72 to 0·95) | | I2=50·9%, p=0·038 |  |  |  |  |  |  |  |  |  |  |  |
|  |  |  |  |  |  |  |  |  |  |  |  |  |  |  |  |  |  |
|  | Aspirin use | | | | | |  |  |  |  |  |  |  |  |  |  |  |
|  |  |  |  | Relative risk |  | Subgroup | Overall |  |  |  |  |  |  |  |  |  |  |
| Ref |  | Year | First author | (95% CI) |  | weight | weight |  |  |  |  |  |  |  |  |  |  |
|  | **Case-control studies** | | |  |  |  |  |  |  |  |  |  |  |  |  |  |  |
| 223 |  | 2006 | Bower | 1·40 (0·80 to 2·30) |  | 18·62 | 7·94 |  |  |  |  |  |  |  |  |  |  |
| 227 |  | 2006 | Hernan | 1·29 (1·05 to 1·58) |  | 32·69 | 22·37 |  |  |  |  |  |  |  |  |  |  |
| 226 |  | 2006 | Ton | 0·74 (0·49 to 1·12) |  | 23·15 | 11·24 |  |  |  |  |  |  |  |  |  |  |
| 225 |  | 2007 | Wahner | 0·80 (0·56 to 1·15) |  | 25·54 | 13·37 |  |  |  |  |  |  |  |  |  |  |
|  | Subtotal | |  | 1·02 (0·74 to 1·40) | | I2=69·0%, p=0·021 |  |  |  |  |  |  |  |  |  |  |  |
|  |  |  |  |  |  |  |  |  |  |  |  |  |  |  |  |  |  |
|  | **Cohort studies** | | |  |  |  |  |  |  |  |  |  |  |  |  |  |  |
| 228 |  | 2003 | Chen | 1·13 (0·92 to 1·38) |  | 49·61 | 22·48 |  |  |  |  |  |  |  |  |  |  |
| 229 |  | 2005 | Chen | 1·28 (1·05 to 1·57) |  | 50·39 | 22·59 |  |  |  |  |  |  |  |  |  |  |
|  | Subtotal | |  | 1·20 (1·04 to 1·39) | | I2=0·0%, p=0·392 |  |  |  |  |  |  |  |  |  |  |  |
|  |  |  |  |  |  |  |  |  |  |  |  |  |  |  |  |  |  |
|  | Overall | |  | 1·11 (0·93 to 1·32) | | I2=55·1%, p=0·049 |  |  |  |  |  |  |  |  |  |  |  |
|  |  |  |  |  |  |  |  |  |  |  |  |  |  |  |  |  |  |
|  | Acetaminophen/Paracetamol use | | | | | |  |  |  |  |  |  |  |  |  |  |  |
|  |  |  |  | Relative risk |  | Subgroup | Overall |  |  |  |  |  |  |  |  |  |  |
| Ref |  | Year | First author | (95% CI) |  | weight | weight |  |  |  |  |  |  |  |  |  |  |
|  | **Case-control studies** | | |  |  |  |  |  |  |  |  |  |  |  |  |  |  |
| 227 |  | 2006 | Hernan | 1·16 (1·00 to 1·35) |  | 100 | 56·21 |  |  |  |  |  |  |  |  |  |  |
|  | Subtotal | |  | 1·16 (1·00 to 1·35) | |  |  |  |  |  |  |  |  |  |  |  |  |
|  |  |  |  |  |  |  |  |  |  |  |  |  |  |  |  |  |  |
|  | **Cohort studies** | | |  |  |  |  |  |  |  |  |  |  |  |  |  |  |
| 229 |  | 2005 | Chen | 0·86 (0·66 to 1·10) |  | 100 | 43·79 |  |  |  |  |  |  |  |  |  |  |
|  | Subtotal | |  | 0·86 (0·67 to 1·11) | |  |  |  |  |  |  |  |  |  |  |  |  |
|  |  |  |  |  |  |  |  |  |  |  |  |  |  |  |  |  |  |
|  | Overall | |  | 1·02 (0·76 to 1·36) | | I2=74·5%, p=0·048 |  |  |  |  |  |  |  |  |  |  |  |
|  |  |  |  |  |  |  |  |  |  |  |  |  |  |  |  |  |  |
|  | Statin use | | | | | |  |  |  |  |  |  |  |  |  |  |  |
|  |  |  |  | Relative risk |  | Overall |  |  |  |  |  |  |  |  |  |  |  |
| Ref |  | Year | First author | (95% CI) |  | weight |  |  |  |  |  |  |  |  |  |  |  |
|  | **Case-control studies** | | |  |  |  |  |  |  |  |  |  |  |  |  |  |  |
| 212 |  | 2007 | Huang | 0·37 (0·19 to 0·72) |  | 9·73 |  |  |  |  |  |  |  |  |  |  |  |
| 234 |  | 2008 | Wahner | 0·45 (0·29 to 0·71) |  | 15·20 |  |  |  |  |  |  |  |  |  |  |  |
| 232 |  | 2008 | Samii | 0·94 (0·82 to 1·09) |  | 26·05 |  |  |  |  |  |  |  |  |  |  |  |
| 49 |  | 2008 | Becker | 1·11 (0·94 to 1·30) |  | 25·44 |  |  |  |  |  |  |  |  |  |  |  |
| 233 |  | 2010 | Ritz | 0·89 (0·72 to 1·11) |  | 23·57 |  |  |  |  |  |  |  |  |  |  |  |
|  | Overall | |  | 0·79 (0·61 to 1·02) | | I2=82·0%, p<0·001 |  |  |  |  |  |  |  |  |  |  |  |
|  |  |  |  |  |  |  |  |  |  |  |  |  |  |  |  |  |  |
|  | Hormone replacement therapy | | | | | |  |  |  |  |  |  |  |  |  |  |  |
|  |  |  |  | Relative risk |  | Subgroup | Overall |  |  |  |  |  |  |  |  |  |  |
| Ref |  | Year | First author | (95% CI) |  | weight | weight |  |  |  |  |  |  |  |  |  |  |
|  | **Case-control studies** | | |  |  |  |  |  |  |  |  |  |  |  |  |  |  |
| 235 |  | 1998 | Marder | 1·02 (0·56 to 1·80) |  | 18·64 | 11·83 |  |  |  |  |  |  |  |  |  |  |
| 236 |  | 2001 | Benedetti | 0·47 (0·12 to 1·85) |  | 3·4 | 3·82 |  |  |  |  |  |  |  |  |  |  |
| 237 |  | 2003 | Martignoni | 0·62 (0·40 to 0·98) |  | 31·65 | 14·64 |  |  |  |  |  |  |  |  |  |  |
| 126 |  | 2003 | Pals | 0·53 (0·13 to 2·11) |  | 3·27 | 3·70 |  |  |  |  |  |  |  |  |  |  |
| 238 |  | 2004 | Currie | 0·40 (0·19 to 0·84) |  | 11·5 | 9·17 |  |  |  |  |  |  |  |  |  |  |
| 239 |  | 2004 | Ragonese | 0·45 (0·13 to 1·50) |  | 4·25 | 4·58 |  |  |  |  |  |  |  |  |  |  |
| 240 |  | 2005 | Popat | 1·30 (0·80 to 2·10) |  | 27·29 | 13·89 |  |  |  |  |  |  |  |  |  |  |
|  | Subtotal | |  | 0·77 (0·60 to 0·99) | | I2=44·5%, p=0·094 |  |  |  |  |  |  |  |  |  |  |  |
|  |  |  |  |  |  |  |  |  |  |  |  |  |  |  |  |  |  |
|  | **Cohort studies** | | |  |  |  |  |  |  |  |  |  |  |  |  |  |  |
| 150 |  | 2004 | Ascherio | 1·33 (1·07 to 1·67) |  | 59·68 | 19·69 |  |  |  |  |  |  |  |  |  |  |
| 241 |  | 2009 | Simon | 1·25 (0·96 to 1·65) |  | 40·32 | 18·68 |  |  |  |  |  |  |  |  |  |  |
|  | Subtotal | |  | 1·30 (1·09 to 1·54) | | I2=0·0%, p=0·729 |  |  |  |  |  |  |  |  |  |  |  |
|  |  |  |  |  |  |  |  |  |  |  |  |  |  |  |  |  |  |
|  | Overall | |  | 0·90 (0·67 to 1·21) | | I2=63·9%, p=0·005 |  |  |  |  |  |  |  |  |  |  |  |
|  |  |  |  |  |  |  |  |  |  |  |  |  |  |  |  |  |  |
|  | Oophorectomy preceding | | | | | |  |  |  |  |  |  |  |  |  |  |  |
|  |  |  |  | Relative risk |  | Subgroup | Overall |  |  |  |  |  |  |  |  |  |  |
| Ref |  | Year | First author | (95% CI) |  | weight | weight |  |  |  |  |  |  |  |  |  |  |
|  | **Case-control studies** | | |  |  |  |  |  |  |  |  |  |  |  |  |  |  |
| 236 |  | 2001 | Benedetti | 2·23 (0·90 to 5·54) |  | 20·62 | 12·36 |  |  |  |  |  |  |  |  |  |  |
| 237 |  | 2003 | Martignoni | 0·80 (0·50 to 1·30) |  | 30·52 | 23·86 |  |  |  |  |  |  |  |  |  |  |
| 239 |  | 2004 | Ragonese | 0·30 (0·13 to 0·77) |  | 21·01 | 12·72 |  |  |  |  |  |  |  |  |  |  |
| 240 |  | 2005 | Popat | 0·70 (0·40 to 1·30) |  | 27·85 | 20·13 |  |  |  |  |  |  |  |  |  |  |
|  | Subtotal | |  | 0·77 (0·42 to 1·43) | | I2=69·1%, p=0·021 |  |  |  |  |  |  |  |  |  |  |  |
|  |  |  |  |  |  |  |  |  |  |  |  |  |  |  |  |  |  |
|  | **Cohort studies** | | |  |  |  |  |  |  |  |  |  |  |  |  |  |  |
| 241 |  | 2009 | Simon | 0·75 (0·56 to 0·99) |  | 100 | 30·94 |  |  |  |  |  |  |  |  |  |  |
|  | Subtotal | |  | 0·75 (0·56 to 1·00) | |  |  |  |  |  |  |  |  |  |  |  |  |
|  |  |  |  |  |  |  |  |  |  |  |  |  |  |  |  |  |  |
|  | Overall | |  | 0·76 (0·52 to 1·13) | | I2=58·8%, p=0·046 |  |  |  |  |  |  |  |  |  |  |  |
|  |  |  |  |  |  |  |  |  |  |  |  |  |  |  |  |  |  |
|  | Oral contraceptive pill use | | | | | |  |  |  |  |  |  |  |  |  |  |  |
|  |  |  |  | Relative risk |  | Subgroup | Overall |  |  |  |  |  |  |  |  |  |  |
| Ref |  | Year | First author | (95% CI) |  | weight | weight |  |  |  |  |  |  |  |  |  |  |
|  | **Case-control studies** | | |  |  |  |  |  |  |  |  |  |  |  |  |  |  |
| 237 |  | 2003 | Martignoni | 0·41 (0·22 to 0·76) |  | 50·52 | 29·16 |  |  |  |  |  |  |  |  |  |  |
| 240 |  | 2005 | Popat | 0·80 (0·40 to 1·40) |  | 49·48 | 28·93 |  |  |  |  |  |  |  |  |  |  |
|  | Subtotal | |  | 0·57 (0·37 to 0·89) | | I2=54·8%, p=0·137 |  |  |  |  |  |  |  |  |  |  |  |
|  |  |  |  |  |  |  |  |  |  |  |  |  |  |  |  |  |  |
|  | **Cohort studies** | | |  |  |  |  |  |  |  |  |  |  |  |  |  |  |
| 241 |  | 2009 | Simon | 1·02 (0·77 to 1·36) |  | 100 | 41·91 |  |  |  |  |  |  |  |  |  |  |
|  | Subtotal | |  | 1·02 (0·77 to 1·36) | |  |  |  |  |  |  |  |  |  |  |  |  |
|  |  |  |  |  |  |  |  |  |  |  |  |  |  |  |  |  |  |
|  | Overall | |  | 0·73 (0·43 to 1·25) | | I2=71·1%, p=0·031 |  |  |  |  |  |  |  |  |  |  |  |
|  |  |  |  |  |  |  |  |  |  |  |  |  |  |  |  |  |  |
|  | Calcium channel blocker use | | | | | |  |  |  |  |  |  |  |  |  |  |  |
|  |  |  |  | Relative risk |  | Subgroup | Overall |  |  |  |  |  |  |  |  |  |  |
| Ref |  | Year | First author | (95% CI) |  | weight | weight |  |  |  |  |  |  |  |  |  |  |
|  | **Case-control studies** | | |  |  |  |  |  |  |  |  |  |  |  |  |  |  |
| 242 |  | 2007 | Ton | 0·85 (0·43 to 1·66) |  | 2·19 | 2·10 |  |  |  |  |  |  |  |  |  |  |
| 161 |  | 2008 | Becker | 0·91 (0·82 to 1·02) |  | 83·82 | 80·39 |  |  |  |  |  |  |  |  |  |  |
| 243 |  | 2009 | Louis | 1·13 (0·59 to 2·17) |  | 2·35 | 2·26 |  |  |  |  |  |  |  |  |  |  |
| 244 |  | 2010 | Ritz | 0·73 (0·54 to 0·97) |  | 11·64 | 11·16 |  |  |  |  |  |  |  |  |  |  |
|  | Subtotal | |  | 0·89 (0·81 to 0·98) | | I2=0%, p=0·484 |  |  |  |  |  |  |  |  |  |  |  |
|  |  |  |  |  |  |  |  |  |  |  |  |  |  |  |  |  |  |
|  | **Cohort studies** | | |  |  |  |  |  |  |  |  |  |  |  |  |  |  |
| 245 |  | 2010 | Simon | 1·18 (0·73 to 1·92) |  | 100 | 4·09 |  |  |  |  |  |  |  |  |  |  |
|  | Subtotal | |  | 1·18 (0·73 to 1·91) | |  |  |  |  |  |  |  |  |  |  |  |  |
|  |  |  |  |  |  |  |  |  |  |  |  |  |  |  |  |  |  |
|  | Overall | |  | 0·90 (0·82 to 0·99) | | I2=0%, p=0·447 |  |  |  |  |  |  |  |  |  |  |  |
|  |  |  |  |  |  |  |  |  |  |  |  |  |  |  |  |  |  |
|  | Beta blocker use | | |  |  |  |  |  |  |  |  |  |  |  |  |  |  |
|  |  |  |  | Relative risk |  | Overall |  |  |  |  |  |  |  |  |  |  |  |
| Ref |  | Year | First author | (95% CI) |  | weight |  |  |  |  |  |  |  |  |  |  |  |
|  | **Case-control studies** | | |  |  |  |  |  |  |  |  |  |  |  |  |  |  |
| 242 |  | 2007 | Ton | 1·20 (0·71 to 2·03) |  | 2·22 |  |  |  |  |  |  |  |  |  |  |  |
| 161 |  | 2008 | Becker | 1·28 (1·16 to 1·41) |  | 64·20 |  |  |  |  |  |  |  |  |  |  |  |
| 244 |  | 2010 | Ritz | 1·29 (1·13 to 1·48) |  | 33·59 |  |  |  |  |  |  |  |  |  |  |  |
|  | Overall | |  | 1·28 (1·19 to 1·39) | | I2=0·0%, p=0·966 |  |  |  |  |  |  |  |  |  |  |  |
|  |  |  |  |  |  |  |  |  |  |  |  |  |  |  |  |  |  |
|  | Pesticide exposure | | | | | |  |  |  |  |  |  |  |  |  |  |  |
|  |  |  |  | Relative risk |  | Subgroup | Overall |  |  |  |  |  |  |  |  |  |  |
| Ref |  | Year | First author | (95% CI) |  | weight | weight |  |  |  |  |  |  |  |  |  |  |
|  | **Case-control studies** | | |  |  |  |  |  |  |  |  |  |  |  |  |  |  |
| 104 |  | 1989 | Ho | 3·60 (1·00 to 12·90) |  | 1·4 | 1·28 |  |  |  |  |  |  |  |  |  |  |
| 171 |  | 1990 | Golbe | 7·00 (1·61 to 63·46) |  | 0·79 | 0·72 |  |  |  |  |  |  |  |  |  |  |
| 172 |  | 1990 | Koller | 1·05 (0·67 to 1·65) |  | 3·77 | 3·58 |  |  |  |  |  |  |  |  |  |  |
| 111 |  | 1992 | Jimenez-Jimenez | 1·34 (0·85 to 2·12) |  | 3·75 | 3·55 |  |  |  |  |  |  |  |  |  |  |
| 155 |  | 1993 | Hubble | 3·42 (1·27 to 7·32) |  | 2·26 | 2·10 |  |  |  |  |  |  |  |  |  |  |
| 173 |  | 1994 | Hertzman | 2·06 (1·11 to 3·85) |  | 3·1 | 2·91 |  |  |  |  |  |  |  |  |  |  |
| 84 |  | 1994 | Morano | 1·73 (0·95 to 3·15) |  | 3·18 | 2·99 |  |  |  |  |  |  |  |  |  |  |
| 174 |  | 1995 | Chaturvedi | 1·81 (0·92 to 3·36) |  | 3.00 | 2·82 |  |  |  |  |  |  |  |  |  |  |
| 168 |  | 1995 | Semchuk | 3·09 (1·27 to 7·56) |  | 2·21 | 2·06 |  |  |  |  |  |  |  |  |  |  |
| 88 |  | 1996 | Seidler | 1·70 (1·00 to 2·60) |  | 3·67 | 3·47 |  |  |  |  |  |  |  |  |  |  |
| 115 |  | 1997 | Liou | 2·89 (2·28 to 3·66) |  | 4·58 | 4·39 |  |  |  |  |  |  |  |  |  |  |
| 89 |  | 1998 | Chan | 0·75 (0·26 to 2·22) |  | 1·76 | 1·63 |  |  |  |  |  |  |  |  |  |  |
| 117 |  | 1998 | de Palma | 2·92 (1·38 to 6·14) |  | 2·66 | 2·48 |  |  |  |  |  |  |  |  |  |  |
| 71 |  | 1998 | McCann | 1·20 (0·80 to 1·50) |  | 4·31 | 4·12 |  |  |  |  |  |  |  |  |  |  |
| 175 |  | 1999 | Fall | 2·80 (0·89 to 8·70) |  | 1·64 | 1·51 |  |  |  |  |  |  |  |  |  |  |
| 120 |  | 1999 | Kuopio | 1·02 (0·63 to 1·65) |  | 3·65 | 3·46 |  |  |  |  |  |  |  |  |  |  |
| 81 |  | 1999 | Taylor | 1·02 (0·90 to 1·17) |  | 4·85 | 4·66 |  |  |  |  |  |  |  |  |  |  |
| 90 |  | 1999 | Werneck | 2·49 (0·53 to 13·14) |  | 0·99 | 0·90 |  |  |  |  |  |  |  |  |  |  |
| 62 |  | 2001 | Herishanu | 6·81 (0·75 to 64·89) |  | 0·56 | 0·51 |  |  |  |  |  |  |  |  |  |  |
| 82 |  | 2002 | Zorzon | 1·60 (1·00 to 2·40) |  | 3·83 | 3·63 |  |  |  |  |  |  |  |  |  |  |
| 123 |  | 2003 | Baldereschi | 3·68 (1·57 to 8·64) |  | 2·32 | 2·16 |  |  |  |  |  |  |  |  |  |  |
| 124 |  | 2003 | Baldi | 2·20 (1·10 to 4·30) |  | 2·88 | 2.70 |  |  |  |  |  |  |  |  |  |  |
| 60 |  | 2003 | Duzcan | 2·96 (1·31 to 6·69) |  | 2·44 | 2·27 |  |  |  |  |  |  |  |  |  |  |
| 176 |  | 2004 | Gorell | 4·10 (1·37 to 12·23) |  | 1·73 | 1·59 |  |  |  |  |  |  |  |  |  |  |
| 147 |  | 2004 | Nuti | 0·94 (0·62 to 1·43) |  | 3·91 | 3·71 |  |  |  |  |  |  |  |  |  |  |
| 92 |  | 2005 | Galanaud | 1·60 (1·00 to 2·40) |  | 3·83 | 3·63 |  |  |  |  |  |  |  |  |  |  |
| 177 |  | 2006 | Frigerio | 1·50 (0·80 to 2·90) |  | 3·02 | 2·83 |  |  |  |  |  |  |  |  |  |  |
| 59 |  | 2007 | Dick | 1·25 (0·97 to 1·61) |  | 4·52 | 4·33 |  |  |  |  |  |  |  |  |  |  |
| 178 |  | 2007 | Fong | 1·68 (1·03 to 2·76) |  | 3·6 | 3·41 |  |  |  |  |  |  |  |  |  |  |
| 131 |  | 2007 | Kamel | 1·30 (0·50 to 3·30) |  | 2·08 | 1·92 |  |  |  |  |  |  |  |  |  |  |
| 133 |  | 2008 | Petersen | 6·00 (0·62 to 57·68) |  | 0·55 | 0·50 |  |  |  |  |  |  |  |  |  |  |
| 137 |  | 2009 | Tanner | 1·90 (1·12 to 3·21) |  | 3·47 | 3·28 |  |  |  |  |  |  |  |  |  |  |
| 179 |  | 2010 | Firestone | 0·77 (0·37 to 1·55) |  | 2·76 | 2·58 |  |  |  |  |  |  |  |  |  |  |
| 93 |  | 2010 | Sanyal | 17·12 (4·97 to 58·84) | | 1·47 | 1·35 |  |  |  |  |  |  |  |  |  |  |
| 249 |  | 2010 | Skeie | 1·06 (0·60 to 1·89) |  | 3·28 | 3·09 |  |  |  |  |  |  |  |  |  |  |
| 180 |  | 2010 | Hristina | 3·22 (1·32 to 7·87) |  | 2·21 | 2·05 |  |  |  |  |  |  |  |  |  |  |
|  | Subtotal | |  | 1·77 (1·48 to 2·12) | | I2=73·3%, p<0·001 |  |  |  |  |  |  |  |  |  |  |  |
|  |  |  |  |  |  |  |  |  |  |  |  |  |  |  |  |  |  |
|  | **Cohort studies** | | |  |  |  |  |  |  |  |  |  |  |  |  |  |  |
| 181 |  | 2003 | Baldi | 2·70 (0·98 to 7·43) |  | 9·35 | 1·76 |  |  |  |  |  |  |  |  |  |  |
| 182 |  | 2006 | Ascherio | 1·70 (1·20 to 2·30) |  | 90·65 | 4·08 |  |  |  |  |  |  |  |  |  |  |
|  | Subtotal | |  | 1·78 (1·30 to 2·42) | | I2=0·0%, p=0·394 |  |  |  |  |  |  |  |  |  |  |  |
|  |  |  |  |  |  |  |  |  |  |  |  |  |  |  |  |  |  |
|  | Overall | |  | 1·78 (1·50 to 2·10) | | I2=72·4%, p<0·001 |  |  |  |  |  |  |  |  |  |  |  |
|  |  |  |  |  |  |  |  |  |  |  |  |  |  |  |  |  |  |
|  | Farming or agricultural occupation | | | | | |  |  |  |  |  |  |  |  |  |  |  |
|  |  |  |  | Relative risk |  | Subgroup | Overall |  |  |  |  |  |  |  |  |  |  |
| Ref |  | Year | First author | (95% CI) |  | weight | weight |  |  |  |  |  |  |  |  |  |  |
|  | **Case-control studies** | | |  |  |  |  |  |  |  |  |  |  |  |  |  |  |
| 104 |  | 1989 | Ho | 1·70 (0·68 to 4·12) |  | 1·96 | 1·90 |  |  |  |  |  |  |  |  |  |  |
| 172 |  | 1990 | Koller | 1·39 (0·88 to 2·19) |  | 5·21 | 5·15 |  |  |  |  |  |  |  |  |  |  |
| 183 |  | 1991 | Semchuk | 0·90 (0·58 to 1·37) |  | 5·57 | 5·52 |  |  |  |  |  |  |  |  |  |  |
| 109 |  | 1991 | Wechsler | 3·10 (0·30 to 35·00) |  | 0·32 | 0·31 |  |  |  |  |  |  |  |  |  |  |
| 173 |  | 1994 | Hertzman | 0·81 (0·44 to 1·47) |  | 3·64 | 3·57 |  |  |  |  |  |  |  |  |  |  |
| 84 |  | 1994 | Morano | 1·15 (0·63 to 2·08) |  | 3·69 | 3·62 |  |  |  |  |  |  |  |  |  |  |
| 184 |  | 1996 | Rocca | 0·60 (0·30 to 1·30) |  | 2·73 | 2·66 |  |  |  |  |  |  |  |  |  |  |
| 88 |  | 1996 | Seidler | 0·70 (0·40 to 1·10) |  | 4·6 | 4·53 |  |  |  |  |  |  |  |  |  |  |
| 115 |  | 1997 | Liou | 1·81 (1·25 to 2·64) |  | 6·42 | 6·39 |  |  |  |  |  |  |  |  |  |  |
| 89 |  | 1998 | Chan | 0·92 (0·59 to 1·43) |  | 5·39 | 5·33 |  |  |  |  |  |  |  |  |  |  |
| 175 |  | 1999 | Fall | 1·40 (0·78 to 5·70) |  | 1·65 | 1.60 |  |  |  |  |  |  |  |  |  |  |
| 120 |  | 1999 | Kuopio | 1·45 (0·88 to 2·41) |  | 4·62 | 4·56 |  |  |  |  |  |  |  |  |  |  |
| 91 |  | 2001 | Behari | 1·35 (0·92 to 2·02) |  | 6·11 | 6·07 |  |  |  |  |  |  |  |  |  |  |
| 185 |  | 2001 | Kirkey | 1·74 (0·85 to 3·60) |  | 2·8 | 2·72 |  |  |  |  |  |  |  |  |  |  |
| 82 |  | 2002 | Zorzon | 7·70 (1·40 to 44·10) |  | 0·6 | 0·58 |  |  |  |  |  |  |  |  |  |  |
| 60 |  | 2003 | Duzcan | 1·69 (0·78 to 3·65) |  | 2·52 | 2·45 |  |  |  |  |  |  |  |  |  |  |
| 186 |  | 2005 | Frigerio | 1·50 (0·80 to 2·50) |  | 3·94 | 3·87 |  |  |  |  |  |  |  |  |  |  |
| 92 |  | 2005 | Galanaud | 1·90 (1·20 to 2·90) |  | 5·41 | 5·35 |  |  |  |  |  |  |  |  |  |  |
| 187 |  | 2005 | Park | 1·88 (1·12 to 3·15) |  | 4·48 | 4·41 |  |  |  |  |  |  |  |  |  |  |
| 188 |  | 2007 | Dick | 1·02 (0·82 to 1·28) |  | 9·25 | 9·38 |  |  |  |  |  |  |  |  |  |  |
| 137 |  | 2009 | Tanner | 1·10 (0·78 to 1·57) |  | 6·82 | 6·81 |  |  |  |  |  |  |  |  |  |  |
| 179 |  | 2010 | Firestone | 1·13 (0·84 to 1·53) |  | 7·72 | 7·76 |  |  |  |  |  |  |  |  |  |  |
| 249 |  | 2010 | Skeie | 1·75 (1·03 to 3·00) |  | 4·29 | 4.22 |  |  |  |  |  |  |  |  |  |  |
| 93 |  | 2010 | Sanyal | 2·01 (0·14 to 27·93) |  | 0·26 | 0·25 |  |  |  |  |  |  |  |  |  |  |
|  | Subtotal | |  | 1·26 (1·10 to 1·45) | | I2=39·5%, p=0·030 |  |  |  |  |  |  |  |  |  |  |  |
|  |  |  |  |  |  |  |  |  |  |  |  |  |  |  |  |  |  |
|  | **Cohort studies** | | |  |  |  |  |  |  |  |  |  |  |  |  |  |  |
| 181 |  | 2003 | Baldi | 1·24 (0·34 to 4·53) |  | 100 | 0·99 |  |  |  |  |  |  |  |  |  |  |
|  | Subtotal | |  | 1·24 (0·34 to 4·53) | | I^2^=100% |  |  |  |  |  |  |  |  |  |  |  |
|  |  |  |  |  |  |  |  |  |  |  |  |  |  |  |  |  |  |
|  | Overall | |  | 1·26 (1·10 to 1·44) | | I2=36·9%, p=0·034 |  |  |  |  |  |  |  |  |  |  |  |
|  |  |  |  |  |  |  |  |  |  |  |  |  |  |  |  |  |  |
|  | Well water exposure | | | | | |  |  |  |  |  |  |  |  |  |  |  |
|  |  |  |  | Relative risk |  | Overall |  |  |  |  |  |  |  |  |  |  |  |
| Ref |  | Year | First author | (95% CI) |  | weight |  |  |  |  |  |  |  |  |  |  |  |
|  | **Case-control studies** | | |  |  |  |  |  |  |  |  |  |  |  |  |  |  |
| 189 |  | 1989 | Tanner | 0·74 (0·41 to 1·32) |  | 3·29 |  |  |  |  |  |  |  |  |  |  |  |
| 171 |  | 1990 | Golbe | 1·33 (0·65 to 2·80) |  | 2·54 |  |  |  |  |  |  |  |  |  |  |  |
| 172 |  | 1990 | Koller | 1·70 (1·05 to 2·70) |  | 4·03 |  |  |  |  |  |  |  |  |  |  |  |
| 183 |  | 1991 | Semchuk | 1·07 (0·57 to 2·02) |  | 3·02 |  |  |  |  |  |  |  |  |  |  |  |
| 109 |  | 1991 | Wechsler | 1·27 (0·39 to 4·11) |  | 1·26 |  |  |  |  |  |  |  |  |  |  |  |
| 111 |  | 1992 | Jimenez-Jimenez | 1·22 (0·77 to 1·94) |  | 4·10 |  |  |  |  |  |  |  |  |  |  |  |
| 83 |  | 1993 | Wang | 0·59 (0·36 to 0·95) |  | 3·94 |  |  |  |  |  |  |  |  |  |  |  |
| 173 |  | 1994 | Hertzman | 0·80 (0·35 to 1·86) |  | 2·13 |  |  |  |  |  |  |  |  |  |  |  |
| 84 |  | 1994 | Morano | 4·98 (1·13 to 45·27) |  | 0·58 |  |  |  |  |  |  |  |  |  |  |  |
| 87 |  | 1996 | de Michele | 2·17 (1·28 to 3·69) |  | 3·64 |  |  |  |  |  |  |  |  |  |  |  |
| 88 |  | 1996 | Seidler | 0·90 (0·60 to 1·30) |  | 4·68 |  |  |  |  |  |  |  |  |  |  |  |
| 115 |  | 1997 | Liou | 1·07 (0·19 to 5·98) |  | 0·65 |  |  |  |  |  |  |  |  |  |  |  |
| 89 |  | 1998 | Chan | 1·04 (0·70 to 1·54) |  | 4·62 |  |  |  |  |  |  |  |  |  |  |  |
| 190 |  | 1998 | Gorell | 0·97 (0·65 to 1·40) |  | 4·70 |  |  |  |  |  |  |  |  |  |  |  |
| 71 |  | 1998 | McCann | 0·60 (0·38 to 0·92) |  | 4·25 |  |  |  |  |  |  |  |  |  |  |  |
| 118 |  | 1998 | Smargiassi | 2·78 (1·46 to 5·28) |  | 2·96 |  |  |  |  |  |  |  |  |  |  |  |
| 117 |  | 1998 | de Palma | 2·09 (1·27 to 3·42) |  | 3·86 |  |  |  |  |  |  |  |  |  |  |  |
| 81 |  | 1999 | Taylor | 0·93 (0·88 to 0·98) |  | 6·89 |  |  |  |  |  |  |  |  |  |  |  |
| 90 |  | 1999 | Werneck | 1·49 (0·74 to 3·01) |  | 2·67 |  |  |  |  |  |  |  |  |  |  |  |
| 91 |  | 2001 | Behari | 1·20 (0·88 to 1·64) |  | 5·28 |  |  |  |  |  |  |  |  |  |  |  |
| 82 |  | 2002 | Zorzon | 2·00 (1·10 to 3·60) |  | 3·24 |  |  |  |  |  |  |  |  |  |  |  |
| 147 |  | 2004 | Nuti | 0·93 (0·58 to 1·48) |  | 4·06 |  |  |  |  |  |  |  |  |  |  |  |
| 191 |  | 2004 | Park | 0·62 (0·29 to 1·32) |  | 2·42 |  |  |  |  |  |  |  |  |  |  |  |
| 192 |  | 2005 | Firestone | 1·08 (0·77 to 1·50) |  | 5·10 |  |  |  |  |  |  |  |  |  |  |  |
| 59 |  | 2007 | Dick | 1·23 (1·00 to 1·52) |  | 6·08 |  |  |  |  |  |  |  |  |  |  |  |
| 136 |  | 2009 | Gatto | 1·21 (0·82 to 1·80) |  | 4·62 |  |  |  |  |  |  |  |  |  |  |  |
| 180 |  | 2010 | Hristina | 2·62 (1·40 to 4·90) |  | 3·05 |  |  |  |  |  |  |  |  |  |  |  |
| 93 |  | 2010 | Sanyal | 4·50 (2·10 to 9·90) |  | 2·35 |  |  |  |  |  |  |  |  |  |  |  |
|  | Overall | |  | 1·21 (1·04 to 1·40) | | I2=70·6%, p<0·001 |  |  |  |  |  |  |  |  |  |  |  |
|  |  |  |  |  |  |  |  |  |  |  |  |  |  |  |  |  |  |
|  | Rural living | | | | | |  |  |  |  |  |  |  |  |  |  |  |
|  |  |  |  | Relative risk |  | Subgroup | Overall |  |  |  |  |  |  |  |  |  |  |
| Ref |  | Year | First author | (95% CI) |  | weight | weight |  |  |  |  |  |  |  |  |  |  |
|  | **Case-control studies** | | |  |  |  |  |  |  |  |  |  |  |  |  |  |  |
| 104 |  | 1989 | Ho | 2·19 (0·90 to 5·61) |  | 3·63 | 3·47 |  |  |  |  |  |  |  |  |  |  |
| 171 |  | 1990 | Golbe | 2·00 (1·04 to 4·00) |  | 4·72 | 4·54 |  |  |  |  |  |  |  |  |  |  |
| 172 |  | 1990 | Koller | 1·90 (1·17 to 3·02) |  | 5·76 | 5·56 |  |  |  |  |  |  |  |  |  |  |
| 183 |  | 1991 | Semchuk | 0·78 (0·51 to 1·21) |  | 5·98 | 5·77 |  |  |  |  |  |  |  |  |  |  |
| 111 |  | 1992 | Jimenez-Jimenez | 1·07 (0·68 to 1·67) |  | 5·89 | 5·68 |  |  |  |  |  |  |  |  |  |  |
| 83 |  | 1993 | Wang | 0·76 (0·49 to 1·18) |  | 5·94 | 5·73 |  |  |  |  |  |  |  |  |  |  |
| 84 |  | 1994 | Morano | 0·69 (0·35 to 1·32) |  | 4·77 | 4·58 |  |  |  |  |  |  |  |  |  |  |
| 88 |  | 1996 | Seidler | 0·90 (0·60 to 1·20) |  | 6·4 | 6·19 |  |  |  |  |  |  |  |  |  |  |
| 115 |  | 1997 | Liou | 2·04 (1·23 to 3·38) |  | 5·59 | 5·39 |  |  |  |  |  |  |  |  |  |  |
| 71 |  | 1998 | McCann | 1·70 (1·20 to 2·60) |  | 6·2 | 6.00 |  |  |  |  |  |  |  |  |  |  |
| 117 |  | 1998 | de Palma | 3·62 (2·09 to 6·26) |  | 5·37 | 5·17 |  |  |  |  |  |  |  |  |  |  |
| 118 |  | 1998 | Smargiassi | 2·50 (1·33 to 4·55) |  | 5·02 | 4·83 |  |  |  |  |  |  |  |  |  |  |
| 90 |  | 1999 | Werneck | 1·00 (0·52 to 1·95) |  | 4·79 | 4·60 |  |  |  |  |  |  |  |  |  |  |
| 74 |  | 2000 | Preux | 1·67 (1·00 to 2·50) |  | 5·84 | 5·64 |  |  |  |  |  |  |  |  |  |  |
| 91 |  | 2001 | Behari | 0·88 (0·66 to 1·18) |  | 6·65 | 6·44 |  |  |  |  |  |  |  |  |  |  |
| 82 |  | 2002 | Zorzon | 1·50 (1·00 to 2·40) |  | 5·95 | 5·74 |  |  |  |  |  |  |  |  |  |  |
| 147 |  | 2004 | Nuti | 0·95 (0·59 to 1·53) |  | 5·74 | 5·54 |  |  |  |  |  |  |  |  |  |  |
| 93 |  | 2010 | Sanyal | 4·05 (2·53 to 6·49) |  | 5·77 | 5·57 |  |  |  |  |  |  |  |  |  |  |
|  | Subtotal | |  | 1·43 (1·12 to 1·83) | | I2=78·9%, p<0·001 |  |  |  |  |  |  |  |  |  |  |  |
|  |  |  |  |  |  |  |  |  |  |  |  |  |  |  |  |  |  |
|  | **Cohort studies** | | |  |  |  |  |  |  |  |  |  |  |  |  |  |  |
| 181 |  | 2003 | Baldi | 1·37 (0·56 to 3·33) |  | 100 | 3·75 |  |  |  |  |  |  |  |  |  |  |
|  | Subtotal | |  | 1·37 (0·56 to 3·34) | |  |  |  |  |  |  |  |  |  |  |  |  |
|  |  |  |  |  |  |  |  |  |  |  |  |  |  |  |  |  |  |
|  | Overall | |  | 1·43 (1·13 to 1·81) | | I2=77·7%, p<0·001 |  |  |  |  |  |  |  |  |  |  |  |
