# Supplementary material for: Meta-Analysis of Early Nonmotor Features and Risk Factors for Parkinson Disease
Source: Ann Neurol. 2012 Oct 15;72(6):893–901. doi: 10.1002/ana.23687 (PMC3556649; doi:10.1002/ana.23687)
Supplement: Supplementary file 4 [file ana0072-0893-SD4.doc]

|  | **Table 3 - Studies included for systematic review but not suitable for use in the meta-analysis** | | | | | | | | | | |  |  |
| --- | --- | --- | --- | --- | --- | --- | --- | --- | --- | --- | --- | --- | --- |
|  |  |  |  |  |  |  |  |  |  |  |  |  |  |
| **Ref** | **Factor and reason not in meta-analysis** | | | **First Author** | **Year** | **Cohort/Case-control design** | |  |  | **OR/RR** | **CI** |  | **Primary study finding** |
|  |  |  |  |  |  |  |  |  |  |  |  |  |  |
| 85 | Family History - single study | | | Bonifati | 1995 | Case-control study | |  |  | 7.63 | 3.67-15.79 |  | OR of PD if family history of PD or tremor |
| 155 | Family History - single study | | | Hubble | 1993 | Case-control study | |  |  | 3.18 | 1.22-7.05 |  | aOR of PD given a family history of neurological disease |
|  |  |  |  |  |  |  |  |  |  |  |  |  |  |
| 193 | Smoking - single study | | | O'Reilly | 2009 | Cohort study |  |  |  | 0.73 | 0.53-1.00 |  | RR of PD if both parents smoked |
| 194 | Smoking - single study | | | O'Reilly | 2005 | Cohort study |  |  |  | 0.24 | 0.08-0.75 |  | aRR for current vs never smokers of smokeless tobacco |
|  |  |  |  |  |  |  |  |  |  |  |  |  |  |
| 195 | Coffee - could not convert for meta-analysis | | | Hellenbrand | 1996 | Case-control study | |  |  | 0.27 | 0.14-0.52 |  | aOR of PD if prior coffee consumption in the highest quartile |
| 127 | Coffee - could not convert for meta-analysis | | | Tan | 2003 | Case-control study | |  |  | 0.78 | 0.66-0.93 |  | Multivariate OR of PD for coffee >3 cups/day for 10 years |
| 196 | Coffee - could not convert for meta-analysis | | | Tan | 2007 | Case-control study | |  |  | 0.47 | 0.34-0.65 |  | OR of PD given high coffee drinking |
| 38 | Coffee - could not convert for meta-analysis | | | Grandinetti | 1994 | Cohort study |  |  |  | 0.46 | 0.26-0.82 |  | RR of PD if regular coffee drinking |
| 122 | Coffee - could not convert for meta-analysis | | | Paganini-Hill | 2001 | Population-based case-control study |  |  |  | 0.64 | 0.48-0.84 |  | OR of PD if >2 cups of coffee per day |
| 175 | Coffee - could not convert for meta-analysis | | | Fall | 1999 | Case-control study | |  |  | 0.13 | 0.03-0.44 |  | aOR of PD if >5 cups of coffee per day |
| 141 | Coffee - could not convert for meta-analysis | | | Checkoway | 2002 | Case-control study | |  |  | 1 | 0.5-2.0 |  | aOR of PD for coffee >6 cups/day |
| 145 | Coffee - could not convert for meta-analysis | | | Tan | 2008 | Cohort study |  |  |  | 1.04 | 0.53-2.03 |  | aRR of PD for coffee >2 cups/day |
|  |  |  |  |  |  |  |  |  |  |  |  |  |  |
| 175 | Alcohol - could not convert for meta-analysis | | | Fall | 1999 | Case-control study | |  |  | 0.47 | 0.26-0.83 |  | aOR of PD if 1-3 medium strength bottles of beer per day (also wine and liquor studied) |
| 83 | Alcohol - could not convert for meta-analysis | | | Wang | 1993 | Case-control study | |  |  | 0.6 | 0.36-0.99 |  | OR of PD given drinking hard liquor |
| 195 | Alcohol - could not convert for meta-analysis | | | Hellenbrand | 1996 | Case-control study | |  |  | 0.26 | 0.14-0.49 |  | aOR of PD given highest quartile of beer (also wine and spirits studied) |
|  |  |  |  |  |  |  |  |  |  |  |  |  |  |
| 127 | Tea - could not convert for meta-analysis | | | Tan | 2003 | Case-control study | |  |  | 0.72 | 0.56-0.94 |  | Multivariate OR of PD for tea >3 cups/day for 10 years |
| 141 | Tea - could not convert for meta-analysis | | | Checkoway | 2002 | Case-control study | |  |  | 0.4 | 0.20-0.90 |  | aOR of PD for tea >2 cups/day |
| 175 | Tea - could not convert for meta-analysis | | | Fall | 1999 | Case-control study | |  |  | 0.31 | 0.10-0.87 |  | aOR of PD for tea 2-5 cups/day |
| 145 | Tea - could not convert for meta-analysis | | | Tan | 2008 | Cohort study |  |  |  | 0.29 | 0.13-0.67 |  | aRR of PD for highest vs lowest quartile of black tea consumption |
| 195 | Tea - could not convert for meta-analysis | | | Hellenbrand | 1996 | Case-control study | |  |  | 0.82 | 0.52-1.29 |  | aOR of PD given highest quartile of tea |
| 122 | Tea - could not convert for meta-analysis | | | Paganini-Hill | 2001 | Population-based case control study |  |  |  | 1.21 | 0.86-1.70 |  | OR of PD for tea >2 cups/day |
| 99 | Tea - could not convert for meta-analysis | | | Baumann | 1980 | Case-control study | |  |  | 0.98 | 0.78-1.22 |  | OR of PD if tea or coffee consumption |
|  |  |  |  |  |  |  |  |  |  |  |  |  |  |
| 42 | Smell - single study | | | Ross | 2008 | Cohort study |  |  |  | 5.2 | 1.5-25.6 |  | Adjusted OR for smell sense in the lowest quartile vs the top two quartiles |
|  |  |  |  |  |  |  |  |  |  |  |  |  |  |
| 44 | Erectile dysfunction - single study | | | Gao | 2007 | Cohort study |  |  |  | 3.8 | 2.4-6.0 |  | aRR of PD given preceding ED |
|  |  |  |  |  |  |  |  |  |  |  |  |  |  |
| 43 | Excessive daytime somnolence - single study | | | Abbott | 2005 | Cohort study |  |  |  | 2.8 | 1.1-6.4 |  | aOR 2.8 of PD in men with excessive daytime somnolence |
|  |  |  |  |  |  |  |  |  |  |  |  |  |  |
| 47 | Subjective complaints - single study | | | de Lau | 2006 | Cohort study |  |  |  | 2.11 | 1.25-3.55 |  | aHR of PD given preceding subjective complaint of stiffness (also imbalance and tremor) |
| 48 | Subjective complaints - single study | | | Gao | 2008 | Cohort study |  |  |  | 1.8 | 1.3-2.5 |  | aRR of PD for with preceding imbalance |
|  |  |  |  |  |  |  |  |  |  |  |  |  |  |
| 204 | Cancer - single study | |  | Gao | 2009 | Cohort study |  |  |  | 1.85 | 1.2-2.8 |  | aRR of PD if 1st degree relative with melanoma |
| 205 | Cancer - single study | |  | Gao | 2009 | Cohort study |  |  |  | 1.93 | 1.08-3.42 |  | aRR of PD given red hair |
|  |  |  |  |  |  |  |  |  |  |  |  |  |  |
| 210 | Serum urate/uric acid – different measurement of urate/uric acid - could not convert for meta-analysis | | | Alonso | 2007 | Case-control study | |  |  | 0.69 | 0.48-0.99 |  | aOR of PD given preceding gout |
| 207 | Serum urate/uric acid – different measurement of urate/uric acid - could not convert for meta-analysis | | | Weisskopf | 2007 | Case-control study | |  |  | 0.43 | 0.18-1.02 |  | aRR of PD for highest vs lowest quartile of serum urate |
| 208 | Serum urate/uric acid – different measurement of urate/uric acid - could not convert for meta-analysis | | | Davis | 1996 | Cohort study |  |  |  | 0.6 | 0.4-1.0 |  | aRR of PD if uric acid level above the median |
| 209 | Serum urate/uric acid – different measurement of urate/uric acid - could not convert for meta-analysis | | | de Lau | 2005 | Cohort study |  |  |  | 0.71 | 0.51-0.98 |  | aHR for PD per standard deviation increase in uric acid level |
| 206 | Serum urate/uric acid – different measurement of urate/uric acid - could not convert for meta-analysis | | | O'Reilly | 2010 | Case-control study | |  |  | 1.33 | 0.69-2.57 |  | aRR of PD for highest vs lowest quartile of serum urate |
| 140 | Serum urate/uric acid – different measurement of urate/uric acid - could not convert for meta-analysis | | | Chen | 2009 | Cohort study |  |  |  | 0.4 | 0.2-0.8 |  | aRR of PD for highest vs lowest quartile of serum urate |
|  |  |  |  |  |  |  |  |  |  |  |  |  |  |
| 211 | Cholesterol – could not convert for meta-analysis | | | Hu | 2008 | Cohort study |  |  |  | 1.86 | 1.31-2.63 |  | aHR of PD in highest vs lowest total cholesterol quintile |
| 221 | Cholesterol – could not convert for meta-analysis | | | de Lau | 2006 | Cohort study |  |  |  | 0.77 | 0.64-0.94 |  | aHR for every 1mmol/L increase in serum total cholesterol |
| 212 | Cholesterol – could not convert for meta-analysis | | | Huang | 2007 | Case-control study | |  |  | 2.6 | 1.1- 6.0 |  | aOR given lowest LDL-C quartile (also total cholesterol (non-significant)) |
| 163 | Cholesterol – could not convert for meta-analysis | | | Miyake | 2010 | Case-control study | |  |  | 0.58 | 0.33-0.97 |  | aOR of PD if hypercholesterolaemia |
| 213 | Cholesterol – could not convert for meta-analysis | | | Huang | 2008 | Cohort study |  |  |  | 0.4 | 0.2-0.9 |  | aRR of PD given highest vs lowest LDL-C percentile |
| 49 | Hyperlipidaemia – could not convert for meta-analysis | | | Becker | 2008 | Case-control study | |  |  | 1.07 | 0.9-1.29 |  | aOR of PD if hyperlipidaemia |
| 38 | Cholesterol – could not convert for meta-analysis | | | Grandinetti | 1994 | Cohort study |  |  |  | 0.73 | 0.43-1.24 |  | aRR of PD if high cholesterol |
| 165 | Cholesterol – could not convert for meta-analysis | | | Simon | 2007 | Cohort study |  |  |  | 0.98 | 0.82-1.19 |  | aRR of PD if high cholesterol |
|  |  |  |  |  |  |  |  |  |  |  |  |  |  |
| 214 | Obesity – could not convert for meta-analysis | | | Abbott | 2002 | Cohort study |  |  |  | 2.8 | 1.4-5.6 |  | aRR of PD in highest vs lowest quartile of triceps skin thickness |
| 215 | Obesity – could not convert for meta-analysis | | | Hu | 2006 | Cohort study |  |  |  | 2.03 | 1.44-2.85 |  | aRR of PD in highest vs lowest quintile of body mass index |
| 128 | Obesity – could not convert for meta-analysis | | | Ma | 2006 | Population-based case-control study |  |  |  | 0.43 | 0.2-0.9 |  | aRR of PD given BMI >23 |
| 161 | Obesity – could not convert for meta-analysis | | | Becker | 2008 | Case-control study | |  |  | 0.9 | 0.75-1.07 |  | aOR of PD given highest tertile of BMI |
| 216 | Obesity – could not convert for meta-analysis | | | Chen | 2004 | Cohort study |  |  |  | 0.8 | 0.6-1.2 |  | aRR of PD given highest quintile of BMI |
| 217 | Obesity – could not convert for meta-analysis | | | Logroscino | 2007 | Cohort study |  |  |  | 0.86 | 0.53-1.41 |  | aRR of PD given highest tertile of BMI |
| 218 | Obesity – could not convert for meta-analysis | | | Ragonese | 2008 | Case-control study | |  |  | 0.99 | 0.94-1.03 |  | aOR of PD given high BMI |
|  |  |  |  |  |  |  |  |  |  |  |  |  |  |
| 219 | Physical activity – could not convert for meta-analysis | | | Xu | 2010 | Cohort study |  |  |  | 0.6 | 0.37-0.95 |  | aOR of PD given highest vs lowest quartile of activity |
| 220 | Physical activity – could not convert for meta-analysis | | | Sasco | 1992 | Population-based case-control study |  |  |  | 0.85 | 0.31-2.3 |  | aOR of PD in highest weekly exercise quintile |
|  |  |  |  |  |  |  |  |  |  |  |  |  |  |
| 113 | Infections - single study | | | Martyn | 1995 | Case-control study | |  |  | 2.3 | 1.2-4.7 |  | OR of PD given previous diphtheria infection (also croup and rheumatic fever significant) |
| 120 | Infections - single study | | | Kuopio | 1999 | Population-based case-control study |  |  |  | 0.37 | 0.16-0.90 |  | OR of PD given prior rubella infection |
| 97 | Infections - single study | | | Kessler | 1972 | Case-control study | |  |  | 4.15 | - |  | OR of PD given encephalitis (females) |
| 222 | Infections - single study | | | Sasco | 1985 | Population-based case-control study |  |  |  | 0.53 | 0.31-0.93 |  | OR of PD given childhood measles |
|  |  |  |  |  |  |  |  |  |  |  |  |  |  |
| 223 | Other preceding diagnoses - single study | | | Bower | 2006 | Case-control study | |  |  | 1.8 | 1.1-3.1 |  | OR of PD given preceding immediate-type hypersensitivity |
| 224 | Other preceding diagnoses - single study | | | Savica | 2009 | Case-control study | |  |  | 2.17 | 1.40-3.37 |  | aOR of PD if anaemia or low haemoglobin |
|  |  |  |  |  |  |  |  |  |  |  |  |  |  |
| 239 | Reproductive factors - single study | | | Ragonese | 2004 | Case-control study | |  |  | 2.19 | 1.22-3.91 |  | aOR of PD if cumulative length of pregnancies longer than 30 months (also length of fertile life significant) |
| 122 | Reproductive factors - single study | | | Paganini-Hill | 2001 | Population-based case-control study |  |  |  | 1.87 | 1.36-2.58 |  | OR of PD given 3 or more children |
| 126 | Reproductive factors - single study | | | Pals | 2003 | Case-control study | |  |  | 2.49 | 1.08-5.76 |  | OR of PD if no children |
|  |  |  |  |  |  |  |  |  |  |  |  |  |  |
| 122 | Antihypertensive medication - sub-categories in meta-analysis | | | Paganini-Hill | 2001 | Population-based case-control study |  |  |  | 0.63 | 0.49-0.80 |  | OR of PD if preceding use of anti-hypertensive medication |
| 165 | Antihypertensive medication - sub-categories in meta-analysis | | | Simon | 2007 | Cohort study |  |  |  | 1.15 | 0.95-1.39 |  | aRR of PD if use of anti-hypertensive drugs |
|  |  |  |  |  |  |  |  |  |  |  |  |  |  |
| 63 | Education - could not convert for meta-analysis | | | Jacob | 2010 | Population-based case-control study |  |  |  | 0.56 | 0.39-0.79 |  | OR of PD if greater than 12 years of education |
| 81 | Education - could not convert for meta-analysis | | | Taylor | 1999 | Case-control study | |  |  | 0.79 | 0.71-0.88 |  | aOR of PD given years of education |
| 92 | Education - could not convert for meta-analysis | | | Galanaud | 2005 | Population-based case-control study |  |  |  | 0.6 | 0.4-0.9 |  | OR of PD given education higher than primary school |
| 186 | Education - could not convert for meta-analysis | | | Frigerio | 2005 | Case-control study | |  |  | 2.0 | 1.1-3.6 |  | OR for PD if >9 years of education |
| 60 | Education - could not convert for meta-analysis | | | Duzcan | 2003 | Case-control study | |  |  | 1.89 | 0.85-4.22 |  | OR of PD given illiteracy |
| 113 | Education - could not convert for meta-analysis | | | Martyn | 1995 | Case-control study | |  |  | 0.8 | 0.52-1.35 |  | OR of PD given leaving school at >14 yrs |
| 93 | Education - could not convert for meta-analysis | | | Sanyal | 2010 | Case-control study | |  |  | 1.58 | 0.58-4.31 |  | OR of PD given illiteracy |
| 184 | Education - could not convert for meta-analysis | | | Rocca | 1996 | Case-control study | |  |  | 1.9 | 0.9-4.4 |  | OR of PD given illiteracy |
| 120 | Education - could not convert for meta-analysis | | | Kuopio | 1999 | Population-based case-control study |  |  |  | 0.86 | 0.44-1.69 |  | OR of PD given low grade of education |
|  |  |  |  |  |  |  |  |  |  |  |  |  |  |
| 137 | Occupation - could not convert for meta-analysis | | | Tanner | 2009 | Case-control study | |  |  | 2.95 | 1.34-6.48 |  | aOR of PD given a legal occupation (construction/extraction also significant) |
| 186 | Occupation - could not convert for meta-analysis | | | Frigerio | 2005 | Case-control study | |  |  | 8.9 | 1.1-72.1 |  | OR of PD given occupation involving health diagnosing and treating practitioners (contruction/extraction, production workers, metal workers, engineers and physicians also significant) |
| 185 | Occupation - could not convert for meta-analysis | | | Kirkey | 2001 | Population-based case-control study |  |  |  | 0.69 | 0.47-1.00 |  | OR of PD given service-sector occupation |
| 188 | Occupation - could not convert for meta-analysis | | | Dick | 2007 | Case-control study | |  |  | 0.6 | 0.37-0.97 |  | aOR of PD given transport or communication (processing occupations also significant) |
| 187 | Occupation - could not convert for meta-analysis | | | Park | 2005 | Case-control study | |  |  | 0.28 | 0.09-0.87 |  | aOR of PD given transportation occupation (manufacturing, drivers, service occupation and technicians also significant) |
| 191 | Occupation - could not convert for meta-analysis | | | Park | 2004 | Case-control study | |  |  | 0.36 | 0.14-0.94 |  | aOR of PD given sales occupation (clerk occupation also significant) |
| 175 | Occupation - could not convert for meta-analysis | | | Fall | 1999 | Case-control study | |  |  | 6.7 | 1.76-30.0 |  | OR of PD given cleaning occupation in females (male carpenters also significant) |
| 62 | Occupation - could not convert for meta-analysis | | | Herishanu | 2001 | Case-control study | |  |  | 2.94 | 1.08-7.99 |  | aOR of PD given work in construction |
| 180 | Occupation - could not convert for meta-analysis | | | Hristina | 2010 | Case-control study | |  |  | 0.15 | 0.04-0.59 |  | aOR of PD given work in service-sector |
